# Genes Encoding Heat Shock Proteins Are Associated with Risk and Clinical Course of Severe COVID-19: A Pilot Study

**DOI:** 10.3390/ijms26188967

**Published:** 2025-09-15

**Authors:** Andrey R. Karpenko, Ksenia A. Kobzeva, Yuriy L. Orlov, Olga Yu. Bushueva

**Affiliations:** 1 Laboratory of Genomic Research, Research Institute for Genetic and Molecular Epidemiology, Kursk State Medical University, 305041 Kursk, Russia; 2 Department of Anesthesia and Critical Care, Institute of Continuing Education, Kursk State Medical University, 305004 Kursk, Russia; 3 Institute of Biodesign and Complex Systems Modeling, Sechenov First Moscow State Medical University of the Russian Ministry of Health (Sechenov University), 119991 Moscow, Russia; 4 Agrarian and Technological Institute, Patrice Lumumba Peoples’ Friendship University of Russia, 117198 Moscow, Russia; 5 Department of Biology, Medical Genetics and Ecology, Kursk State Medical University, 305041 Kursk, Russia

**Keywords:** COVID-19, HSP, DNAJA2, HSPA12B, HSPA8, BAG3, HSPA6, HSPA9, thromboinflammation, ground-glass opacity

## Abstract

In viral infections human heat shock proteins (HSPs) play a dual role by either protecting host cells or acting on viruses’ needs. The roles of HSPs have been extensively studied in various human pathologies, but their involvement in the progression of COVID-19 remains unexplored. It makes HSPs genetic variants particularly interesting in the context of severe COVID-19 risk. In this study, 1228 subjects (199 hospitalized COVID-19 patients and 962 controls) were genotyped for 20 SNPs in genes encoding *HSPs* and their regulators. SNP rs7189628 *DNAJA2* (effect allele [EA] T) increased the risk of severe COVID-19 in the entire group (*p* = 0.002), males (*p* = 0.00008), and smokers (*p* = 0.0003). SNP rs910652 *HSPA12B* (EA C) decreased the risk of severe COVID-19 in the entire group (*p* = 0.01), females (*p* = 0.04), and patients with normal physical activity levels (*p* = 0.01). SNP rs1136141 *HSPA8* (EA A) increased the risk of severe COVID-19 in patients with low fruit/vegetable intake (*p* = 0.004). Moreover, we observed significant changes in ground-glass opacity and alterations in blood coagulation and inflammation parameters, influenced by the SNPs of *BAG3*, *HSF2*, *HSPA6*, *HSPA8*, *HSPA9*, and *DNAJA2*. The molecular mechanisms underlying these associations are discussed. Together, our study provides preliminary evidence that SNPs of HSPs can significantly modulate the risk of severe COVID-19.

## 1. Introduction

In December 2019, a novel coronavirus, SARS-CoV-2, emerged, leading to the declaration of the COVID-19 pandemic by the World Health Organization (WHO) on 11 March 2020. This virus causes a wide spectrum of clinical manifestations, ranging from asymptomatic cases to severe pneumonia, acute respiratory distress syndrome (ARDS), and multiple organ failure (MOF) [1,2]. According to the latest WHO data, COVID-19 has resulted in approximately seven million deaths worldwide, with over 775 million confirmed cases globally, including 21.4 million in the Russian Federation [3].

The complex ladder of host reactions in COVID-19 involves heat shock proteins (HSPs), which form an essential molecular machinery responsible for maintaining cellular homeostasis, particularly under stress conditions [4,5]. Their primary functions are related to proteostasis, including the folding of newly synthesized polypeptides, the refolding of misfolded proteins, the assembly of protein complexes, the degradation of damaged proteins, and the disaggregation of protein aggregates [6]. While the roles of HSPs [7,8,9] and proteins with chaperone-like activity [10,11,12] have been extensively studied in various human pathologies, their involvement in the progression and severity of COVID-19 remains largely unexplored.

Several studies have suggested that SARS-CoV-2 might trigger a hyperautoimmune response through molecular mimicry, where viral proteins share similarities with human proteins [13,14,15]. Specifically, researchers have identified immunogenic epitopes shared between coronavirus and human HSPs, which are involved in the development of autoimmune diseases [16,17]. For example, studies by Lucchese and Flöel revealed that SARS-CoV-2 molecules have affinities for human HSP60 and HSP90, suggesting a potential pathogenetic mechanism for postinfection neuropathy [17].

Further investigations revealed that heat shock factor 1 (HSF1) and HSP70 can reduce the levels of the proinflammatory cytokines TNFα, IL-1, IL-10, and IL-12 but do not affect IL-6 [18]. Additionally, a positive correlation was found between the level of heat shock protein qp96 in the blood plasma of COVID-19 patients and the severity of the disease, as indicated by the level of IL-6 [19]. HSP27 has also been identified as a predictor of exacerbation in chronic obstructive pulmonary disease (COPD), a condition similar to severe COVID-19 [20]. Moreover, recent study found that spike proteins of SARS-CoV-2 can interact with human HSPs (HSPA8 and HSP27 specifically) [21].

Understanding the molecular basis and genetic determinants of severe COVID-19 is essential for uncovering the complex interplay between SARS-CoV-2 and the human immune system, as well as for assessing the potential long-term effects of the disease. While genome-wide association studies (GWASs) have significantly advanced our knowledge of genetic factors contributing to severe COVID-19 [22,23,24] and the critical role of HSPs in the disease course is increasingly recognized, no genetic studies to date have specifically investigated the involvement of genetic variations in HSPs in the risk of severe COVID-19.

Therefore, the objective of this pilot study was to investigate the association between common single nucleotide polymorphisms (SNPs) within HSPs and the risk of severe COVID-19 in the Russian population. Additionally, this study aimed to explore the most significant gene-gene interactions related to severe COVID-19, assess the combined influence of genetic variations and environmental risk factors on disease susceptibility, and examine how HSPs loci impact the clinical features of the disease, including thrombodynamic parameters.

## 2. Results

### 2.1. Quality Control and Post Hoc Power Analysis

Table S1 presents the quality control (QC) for genotyping, including missing genotype rates, call rates, and cluster plots. Call rates ranged from 0.980 for rs7189628 *DNAJA2* to 0.998 for rs13161158 *HSAP4*.

The next step in QC included assessment of Hardy–Weinberg equilibrium (HWE) in controls and patients, heterozygosity, and minor allele frequencies (Table S2). We relied on the control group’s Hardy-Weinberg equilibrium analysis results because correlations between genetic markers and disease can cause deviations from equilibrium. With the exception of rs706121 *BAG1* (*p* = 0.03), all the examined SNPs presented genotype frequencies within the control group that were in line with HWE (*p* > 0.05). However, rs706121 *BAG1* was included in the statistical analysis because repeated genotyping of this SNP demonstrated 100% reproducibility of the primary results.

**Figure 1:**
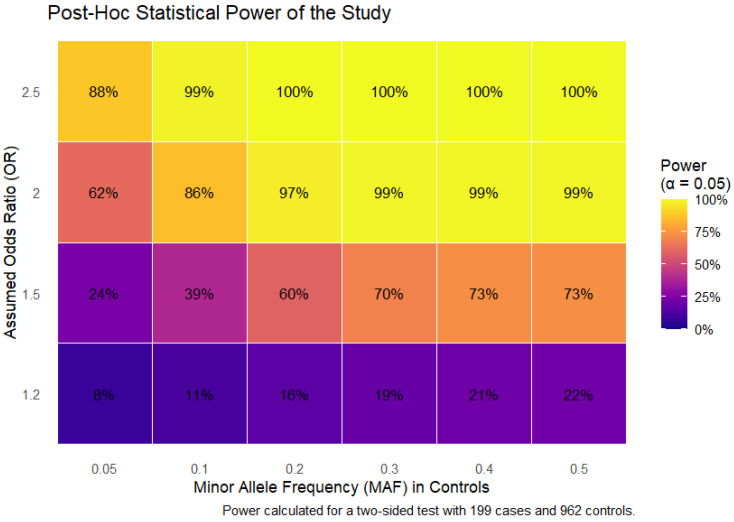
Post hoc statistical power of the genetic association study. The heatmap depicts the statistical power (percentages in cells) to detect a significant association (*p* < 0.05) across a range of assumed odds ratios (effect size) and minor allele frequencies (MAF) in controls. Power was calculated for a two-sided test with a sample size of 199 cases and 962 controls.

The results of the post hoc power analysis are presented in Figure 1. The study demonstrated high power (>80%) to detect common genetic variants (MAF ≥ 0.2) with moderate to large effects (OR ≥ 1.8). Power was more limited for detecting smaller effect sizes (OR < 1.5) or for rarer variants (MAF < 0.1). For instance, the power to detect an OR of 1.5 at a MAF of 0.05 was approximately 24%.

### 2.2. HSPs SNPs and the Risk of Severe COVID-19

We identified significant associations between certain SNPs and the risk of developing a severe course of COVID-19 within the entire patient cohort. Specifically, SNP rs7189628 *DNAJA2* (risk allele T, OR = 2.02, 95% CI 1.26–3.24, *p* = 0.003, *p*_perm_ = 0.002) was found to increase the risk of severe COVID-19, whereas SNP rs910652 *HSPA12B* (protective allele C, OR = 0.70, 95% CI 0.53–0.92, *p* = 0.01, *p*_perm_ = 0.01) was associated with a reduced risk (Table 1).

Upon further analysis stratified by sex, we observed that in males, rs7189628 *DNAJA2* (risk allele T, OR = 3.53, 95% CI 1.9–6.56, *p* = 6.8 × 10^−5^, *p*_perm_ = 7.6 × 10^−5^) significantly increased the risk of severe COVID-19. Conversely, in females, rs910652 *HSPA12B* (protective allele C, OR = 0.68, 95% CI 0.47–0.98, *p* = 0.04, *p*_perm_ = 0.04) was associated with a decreased risk of severe COVID-19 (Table 2 and Table S3).

Moreover, rs7189628 *DNAJA2* increased the risk of severe COVID-19 in smokers (risk allele T, OR = 3.99, 95% CI 1.92–8.29, *p* = 0.0002, *p*_perm_ = 0.0002) (Table 2 and Table S3), as well as in patients with normal physical activity levels (OR = 2.71, 95% CI 1.52–4.84, *p* = 0.0007, *p*_perm_ = 0.0009 (*p*_bonf_ = 0.002)) (Table 2 and Table S3).

In patients with low levels of fresh fruit and vegetable intake, rs7189628 *DNAJA2* (risk allele T, OR = 2.39, 95% CI 1.45–3.95, *p* = 0.0007, *p*_perm_ = 0.0008, (*p*_bonf_ = 0.002)) and rs1136141 *HSPA8* (risk allele A, OR = 1.69, 95% CI 1.2–2.36, *p* = 0.002, *p*_perm_ = 0.002 (*p*_bonf_ = 0.004)) increased the risk of severe COVID-19 (Table 2 and Table S3). However, rs1042665 *HSPA9* was associated with an increased risk of severe COVID-19 in the group with normal fresh fruit and vegetable intake (risk allele C, OR = 1.67, 95% CI 1.14–2.46, *p* = 0.009, *p*_perm_ = 0.009 (*p*_bonf_ = 0.02)) (Table 2 and Table S3).

Notably, for rs910652 *HSPA12B* was observed protective effects against severe COVID-19 in patients with normal physical activity levels: (protective allele C, OR = 0.58, 95% CI 0.39–0.88, *p* = 0.009, *p*_perm_ = 0.007, (*p*_bonf_ = 0.01)) (Table 2 and Table S3).

Of note, we observed sub-significant risk effects for rs7189628 *DNAJA2* in patients with low physical activity levels (OR = 1.88, 95% CI 1.05–3.34, *p* = 0.03, *p*_perm_ = 0.02, (*p*_bonf_ = 0.04)) and for rs1042665 *HSPA9* in patients with normal physical activity (risk allele C, OR = 1.47, 95% CI 1.03–2.1, *p* = 0.03, *p*_perm_ = 0.02, (*p*_bonf_ = 0.41)).

Additionally, we investigated the effects of SNPs stratified by age (Table 2 and Table S3). We found that in patients aged 68 years or older, the SNPs rs1461496 *HSPA8* (risk allele A, OR = 1.59, 95% CI 1.05–2.4, *p* = 0.03, *p*_perm_ = 0.03), rs1043618 *HSPA1A* (risk allele C, OR = 1.56, 95% CI 1.04–2.35, *p* = 0.03, *p*_perm_ = 0.035), and rs6457452 *HSPA1B* (risk allele T, OR = 2.29, 95% CI 1.16–4.54, *p* = 0.02, *p*_perm_ = 0.01) increased the risk of severe COVID-19. Conversely, in patients under 68 years old, SNPs rs1136141 *HSPA8* (risk allele A, OR = 1.55, 95% CI 1.06–2.28, *p* = 0.02, *p*_perm_ = 0.02) and rs7189628 *DNAJA2* (risk allele T, OR = 2.02, 95% CI 1.08–3.75, *p* = 0.03, *p*_perm_ = 0.02) increased the risk of severe COVID-19 (Table 2 and Table S3).

### 2.3. Associations Between HSPs SNPs and the Clinical and Biochemical Parameters of Patients with Severe COVID-19

**Figure 2:**
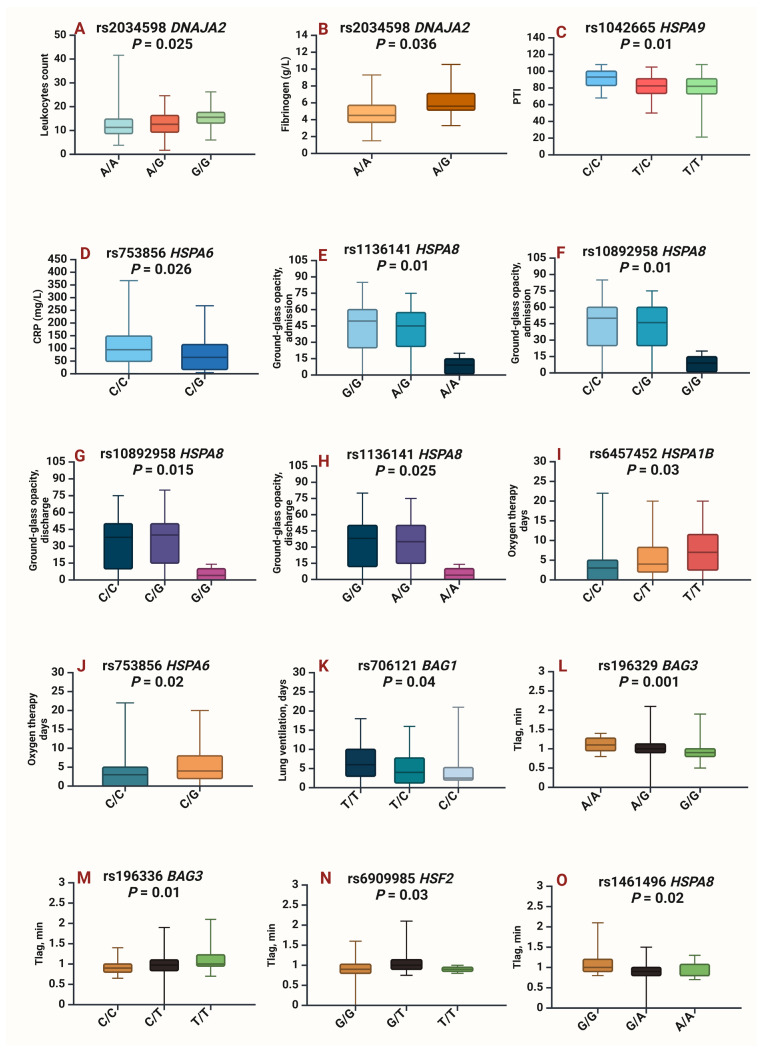
Associations of *HSPs* SNPs with the clinical/laboratory parameters of COVID-19 patients. (**A**)—Leukocyte values for rs2034598 *DNAJA2 p* = 0.025, (**B**)—fibrinogen values for rs2034598 *DNAJA2 p* = 0.036, (**C**)—prothrombin index (PTI) values for rs1042665 *HSPA9 p* = 0.01, (**D**)—C-reactive protein (CRP) values for rs753856 *HSPA6 p* = 0.026, (**E**)—ground-glass opacity on admission for rs1136141 *HSPA8 p* = 0.01, (**F**)—ground-glass opacity on admission for rs10892958 *HSPA8 p* = 0.01, (**G**)—ground-glass opacity upon discharge for rs10892958 *HSPA8 p* = 0.015, (**H**)—ground-glass opacity upon discharge for rs1136141 *HSPA8 p* = 0.025, (**I**)—oxygen therapy days for rs6457452 *HSPA1B p* = 0.03, (**J**)—oxygen therapy days for rs753856 *HSPA6 p* = 0.02, (**K**)—days on lung ventilation for rs706121 *BAG1 p* = 0.04, (**L**)—time to the start of clot growth (Tlag, minutes) for rs196329 *BAG3 p* = 0.001, (**M**)—Tlag for rs196336 *BAG3 p* = 0.01, (**N**)—Tlag for rs6909985 *HSF2 p* = 0.03, (**O**)—Tlag for rs1461496 *HSPA8 p* = 0.02. Created in BioRender.com. Karpenko, A.R.; Kobzeva, K.A.; Orlov, Y.L.; Bushueva, O.Y. (2025) https://app.biorender.com/illustrations/662a2291c7516e481b232af9 (accessed on 9 September 2025).

The findings of the associations between *HSPs* SNPs and the clinical/biochemical parameters of patients with severe COVID-19 are shown in Figure 2 and Table S4.

The SNP rs2034598 *DNAJA2* was found to be associated with a higher leukocyte count (*p* = 0.025) and an increased amount of fibrinogen (*p* = 0.036) (Figure 2A,B). The SNP rs1042665 *HSPA9* was associated with an increase in PTI (*p* = 0.009) (Figure 2C), whereas the SNP rs753856 *HSPA6* was associated with a reduction in CRP (*p* = 0.026) in heterozygotes (Figure 2D). Notably, two SNPs within the *HSPA8* gene were associated with lower ground-glass opacification in the lungs. Specifically, rs1136141 *HSPA8* (*p* = 0.007) and rs10892958 *HSPA8* (*p* = 0.007) were associated with lower ground-glass opacification upon admission to the ICU (Figure 2E,F). Moreover, these same SNPs, rs10892958 *HSPA8* (*p* = 0.015) and rs1136141 *HSPA8* (*p* = 0.025), were associated with a reduction in ground-glass opacity upon discharge from the ICU (Figure 2G,H).

Regarding the associations between *HSPs* SNPs and respiratory failure indicators, we found that rs6457452 *HSPA1B* (*p* = 0.03) and rs753856 *HSPA6* (*p* = 0.02) were linked to prolonged oxygen therapy (Figure 2I,J), whereas rs706121 *BAG1* (*p* = 0.04) decreased the duration of lug ventilation (Figure 2K), indicating a better prognosis.

Furthermore, several SNPs are associated with a parameter of thrombodynamics, known as the time to the start of clot growth (Tlag, in minutes). Specifically, an increase in Tlag was associated with rs196336 *BAG3* (*p* = 0.014), rs196329 *BAG3* (*p* = 0.001), and rs6909985 *HSF2* (*p* = 0.03) (Figure 2L–N). However, rs1461496 *HSPA8* (*p* = 0.02) reduced Tlag (Figure 2O).

### 2.4. Gene-Gene Interactions Associated with Severe COVID-19 (MB-MDR, MDR Modeling)

Using the MB-MDR approach, the six most significant gene-gene interaction patterns associated with severe COVID-19 were identified: three two-locus, one three-locus and two four-locus (*p*_perm_ ≤ 0.001) (Table 3).

In total, the best models of G×G interactions included six polymorphic loci, five of which, rs7189628 *DNAJA2*, rs2034598 *DNAJA2*, rs10892958 *HSPA8*, rs706121 *BAG1*, and rs4279640 *HSF1*, were involved in the formation of two or more of the most significant G×G interactions.

For these models (intergenic interactions), the cross-validation, sensitivity, and specificity values were calculated (Table S5). It turned out that the G×G models are characterized by high specificity; however, the sensitivity is low (the maximum sensitivity and specificity indices—57.53% and 61.15%—were achieved for the intergenic interaction model rs4279640 *HSF1* × rs7189628 *DNAJA2* × rs706121 *BAG1* × rs1136141 *HSPA8*).

**Figure 3:**
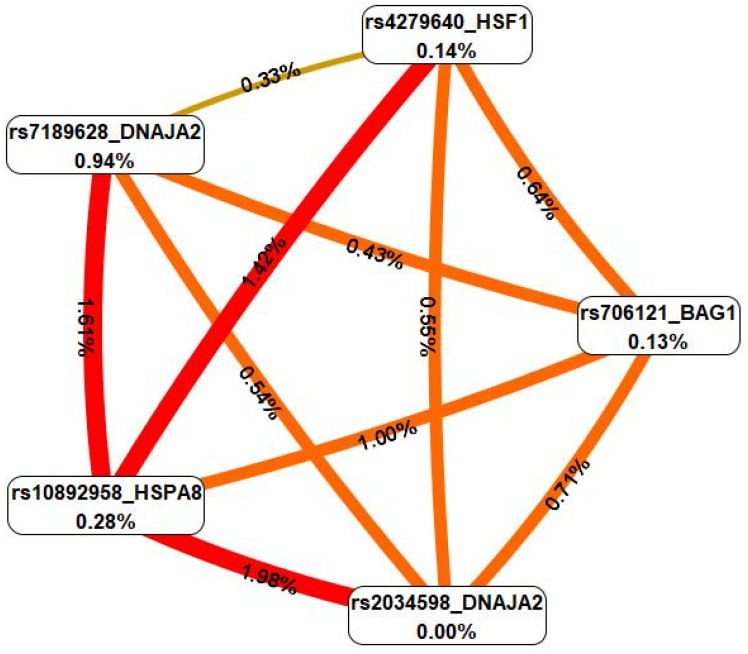
Graph reflecting the structure and power of the most significant G×G interactions of the *HSPs* loci associated with severe COVID-19. Notes: the color of the lines reflects the nature of the interaction: red and orange lines indicate strong and moderate synergism, brown lines indicate additive (independent) effects, and % reflects the strength and direction of the phenotypic effect of the gene-gene interaction (% of entropy).

In the next step, we analyzed the mechanisms of interactions between these genetic variants via the multifactor dimensionality reduction (MDR) method (Figure 3).

First, MDR revealed that the genetic variants included in the best G×G models were predominantly characterized by moderate to strong synergism, with the exception of the SNPs rs7189628 *DNAJA2* and rs4279640 *HSF1*, which exhibited additive (independent) effects (in interaction with each other). Second, rs7189628 *DNAJA2* was characterized by the highest mono-effect (0.94% contribution to the entropy of the trait) in comparison with other SNPs included in the best models of intergenic interactions (0–0.28% contribution to the entropy). Third, the effects of intergenic interactions (0.33–1.98% contribution to entropy) were comparable to the mono-effects of SNPs characterizing the best intergenic interactions, with the exception of rs2034598 *DNAJA2*, which was characterized by the absence of a mono-effect; however, its interaction with rs10892958 *HSPA8* was characterized by the maximum contribution to the entropy of the trait. Fourth, the following combinations of genotypes of polymorphic gene variants had the strongest correlations with severe COVID-19: rs7189628 *DNAJA2* C/T × rs2034598 *DNAJA2* A/G (beta = 0.22538, *p* = 0.0002); rs7189628 *DNAJA2* C/T × rs10892958 *HSPA8* C/G (beta = 0.23318, *p* = 0.0004); rs7189628 *DNAJA2* C/T × rs706121 *BAG1* T/C (beta = 0.261094, *p* = 0.0006); rs7189628 *DNAJA2* C/T × rs10892958 *HSPA8* C/G × rs2034598 *DNAJA2* A/G (beta = 0.487459, *p* = 6.216 × 10^−7^); rs4279640 *HSF1* T/T × rs7189628 *DNAJA2* C/T × rs10892958 *HSPA8* C/G × rs706121 *BAG1* T/T (beta = 0.549438, *p* = 7.265 × 10^−5^); and rs4279640 *HSF1* C/C × rs7189628 *DNAJA2* C/C × rs706121 *BAG1* T/T × rs1136141 *HSPA8* A/A (beta = 0.593388, *p* = 0.0016) (Table S6).

### 2.5. Gene-Environment Interactions Associated with Severe COVID-19 (MB-MDR, MDR Modeling)

Using the MB-MDR approach, the seven most significant gene-environment interaction patterns associated with severe COVID-19 were identified: one two-level, three three-level, and three four-level interaction patterns (Table 4).

In total, the best models of G×E interactions involved smoking in interaction with seven SNPs, four of which—rs7189628 *DNAJA2*, rs2034598 *DNAJA2*, rs196329 *BAG3*, and rs1043618 *HSPA1A*—were involved in two or more of the most significant models of G×E interactions.

For the G×E models, the cross-validation, sensitivity, and specificity indices were also calculated (Table S7). The highest specificity indices (92.97%) were observed for rs7189628 *DNAJA2* × rs4926222 *DNAJB1* × SMOKE. The highest sensitivity indices (71.45%) were observed for the model rs4279640 *HSF1* × rs7189628 *DNAJA2* × rs196329 *BAG3* × SMOKE.

**Figure 4:**
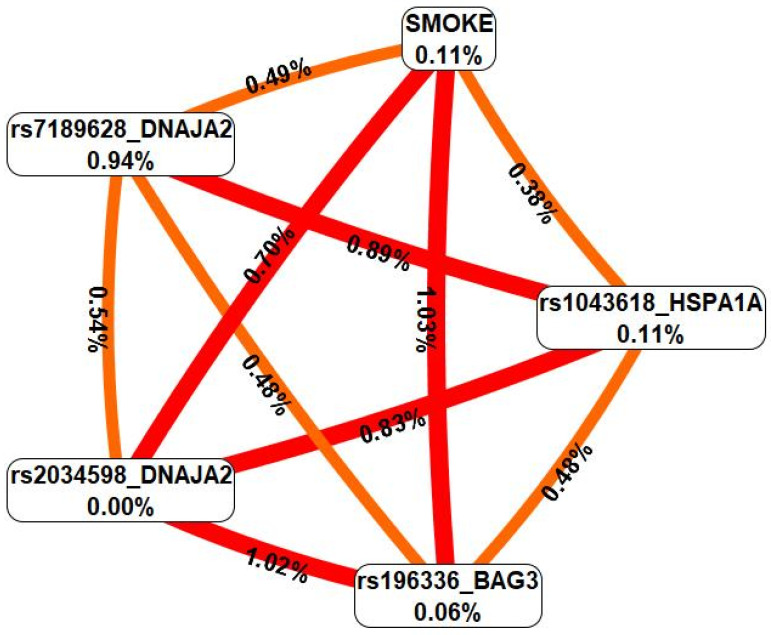
Graph reflecting the structure and power of the most significant G×E interactions of the *HSPs* loci associated with severe COVID-19. Notes: the color of the lines reflects the nature of the interaction: orange and red indicate moderate and strong synergism, respectively; and % reflects the strength and direction of the phenotypic effect of the gene-environment interaction (% of entropy).

In the next step, we analyzed the interactions between these genetic variants and smoking via the MDR method (Figure 4).

First, MDR revealed that an environmental risk factor such as smoking was characterized by moderate and pronounced synergism in interaction with all the SNPs included in the two or more most significant gene-environment interactions. Second, all the SNPs characterizing the best G×E models interacted synergistically with each other. Third, the effects of genotype-environment interactions (contribution to the entropy of a trait) were comparable to/exceeded the mono-effects of smoking/SNPs. Fourth, rs7189628 *DNAJA2* was characterized by a complete absence of a mono-effect but rather high entropy values in interaction with smoking (0.70%) and in interaction with SNPs characterizing the most significant G×E models (0.54–1.02% contribution to the entropy of the trait). Notably, all of the most significant gene-environment interaction models included the genetic variant rs7189628 *DNAJA2*. Fifth, the genes with the strongest connections with severe COVID-19 had the following gene-environmental interactions: rs7189628 *DNAJA2* C/T × smoking (beta = 0.21879, *p* = 0.0006); rs7189628 *DNAJA2* C/T × rs2034598 *DNAJA2* A/G × smoking (beta = 0.499993, *p* = 2.39 × 10^−6^); rs7189628 *DNAJA2* C/T × rs196329 *BAG3* G/G × smoking (beta = 0.28850, *p* = 0.0004); rs7189628 *DNAJA2* C/T × rs4926222 *DNAJB1* A/G × nonsmoking (beta = 0.2655907, *p* = 0.01); rs4279640 *HSF1* T/T × rs7189628 *DNAJA2* C/T × rs196329 *BAG3* G/G × smoking (beta = 0.534638, *p* = 3.307 × 10^−6^); and rs7189628 *DNAJA2* C/T × rs2034598 *DNAJA2* A/G × rs1043618 *HSPA1A* C/C × nonsmoking (beta = 0.8455910, *p* = 5.945 × 10^−5^) (Table S8).

### 2.6. Functional Annotation

#### 2.6.1. eQTL Effects

**Figure 5:**
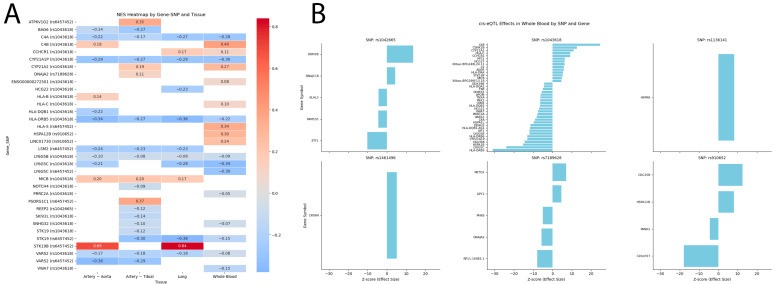
cis-eQTL effects of HSP SNPs. (**A**) Heatmap of cis-eQTL associations for risk-associated SNPs in lung, arterial tissues, and whole blood (GTEx Portal data). (**B**) Bar plots of cis-eQTL effects in whole blood (eQTLGen Browser data).

The analysis of the cis-eQTL effects of risk-associated *HSPs* SNPs revealed several noteworthy associations (Tables S9 and S10, Figure 5A,B). The A allele of rs1461496 *HSPA8* was found to increase the expression of *CRTAM* in blood, whereas the A allele of rs1136141 *HSPA8* increased the expression of *HSPA8* in blood (Figure 5A,B). Additionally, the C allele of rs1042665 *HSPA9* was associated with elevated expression of *KDM3B* and *DNAJC18* and reduced expression of *ETF1*, *FAM53C*, and *KLHL3* in blood (Figure 5A,B).

Furthermore, the C allele of rs910652 *HSPA12B* increased the expression of *HSPA12B* and *LINC01730* in the blood and tibial artery (Figure 5A,B). The T allele of rs6457452 *HSPA1B* increased the expression of *STK19B* in the aorta, tibial artery, lung, and whole blood, as well as *PSORS1C1* and *PSORS1C2* in arterial tissues; conversely, it decreased the expression of *STK19* in the lung and whole blood; *VARS2* in the aorta and tibial artery; *LSM2* in the tibial artery and lung; and *LY6G5C* in whole blood (Figure 5A,B).

Significant number of cis-eQTL effects were observed for rs1043618 *HSPA1A*: notably its allele C is associated with changes in expression of genes encoding Human Leukocyte Antigen (HLA), cytochromes, *NOTCH4*, and others (Figure 5A,B).

Finally, the allele T of rs7189628 *DNAJA2* elevated the expression of *NETO2* and *GPT2* while reducing the expression of *RP11*-*169E6.1*, *DNAJA2*, and *PHKB* in whole blood (Figure 5A,B). Additionally, allele C of rs7189628 *DNAJA2* is associated with increased expression of *DNAJA2* itself in tibial artery.

#### 2.6.2. Histone Modifications

We identified high regulatory potential for rs1461496 *HSPA8*, rs1136141 *HSPA8*, rs1043618 *HSPA1A*, and rs6457452 *HSPA1B* in both blood and aorta, along with rs1042665 *HSPA9* and rs7189628 *DNAJA2*, which are expressed specifically in the blood (Table S11). These SNPs are located in regions that bind to the histone modifications H3K4me1 and H3K4me3, and their effects are amplified by enhancer-marking H3K27ac and promoter-marking H3K9ac. Furthermore, rs1461496 *HSPA8*, rs1136141 *HSPA8*, rs1043618 *HSPA1A*, and rs6457452 *HSPA1B* are located within DNA regions hypersensitive to DNase-1 in blood samples. Additionally, in lung tissue, the studied SNPs presented at least one enhancer or promoter marker, except for rs910652 *HSPA12B* (Table S11).

#### 2.6.3. Bioinformatic Analysis of the Associations of the Studied SNPs with COVID-19 and Related Phenotypes

According to the Lung Disease Knowledge Portal bioinformatic resource, first, rs910652 *HSPA12B* was linked to a lower risk of very severe respiratory confirmed COVID-19 cases, whereas rs6457452 *HSPA1B* was associated with an increase in hospitalization due to COVID-19 (Table S12). Second, rs1136141 *HSPA8*, rs6457452 *HSPA1B*, and rs1043618 *HSPA1A* were implicated in reducing lung capacity parameters such as forced expired volume in 1 s (FEV1) and forced vital capacity (FVC) (Table S12).

#### 2.6.4. Analysis of Transcription Factors

The protective allele G rs1461496 *HSPA8* creates DNA-binding sites for 22 TFs that are jointly involved in the following overrepresented biological processes: regulation of transforming growth factor beta2 production (GO:0032909; FDR = 0.0025), positive regulation of extracellular matrix assembly (GO:1901203; FDR = 0.00681), activin receptor signaling pathway (GO:0032924; FDR = 0.021), positive regulation of epithelial to mesenchymal transition (GO:0010718; FDR = 0.00153), positive regulation of nitric oxide biosynthetic process (GO:0045429; FDR = 0.0328), regulation of transforming growth factor beta receptor signaling pathway (GO:0017015; FDR = 0.0149), cellular response to transforming growth factor beta stimulus (GO:0071560; FDR = 0.0176), SMAD protein signal transduction (GO:0060395; FDR = 0.019), and response to hypoxia (GO:0001666; FDR = 0.00637) (Table S8). In contrast, the risk allele A rs1461496 *HSPA8* creates binding sites for 32 TFs involved in epithelial tube branching involved in lung morphogenesis (GO:0060441; FDR = 0.0402), lymphocyte differentiation (GO:0030098; FDR = 0.0385), negative regulation of epithelial cell differentiation (GO:0030857; FDR = 0.00389), positive regulation by host of viral transcription (GO:0043923; FDR = 0.0209), canonical Wnt signaling pathway (GO:0060070; FDR = 0.00144) (Table S13).

The risk allele A rs1136141 *HSPA8* generates DNA-binding sites for 18 TFs that participate in hippo signaling (GO:0035329; FDR = 0.0219) and leukocyte differentiation (GO:0002521; FDR = 0.00251), whereas the protective allele G rs1136141 *HSPA8* generates DNA-binding sites for 51 TFs involved in four overrepresented biological processes: positive regulation of CD8-positive, alpha-beta T-cell differentiation (GO:0043378; FDR = 0.0043), response to cAMP (GO:0051591; FDR = 0.0452), and negative regulation of leukocyte cell–cell adhesion (GO:1903038; FDR = 0.0121) (Table S14).

The risk allele C rs1042665 *HSPA9* creates DNA-binding sites for 66 TFs involved in lymph vessel development (GO:0001945; FDR = 0.00186), positive regulation of BMP signaling pathway (GO:0030513; FDR = 0.00427), regulation of epithelial to mesenchymal transition (GO:0010717; FDR = 0.00415), and lung development (GO:0030324; FDR = 0.0352) (Table S15).

The protective allele C rs910652 *HSPA12B* generates DNA-binding sites for 21 TFs, which function in biological processes such as interleukin-9-mediated signaling pathway (GO:0038113; FDR = 0.00454), interleukin-2-mediated signaling pathway (GO:0038110; FDR = 0.00569), growth hormone receptor signaling pathway via JAK-STAT (GO:0060397; FDR = 0.00755), interleukin-15-mediated signaling pathway (GO:0035723; FDR = 0.00859), cellular response to estrogen stimulus (GO:0071391; FDR = 0.0216), positive regulation of epithelial cell proliferation (GO:0050679; FDR = 0.00004), cellular response to transforming growth factor beta stimulus (GO:0071560; FDR = 0.000426) (Table S16). Conversely, the risk allele T rs910652 *HSPA12B* creates DNA-binding sites for 39 TFs, which function in cellular response to prostaglandin E stimulus (GO:0071380; FDR = 0.027), cellular response to hypoxia (GO:0071456; FDR = 0.00344), and response to cytokine (GO:0034097; FDR = 0.0407) (Table S16).

The risk allele T rs7189628 *DNAJA2* generates binding sites for 27 TFs, participating in various overrepresented biological processes: negative regulation of interleukin-4 production (GO:0032713; FDR = 0.00301), negative regulation of interleukin-5 production (GO:0032714; FDR = 0.0036), lymphocyte proliferation (GO:0046651; FDR = 0.0227), positive regulation by host of viral transcription (GO:0043923; FDR = 0.0139), positive regulation of vascular endothelial growth factor production (GO:0010575; FDR = 0.000842), cytokine production (GO:0001816; FDR = 0.0282), B cell homeostasis (GO:0001782; FDR = 0.0291), epithelial cell apoptotic process (GO:1904019; FDR = 0.00339), regulation of transforming growth factor beta production (GO:0071634; FDR = 0.047), transforming growth factor beta receptor superfamily signaling pathway (GO:0141091; FDR = 0.0407), cellular response to hypoxia (GO:0071456; FDR = 0.0238), positive regulation of cell-cell adhesion (GO:0022409; FDR = 0.0229), response to oxidative stress (GO:0006979; FDR = 0.0324), leukocyte differentiation (GO:0002521; FDR = 0.0497), cellular response to cytokine stimulus (GO:0071345; FDR = 0.0417) (Table S17). However, the protective allele C rs7189628 *DNAJA2* also results in the loss of DNA-binding sites for 64 TFs and functions such as cellular response to reactive oxygen species (GO:0034614; FDR = 0.029), lymphocyte differentiation (GO:0030098; FDR = 0.0000708), lymphocyte activation (GO:0046649; FDR = 0.00238), and leukocyte activation (GO:0045321; FDR = 0.00958) (Table S17). Of note, no TFs were found for rs1043618 *HSPA1A*.

## 3. Discussion

Alterations in chaperone function contribute to the progression of various diseases, including neurodegeneration [25], ischemic stroke [26,27], and cancer [28,29]. Chaperones play critical roles in cellular processes by ensuring survival and resilience under diverse stress conditions, such as oxidative stress [30] and inflammation [31], both of which are key hallmarks of COVID-19 pathology [32,33].

In this study, we are the first to identify associations between HSPs SNPs and the risk of severe COVID-19. Specifically, the polymorphic variants rs1136141 and rs1461496 *HSPA8*, rs1042665 *HSPA9*, rs7189628 *DNAJA2*, rs910652 *HSPA12B*, rs6457452 *HSPA1B* and rs1043618 *HSPA1A* are associated with alerted risk of severe COVID-19. Additionally, we demonstrated how sex, smoking, dietary habits, and physical activity influence these associations. Notably, two SNPs rs1136141 *HSPA8* and rs7189628 *DNAJA2* are associated with severe COVID-19 in patients under 68 years of age, whereas in older patients, SNPs rs1461496 *HSPA8*, rs910652 *HSPA12B*, rs1043618 *HSPA1A* and rs6457452 *HSPA1B* are significantly associated with severe disease. We further explored how HSPs SNPs affect clinical and biochemical parameters, including thrombodynamic markers. Importantly, we revealed strong synergistic effects of gene-gene and gene-smoking interactions. Finally, via the use of bioinformatics tools, we performed a comprehensive functional annotation of the risk-associated HSPs SNPs, identifying their possible molecular mechanisms underlying the severe progression of COVID-19.

**Figure 6:**
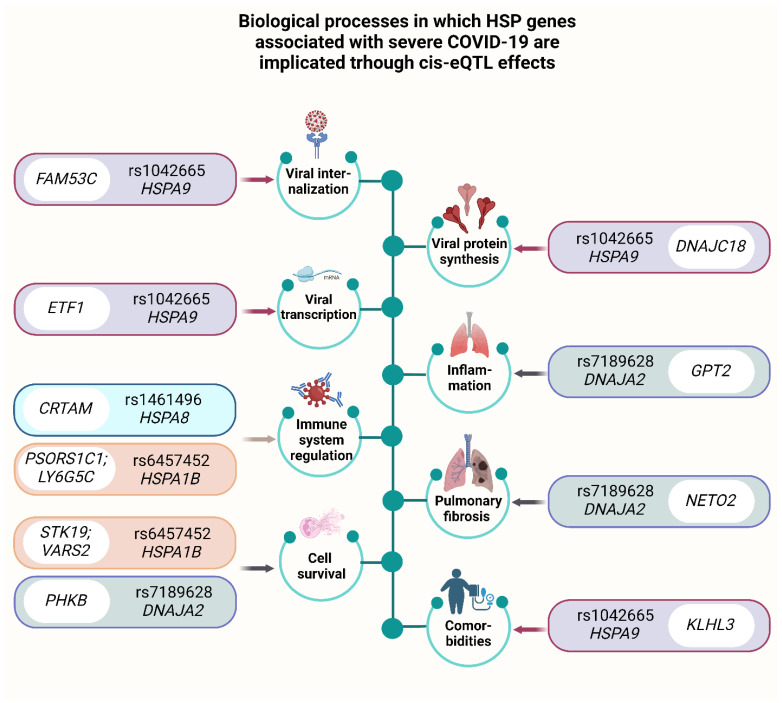
cis-eQTL effects of HSP variants associated with severe COVID-19 and their involvement in key biological processes. SNPs in HSPs were linked to expression changes in nearby genes, which in turn participate in pathways relevant to viral entry and replication, immune system regulation, inflammation, pulmonary fibrosis, and comorbidities. The schematic summarizes how genetic variation in HSP genes may contribute to COVID-19 pathogenesis through downstream cis-eQTL effects. Created in BioRender.com. Karpenko, A.R.; Kobzeva, K.A.; Orlov, Y.L.; Bushueva, O.Y. (2025) https://app.biorender.com/illustrations/6630e970fe63780139922ae6 (accessed on 9 September 2025).

In Figure 6, we provide an overview of the potential mechanisms underlying the cis-eQTL effects of HSPs SNPs in severe COVID-19.

Figure 6 shows effects of viral internalization, vial transcription, immune system regulation, cell survival, viral protein synthesis, inflammation, pulmonary fibrosis, comorbidities and associated SNPs.

**Figure 7:**
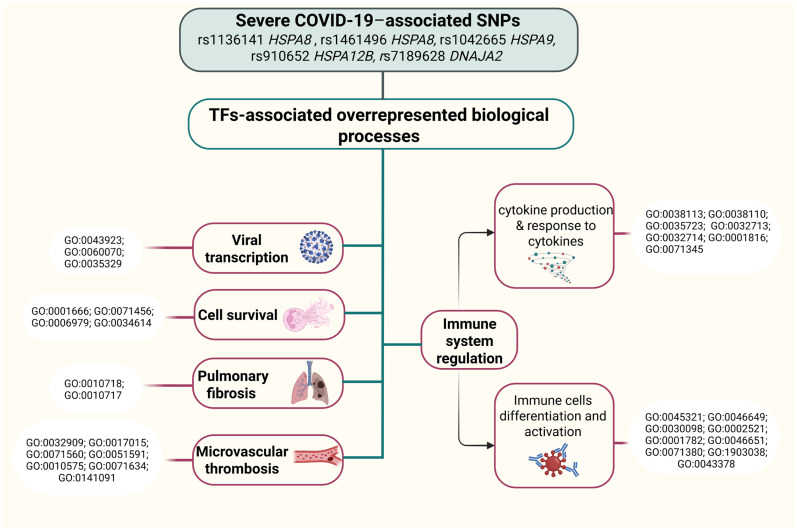
Gene ontology (GO) terms of transcription factor–associated biological processes linked to severe COVID-19. SNPs in HSPs were associated with transcription factors regulating biological processes relevant to severe disease. Overrepresented GO terms clustered into pathways involved in viral transcription, cell survival, pulmonary fibrosis, microvascular thrombosis, and immune system regulation, including cytokine production/response and immune cell differentiation and activation. Created in BioRender.com. Karpenko, A.R.; Kobzeva, K.A.; Orlov, Y.L.; Bushueva, O.Y. (2025) https://app.biorender.com/illustrations/6638921eb27497a7cfaf82eb (accessed on 9 September 2025).

Additionally, Figure 7 outlines the impacts of TF-associated overrepresented biological processes of HSPs SNPs that contribute to COVID-19 pathogenesis.

We observed that the risk allele T of rs7189628 *DNAJA2* is associated with an increased risk of severe COVID-19 across the entire group, with this risk being modulated by various factors, such as sex, age, smoking status, and dietary habits, but is not influenced by levels of physical activity.

In the context of gene-gene interactions, rs7189628 *DNAJA2* played a key role in the most significant G×G models, exhibiting the greatest mono-effect. Interestingly, in gene-environment interactions, rs7189628 in *DNAJA2* demonstrated a complete absence of a mono-effect. Instead, it displayed high entropy values in its interaction with smoking and contributed to the formation of all the most significant models of gene-smoking interactions.

*DNAJA2*, a member of the DnaJ heat shock protein family (Hsp40), plays a crucial role in regulating molecular chaperone activity and acts as a cochaperone of Hsp70s.

Our bioinformatic analysis revealed that the risk allele T of rs7189628 *DNAJA2* leads to elevated expression of *NETO2* and *GPT2* while reducing the expression of *RP11-169E6.1*, *DNAJA2*, and *PHKB* in whole blood (Figure 6).

*NETO2*, also known as Neuropilin and Tolloid-Like 2, has been linked to the induction of epithelial–mesenchymal transition (EMT) [34]. EMT is implicated in various pathophysiological conditions, including fibrosis and organ damage [35,36,37,38], and has been recognized as a potential mechanism in the pathogenesis of COVID-19 [39]. Specifically, EMT has been associated with the transformation of alveolar epithelial cells in COVID-19 patients, which may contribute to pulmonary fibrosis and adverse respiratory outcomes [40].

Furthermore, the upregulation of *GPT2* through the cis-eQTL effects of the SNP rs7189628 *DNAJA2* is noteworthy. *GPT2*, an enzyme involved in glutamine metabolism, plays a critical role in immune function [41,42,43] and has been linked to mitigating SARS-CoV-2 replication [44]. Studies have consistently shown a decrease in circulating glutamine levels in COVID-19 patients, which is associated with disease severity [45,46,47,48,49,50]. This glutamine deficiency is considered a central metabolic feature of COVID-19 and may contribute to various pathological processes, including inflammation, immune dysfunction, and multiorgan failure [51].

Moreover, altered expression of *PHKB*, driven by rs7189628 *DNAJA2*, is of interest. PHKB, the regulatory subunit of phosphorylase kinase, has been implicated in regulating cell survival and immune responses. Circ-Phkb, a derivative of the *Phkb* gene, has been shown to inhibit cell survival [52], indicating a potential role in immune regulation and response to pathological conditions such as COVID-19.

Additionally, the involvement of rs7189628 *DNAJA2* in various biological processes through transcription factor regulation further supports its role in immune responses and immune regulation (Figure 7). Notably, the risk allele T of rs7189628 *DNAJA2* has been associated with negative regulation of interleukin-4 and interleukin-5 production, positive regulation of viral transcription and cell-cell adhesion, and regulation of vascular endothelial growth factor production and is involved in lymphocyte proliferation, leukocyte differentiation, cytokine production, the cellular response to cytokine stimulus, B-cell homeostasis, the epithelial cell apoptotic process, the cellular response to hypoxia, and the response to oxidative stress. These findings provide robust evidence of its involvement in stress responses, as well as immune regulation during COVID-19.

Furthermore, our study revealed the protective effect of *HSPA12B* SNP. Specifically, the SNP rs910652 *HSPA12B* has been associated with a decreased risk of severe COVID-19 in the entire group, females and patients with normal physical activity levels.

Heat shock protein A12B (HSPA12B), a member of the HSP70 family, is expressed primarily in endothelial cells and is known to regulate the proinflammatory response of macrophages [53]. Increased expression of *HSPA12B* in endothelial cells has been shown to attenuate lipopolysaccharide (LPS)-induced adhesion molecule expression and proinflammatory cytokine production by activating the PI3K/Akt signaling pathway [54]. Inhibition of this pathway is viewed as a possible treatment for COVID-19 [55,56,57].

According to the Lung Disease Knowledge Portal, rs910652 *HSPA12B* is linked to a reduced risk of very severe respiratory confirmed COVID-19 compared with the general population. Additionally, the protective allele C of rs910652 *HSPA12B* is involved in various signaling pathways, including interleukin-2, -9, and -15-mediated signaling pathways, the growth hormone receptor signaling pathway via JAK-STAT, the cellular response to transforming growth factor beta stimulus, positive regulation of epithelial cell proliferation, and epithelial cell differentiation (Figure 7). These biological processes are crucial for immune responses, modulation, and recovery in patients with COVID-19 [58,59,60]. Furthermore, the SNP rs910652 *HSPA12B* participates in the cellular response to estrogen stimulus by binding to TFs. Estrogen hormones have been recognized as essential factors for inhibiting inflammation and the immune response in patients with COVID-19 [61], suggesting a potential link between the protective effect of this SNP in females. Moreover, the risk allele T rs910652 *HSPA12B,* through TF binding, may exacerbate COVID-19 outcomes by participating in the cellular response to prostaglandin E stimulus, promoting severe COVID-19 by impairing the immune response [62].

Here, we also found a risk effect of allele C rs1043618 *HSPA1A* in elderly patients (aged 68 years and older). Bioinformatic annotation revealed substantial regulatory effects of this SNP via cis-eQTL effects in blood, arteries, and lungs. It is noteworthy that among those genes with altered expression are genes encoding histocompatibility complex (HLA), which are crucial for antigen presentation and immune responses [63]. Furthermore, this genetic variant is responsible for regulating CYPs expression, which are responsible for drug pharmacokinetics, steroid, and vitamin pathways in COVID-19 [64].

We found that the risk allele T rs6457452 *HSPA1B* is associated with an increased risk of severe COVID-19 in patients aged 68 years or older as well as in patients with low physical activity. HSPA1B is a member of the Hsp70 family. One study revealed that HSPA1B can inhibit viral proliferation following viral infection [65]. Data from the LKP indicate that the SNP rs6457452 *HSPA1B* increases the risk of hospitalization with COVID-19 and exacerbates the severity of its course. Simultaneously, this SNP lowers peak expiratory flow and forced the vital capacity of the lungs in idiopathic pulmonary fibrosis. Additionally, rs6457452 *HSPA1B* downregulates the expression of *STK19* in the blood and lungs. A recent study revealed that *STK19*, a DNA/RNA-binding protein, is critical for DNA damage repair (DDR) and cell proliferation [66]. Slowing these processes during SARS-CoV-2 infection can lead to poor outcomes and ICU admission, especially in older individuals. Furthermore, rs6457452 *HSPA1B* downregulates the expression of *VARS2*. Depletion of *VARS2*, a mitochondrial aminoacyl-tRNA synthetase, results in the activation of the integrated stress response (ISR) and disruptions in mitochondrial fatty acid oxidation [67], thereby decreasing cell survival (Figure 6). Notably, two genes with altered expression levels, *PSORS1C1* and *LY6G5C*, are located in the major histocompatibility complex (MHC) class I (https://www.genecards.org/cgi-bin/carddisp.pl?gene=PSORS1C1&keywords=PSORS1C1, accessed on 10 March 2025) and III regions (https://www.genecards.org/cgi-bin/carddisp.pl?gene=LY6G5C&keywords=LY6G5C, accessed on 10 March 2025), respectively. Both *PSORS1C1* and *LY6G5C* are involved in immune regulation and inflammation [68,69]

Next, we established associations of *HSPA8* SNPs with severe COVID-19, as well as with clinical manifestations. HSPA8, a member of the heat shock protein 70 (HSP70) family, has been implicated in various stages of the viral life cycle, including attachment [70,71], internalization [72], and replication [73].

First, rs1461496 *HSPA8* is linked to an increased risk of severe COVID-19 in patients aged 68 and over, while it is also associated with reduced time to the start of clot growth in the entire group (Tlag, minutes). This SNP regulates the expression of *CRTAM* via cis-eQTL effects. *CRTAM* was found to be downregulated in COVID-19 patients, and this gene regulates the activation and differentiation of several T-cell subsets, including NK cells [74]. Analysis of TFs binding to the risk allele A rs1461496 *HSPA8* revealed its involvement in various pathological pathways crucial for COVID-19, such as positive regulation by host viral transcription, lymphocyte differentiation, and the canonical Wnt signaling pathway, which can result in a cytokine storm [75]. Moreover, the protective allele G rs1461496 *HSPA8* creates DNA-binding sites for TFs involved in biological processes that may have a positive impact on COVID-19. These processes include the positive regulation of nitric oxide (NO) biosynthesis, the regulation of transforming growth factor beta (TGF-β) signaling and production, and the response to hypoxia (Figure 7). Nitric oxide affects COVID-19 through four mechanisms: regulating blood flow, initiating anti-inflammatory responses, promoting anticoagulation effects, and exerting antiviral effects [76], thus indicating the protective effect of this SNP. TGF-β influences immune cell development, differentiation, tolerance induction, and homeostasis. A study revealed that serum levels of TGF-β positively correlated with improved outcomes in COVID-19 patients [77].

Second, rs1136141 *HSPA8* is associated with increased risk of severe COVID-19 in patients under 68 years of age as well as in patients with inadequate intake of fresh fruit and vegetables. Additionally, both rs1136141 and rs10892958 *HSPA8* are associated with a lower area of lung lesions upon admission to the ICU and discharge from the ICU. Data from the LKP also associate rs1136141 *HSPA8* with a reduction in forced expired volume in 1 s (FEV1), forced vital capacity (FVC), and peak expiratory flow. Moreover, the risk allele A rs1136141 *HSPA8*, through TF binding, is involved in leukocyte differentiation and hippo signaling, the inhibition of which significantly reduces SARS-CoV-2 replication [78]. Conversely, the protective allele G rs1136141 *HSPA8*, by binding of TFs, positively regulates CD8-positive, alpha-beta T-cell differentiation, negatively regulates leukocyte cell–cell adhesion, and is involved in the response to cAMP, a factor known to prevent antibody-mediated coagulopathy in patients with COVID-19 [79] (Figure 7).

Finally, in our study, we identified the SNP rs1042665 *HSPA9* as a risk allele for severe COVID-19 across groups with normal physical activity levels and adequate intake of fresh fruit and vegetables. This finding suggests that environmental risk factors such as oxidative stress from insufficient exercise or low antioxidant intake may override the genetic effect of this variant. Additionally, this SNP was associated with an increase in PTI, indicating a potential link to thrombosis.

HSPA9, a chaperone protein, plays an important role in mitochondrial iron-sulfur cluster (ISC) biogenesis [80]. Notably, viral proteins often rely on [FeS] clusters for their structural and catalytic functions. For example, the RNA-dependent RNA polymerase (RdRp) of SARS-CoV-2 contains two 4Fe-4S3(His) clusters, which support its catalytic activity and provide structural stability to the RdRp complex. It is conceivable that the host [FeS] biosynthesis machinery is also manipulated by viruses for the maturation of viral [FeS] proteins [81].

Our analysis of the cis-eQTL effects of the SNP rs1042665 *HSPA9* suggests a complex interplay of gene regulation involving *KDM3B*, *ETF1, FAM53C*, *KLHL3*, and *DNAJC18*, potentially contributing to COVID-19 severity. Upregulation of *KDM3B*, a histone H3K9 demethylase, can result in dysregulation of the cell cycle and cell proliferation [82,83], whereas downregulation of *ETF1*, a protein crucial for the termination of RNA translation [84], may impact the synthesis of viral proteins, including SARS-CoV-2 in host cells [85]. Moreover, FAM53C, which was recently found to be a suppressive binding partner of DYRK1A [86], regulates *ACE2* and *DPP4* transcription to support SARS-CoV-2 entry [87]. *KLHL3* can increase the severity of COVID-19 through its effects on metabolism and the development of comorbidities such as hypertension and obesity [88,89]. Furthermore, *DNAJC18* was found to be involved in virus endoplasmic reticulum membrane penetration and infection [90], suggesting a potential role in COVID-19 pathogenesis.

Moreover, our analysis of TFs revealed that the risk allele C rs1042665 *HSPA9* is involved in lymph vessel development, the regulation of epithelial-to-mesenchymal transition, and the positive regulation of the BMP signaling pathway, which may increase the susceptibility of lung cells to SARS-CoV-2 infection [91]. These findings shed light on how this SNP could exacerbate the course of COVID-19, potentially leading to fibrosis and worsened outcomes.

## 4. Materials and Methods

**Figure 8:**
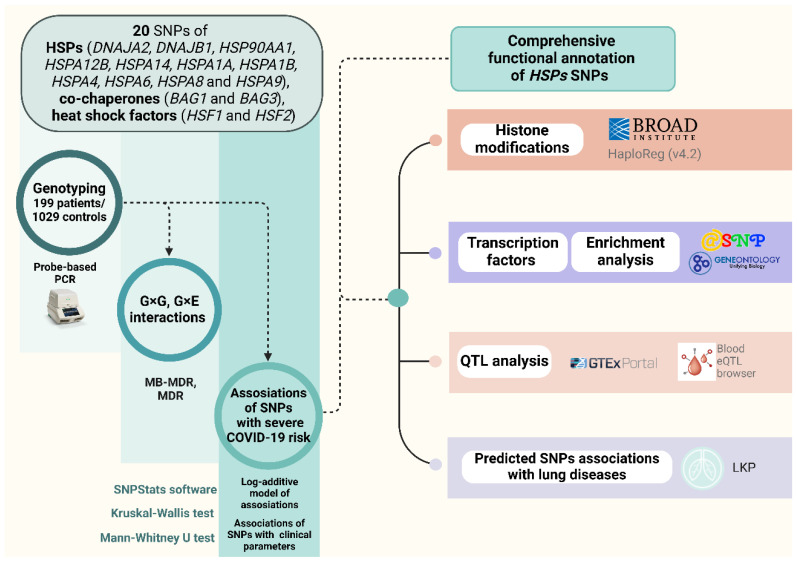
Design of the study. Created in BioRender.com. Karpenko, A.R.; Kobzeva, K.A.; Orlov, Y.L.; Bushueva, O.Y. (2025) https://app.biorender.com/illustrations/676153ed85a9d9e3d6c5f302 (accessed on 9 September 2025).

Figure 8 presents the data, methods, and study flow.

### 4.1. Study Participants

The study included 199 hospitalized COVID-19 patients and 962 healthy controls from Central Russia. The Ethical Review Committee of Kursk State Medical University approved the study protocol (protocol No. 1 from 11 January 2022), and all participants provided written informed consent. A detailed characterization of the study participants was provided in our previous research [22,92,93]. The inclusion criteria required participants to have self-declared Russian ancestry and to be born in Central Russia. Table S18 provides the baseline and clinical characteristics of the study cohort.

Patients were enrolled in the study during the COVID-19 pandemic from 2020–2022 at the intensive care units (ICUs) of Kursk Regional Hospital No. 6 and the Kursk Regional Tuberculosis Dispensary. Case status was defined by a positive RT-PCR test for SARS-CoV-2 and a clinical course requiring hospitalization with advanced respiratory support.

The control group consisted of healthy volunteers from the biobank who had mild or asymptomatic COVID-19 and did not need ICU admission. Additionally, healthy patients presented no clinical symptoms of cardiovascular, cerebrovascular, or other significant illnesses. This group was recruited from the same population and during the same time period [94,95].

Smoking status, fruit and vegetable consumption, and physical activity levels were self-reported. Participants were categorized as ‘active smokers’ if they reported current regular tobacco use at the time of enrollment or diagnosis. All others were classified as non-smokers.

In accordance with WHO guidelines, low fruit and vegetable consumption was defined as consuming less than 400 g per day [96]. Adequate consumption of fresh vegetables and fruits was defined as consuming 400 g or more, equivalent to 3–4 servings per day, excluding starchy tubers such as potatoes.

Insufficient physical activity was characterized by engaging in less than 180 min per week of moderate to vigorous physical activity [97]. This encompassed various forms of exercise, including leisure activities such as walking and running, as well as fitness club exercises such as treadmill running, aerobics, or resistance training.

### 4.2. Genetic Analysis

The Laboratory of Genomic Research at the Research Institute for Genetic and Molecular Epidemiology of Kursk State Medical University (Kursk, Russia) performed genotyping. Up to 5 mL of venous blood from each participant was collected from a cubital vein, put into EDTA-coated tubes, and stored at −20 °C until processing. Defrosted blood samples were used to extract genomic DNA via the standard methods of phenol/chloroform extraction and ethanol precipitation. The purity, quality, and concentration of the extracted DNA samples were assessed via a NanoDrop spectrophotometer (Thermo Fisher Scientific, Waltham, MA, USA).

SNPs were selected based on biological relevance, functional potential, and technical feasibility. First, we focused on genes encoding proteins from the HSP40, HSP70, HSP90 and HSF families. Second, within these families we applied the following filters: MAF of at least 0.05 in the European population, high predicted functional potential by bioinformatic tools, and methodological considerations (SNPs were excluded if they posed significant complications for primer and probe design, such as extreme CG content, proximity to other polymorphic sites, or location in repetitive genomic regions).

Genotyping of the SNPs was performed via allele-specific probe-based polymerase chain reaction (PCR) according to protocols designed at the Laboratory of Genomic Research at the Research Institute for Genetic and Molecular Epidemiology of Kursk State Medical University. Primer3 software (https://primer3.ut.ee/, accessed on 15 February 2025) was used for primer design [98]. Primer and probe sequences are listed in Table S19 (see also Supplementary Materials for prior publication [99,100,101]). A real-time PCR procedure was performed in a 25-µL reaction mixture containing 1.5 units of Hot Start Taq DNA polymerase (Biolabmix, Novosibirsk, Russia), approximately 10 ng of DNA and the following concentrations of reagents: 0.25 μM of each primer; 0.1 μM of each probe; 250 μM of each dNTP; 2 mM MgCl_2_ for rs706121, rs6457452, and 2.5 mM MgCl_2_ for rs1043618, rs1042665, rs7155973, rs13161158, rs862832, and rs753856; 3 mM MgCl_2_ for rs4926222, rs17155992, rs196336, rs196329, rs1461496, and rs113645141; rs910652; 3.5 mM MgCl_2_ for rs2034598; 4.5 mM MgCl_2_ for rs10892958; 5 mM MgCl_2_ for rs6909985; 5.5 mM MgCl_2_ for rs7189628 and rs4279640; and 1xPCR buffer (67 mM Tris-HCl, pH 8.8; 16.6 mM (NH_4_)_2_SO_4_; and 0.01% Tween). The PCR procedure involved initial denaturation for 10 min at 95 °C, followed by 39 cycles at 92 °C for 30 s and 51 °C (rs1042665 and rs1461496), 52 °C (rs1461496), 58 °C (rs753856 and rs17155992), 59 °C (rs196329), 60 °C (rs6909985 and rs196336), 61 °C (rs706121 and rs2034598), 61.5 °C (rs1136141), 62 °C (rs910652), 62.5 °C (rs1043618 and rs13161158), 64 °C (rs7189628), 65 °C (rs13161158, rs7189628, rs4279640, rs6457452, rs862832, rs4926222, and rs10892958) for 1 min. Ten percent of the DNA samples were genotyped twice and blinded to the case-control status to ensure quality control. Over 99% of the data were concordant. Examples of genotype cluster plots are available in Table S1.

### 4.3. Thrombodynamics Analysis

The thrombodynamics test in platelet-free plasma was performed via the laboratory diagnostic system “Thrombodynamics Recorder TD-2”. The procedure was described in detail previously [92]. The quantitative parameters of the spatial dynamics of fibrin clot growth and spontaneous thrombus formation included time to the start of clot growth (Tlag), initial (Vi) and stationary (Vst) spatial clot growth rates (the slopes of the clot size curve vs. time for the segments of 2–6 min and 15–25 min from the start of clot growth for Vi and V, respectively), the clot size at 30 min after coagulation activation (CS), the maximum optical density of the formed clot (D), characterizing its quality, and the time of appearance of spontaneous clots in the sample (Tsp). This latter characteristic has substantial clinical value because spontaneous clots (i.e., those that do not grow from the activator surface) may be observed only in cases of severe hypercoagulable states.

### 4.4. Statistical and Bioinformatic Analysis

A post hoc power analysis was conducted to determine the statistical power of the study to detect genetic associations given our sample size (199 cases, 962 controls) and a significance threshold of α = 0.05. Power was calculated for a range of effect sizes (odds ratios [OR] from 1.2 to 2.5) and minor allele frequencies (MAFs from 0.05 to 0.4) using the pwr.2p2n.test function in R (version 3.6.3, R Foundation for Statistical Computing, Vienna, Austria), which is based on the test for two proportions. The analysis estimates the probability that a true genetic association of a specified effect size would be detected as statistically significant under the stated conditions. The results were visualized as a heat-map with ggplot2 library.

We used PLINK v1.9.0-b.7.7 (www.cog-genomics.org/plink/1.9/, accessed on 22 March 2025) for the statistical analysis, including quality control, Hardy–Weinberg equilibrium (HWE), genotype frequencies assessment, and association testing between genotypes and severe COVID-19 risk. To mitigate false-positive associations, correction for multiple comparisons was performed using the adaptive permutation test (*p*_perm_). This approach allows us to estimate significance non-parametrically, thereby safeguarding against inflated type I error rates [102]. The statistically significant level was taken as *p*_perm_ < 0.05.

In the analysis of genotype association, the log-additive model was employed. Covariates, which comprised factors indicating variations in the overall biological characteristics of the studied groups (age, Table S18), were taken into account when adjusting associations in the entire group of patients and controls. For analyses involving environmental or clinical variables with missing data, a complete-case analysis was employed. We opted for this approach over imputation to avoid introducing potential biases, as the pattern of missingness was not random and large portions of data were absent for the control group.

Gender and environmental factors are known to influence disease risk, either by lowering or increasing susceptibility [103,104,105]. Accordingly, we investigated the associations of *HSPs* SNPs in subgroups stratified by sex, smoking status, levels of fresh fruit and vegetable intake, and physical activity. In subgroup analyses based on environmental risk factors, in cases where information on the environmental risk factor in the control group was missing (for physical activity and fruit/vegetable intake), associations were analyzed on the basis of the presence or absence of the risk factor in the patient group compared with the overall control group. The Bonferroni adjustment was used in these cases (*p*-value multiplied by two).

STATISTICA software (v13.3, TIBCO Software Inc., Palo Alto, CA, USA) was used for statistical processing. The normality of the distribution of the quantitative data was assessed via the Shapiro-Wilk test. Given that the majority of the quantitative parameters exhibited deviations from a normal distribution, they are presented as the median (Me) along with the first and third quartiles [Q1 and Q3]. The Kruskal–Wallis test was used to compare quantitative variables among three independent groups. The groups were subsequently compared pairwise via the Mann–Whitney test. To compare quantitative variables among two independent groups, the Mann-Whitney test was also performed. For categorical variables, differences in statistical significance were evaluated via Pearson’s chi-square test with Yates’s correction for continuity.

Model-based multifactor dimensionality reduction (MB-MDR) analysis was used to test two-, three-, and four-level genotype combinations (G×G) and genotype-environment combinations (G×E). For each model, the empirical *p* value (*p*_perm_) was estimated via a permutation test. Models with *p*_perm_ < 0.001 were considered statistically significant. All calculations were adjusted for age. Statistical analysis was carried out via the R software environment. Models (on average, 3-4 models of each level) with the highest Wald statistics and the lowest *p*-level of significance were included in the study. Additionally, via the MB-MDR method, individual combinations of genotypes associated with the studied phenotypes were established (*p* < 0.001). Calculations were performed in the MB-MDR program for the R software environment (Version 3.6.3).

Additionally, the most significant G×G and G×E models were analyzed via the MDR method. The analysis was implemented in the MDR program (v.3.0.2) (http://sourceforge.net/projects/mdr, accessed on 15 February 2025). The MDR method was used to assess the mechanisms of interactions (synergy, antagonism, additive interactions (independent effects)) and the strength of interactions (the contribution of individual genes/environmental factors to the entropy of a trait and the contribution of interactions). The results of the MDR analysis were visualized as a graph.

The cross-validation values of the most significant models associated with the development of severe COVID-19 were calculated using the Generalized MDR (GMDR) method [106] implemented in the GMDR software (software Beta 0.9). The indices of consistency (CVC), prediction accuracy (Testing Balanced Accuracy), sensitivity (Se) and specificity (Sp) of the models were calculated taking into account adjustment for covariates.

The following bioinformatics resources were used to analyze the functional effects of *HSPs* SNPs (the approaches used are described in detail in our previous studies [99,107]):

- GTExportal (http://www.gtexportal.org/, accessed on 15 February 2025) was used to analyze the expression of quantitative trait loci (eQTLs) in blood, vessels, and lungs [108].
- eQTLGen (https://www.eqtlgen.org/, accessed on 15 February 2025) was employed for examination of *HSPs* SNPs that bind to eQTLs in peripheral blood [109]. To illustrate cis-eQTL associations, we used custom scripts in Python 3.12 (pandas, seaborn, matplotlib). Heatmaps were generated to display NES values across SNP-gene-tissue combinations, with color scale centered at zero to indicate direction of effect. For eQTLGen results, Z-scores of gene expression associations in whole blood were visualized using faceted bar plots, with genes sorted by effect size within each SNP for readability. Full association tables are provided in the Tables S9 and S10.
- HaploReg (v4.2) (https://pubs.broadinstitute.org/mammals/haploreg/haploreg.php, accessed on 15 February 2025) was utilized to assess the associations between *HSPs* SNPs and specific histone modifications (acetylation of lysine residues at positions 27 and 9 of the histone H3 protein (H3K27ac, H3K9ac), monomethylation at position 4 (H3K4me1) and trimethylation at position 4 (H3K4me3) of the histone H3 protein). Additionally, the tool was applied to investigate the positioning of SNPs in DNase-1 hypersensitive regions [110].
- The atSNP function prediction online tool (http://atsnp.biostat.wisc.edu/search, accessed on 15 February 2025) was used to evaluate the impact of *HSPs* SNPs on gene affinity to transcription factors (TFs) [111].
- Gene Ontology (http://geneontology.org/, accessed on 15 February 2025) was employed to analyze the joint involvement of TFs linked to the reference/SNP alleles in overrepresented biological processes directly related to the pathogenesis of COVID-19 [112].
- The Lung Disease Knowledge Portal (LKP) (https://cd.hugeamp.org/, accessed on 15 February 2025) was used for bioinformatic analyses of the associations of SNPs with lung diseases, intermediate phenotypes, and risk factors for COVID-19 (such as FEV1 and the FEV1-to-FVC ratio).

## 5. Conclusions

In conclusion, this study is the first to explore the associations between *HSPs* SNPs and the risk of severe COVID-19. Our findings highlight the significant associations of HSPs SNPs with patient-specific factors, including sex, smoking status, age, physical activity, and dietary intake of fresh fruits and vegetables. We identified key gene-gene and gene-environment interactions, providing valuable insights into the complex mechanisms by which HSPs SNPs contribute to severe COVID-19. Additionally, we demonstrated associations between HSPs SNPs and clinical parameters, such as thrombodynamics, the duration of oxygen therapy, lung ventilation, and ground-glass opacity in lung imaging. Finally, functional annotation of the risk-associated HSPs SNPs enabled us to propose their molecular mechanisms in the progression and severity of COVID-19.

### Study Limitations

Several limitations of our study should be noted. First, the relatively small sample size limits statistical power and the analysis therefore should be regarded as exploratory. Second, while cases and controls were matched on sex and smoking status and age was included as a covariate, information on other confounders such as BMI, comorbidities, vaccination status, and circulating SARS-CoV-2 variants was unavailable, limiting the extent of covariate adjustment. Finally, replications in larger, multi-ethnic cohorts will be essential to confirm our preliminary associations.

## Figures and Tables

**Table 1:** Results of the analysis of associations between *HSPs* SNPs and severe COVID-19 risk in the entire group.

Genetic Variant	Effect Allele	Other Allele	N	OR [95% CI] ^1^	*p* ^2^	*p*_perm_^3^
rs753856 *HSPA6*	G	C	1146	1.13 [0.78–1.64]	0.51	0.64
rs13161158 *HSPA4*	C	T	1159	1.14 [0.71–1.82]	0.60	0.55
rs1042665 *HSPA9*	C	T	1153	1.33 [1.01–1.75]	0.04	0.06
rs1043618 *HSPA1A*	C	G	1157	1.07 [0.84–1.36]	0.59	0.75
rs6457452 *HSPA1B*	T	C	1143	1.27 [0.90–1.80]	0.18	0.16
rs6909985 *HSF2*	T	G	1153	1.15 [0.83–1.59]	0.41	0.46
rs4279640 *HSF1*	C	T	1145	0.93 [0.73–1.18]	0.54	0.70
rs706121 *BAG1*	C	T	1151	1.17 [0.87–1.59]	0.30	0.28
rs17155992 *HSPA14*	A	G	1144	1.24 [0.81-1.91]	0.32	0.33
rs196336 *BAG3*	T	C	1155	1.01 [0.80–1.28]	0.94	1.00
rs196329 *BAG3*	A	G	1155	0.92 [0.70–1.21]	0.56	0.75
rs1461496 *HSPA8*	A	G	1156	1.11 [0.87–1.41]	0.42	0.42
rs10892958 *HSPA8*	G	C	1154	1.03 [0.76–1.38]	0.87	0.86
rs1136141 *HSPA8*	A	G	1108	1.25 [0.91–1.72]	0.17	0.27
rs7155973 *HSP90AA1*	A	G	1149	0.96 [0.59–1.57]	0.88	1.00
rs2034598 *DNAJA2*	G	A	1155	0.94 [0.72–1.22]	0.64	0.75
rs7189628 *DNAJA2*	T	C	1138	**2.02 [1.26–3.24]**	**0.003**	**0.002**
rs4926222 *DNAJB1*	G	A	1156	0.96 [0.68–1.35]	0.81	0.86
rs862832 *HSPA12B*	T	C	1148	0.82 [0.57–1.18]	0.28	0.25
rs910652 *HSPA12B*	C	T	1149	**0.70 [0.53–0.92]**	**0.01**	**0.01**

All calculations were performed relative to the minor alleles (Effect allele) with adjustment for age; ^1^—odds ratio and 95% confidence interval; ^2^—*p*-value; ^3^—*p*-value after permutation testing; statistically significant differences are marked in bold.

**Table 2:** Summary of the results of the analysis of associations between *HSPs* SNPs and severe COVID-19 risk in groups stratified by sex, smoking status, physical activity level, fresh fruit/vegetable intake, and age.

Genetic Variant	Effect Allele	Other Allele	N	OR [95% CI] ^1^	*p* ^2^ (*p*_bonf_)	*p*_perm_ ^3^ (*p*_bonf_)	N	OR [95% CI] ^1^	*p* ^2^ (*p*_bonf_)	*p*_perm_ ^3^ (*p*_bonf_)
			Males				Females			
rs910652 *HSPA12B*	C	T	481	0.91 [0.63–1.31]	0.60	0.45	668	**0.68 [0.47–0.98]**	**0.04**	**0.04**
rs7189628 *DNAJA2*	T	C	473	**3.53 [1.9–6.56]**	**6.8 × 10^−5^**	**7.6 × 10^−5^**	665	1.56 [0.84–2.9]	0.16	0.15
			Smokers				Nonsmokers			
rs7189628 *DNAJA2*	T	C	307	**3.99 [1.92–8.29]**	**0.0002**	**0.0003**	811	1.58 [0.9–2.78]	0.11	0.14
			Low physical activity level				Normal physical activity level			
rs910652 *HSPA12B*	C	T	1061	0.96 [0.69–1.33]	0.79 (1.0)	0.75 (1.0)	1047	**0.58 [0.39–0.88]**	**0.009** **(0.02)**	**0.007** **(0.01)**
rs6457452 *HSPA1B*	T	C	1058	**1.6 [1.08–2.37]**	**0.02** **(0.04)**	**0.02** **(0.04)**	1045	0.69 [0.38–1.25]	0.22 (0.44)	0.25 (0.5)
rs1042665 *HSPA9*	C	T	1067	1.17 [0.83–1.66]	0.36 (0.72)	0.39 (0.78)	1050	**1.47 [1.03–2.1]**	**0.03** (0.06)	**0.02** **(0.04)**
rs7189628 *DNAJA2*	T	C	1050	**1.88 [1.05–3.34]**	**0.03** (0.06)	**0.02** **(0.04)**	1037	**2.71 [1.52–4.84]**	**0.0007** **(0.001)**	**0.0009** **(0.002)**
			Low fruit and vegetable intake				Normal fruit and vegetable intake			
rs1136141 *HSPA8*	A	G	1039	**1.69 [1.2–2.36]**	**0.002** **(0.004)**	**0.002** **(0.004)**	987	0.6 [0.32–1.12]	0.11 (0.22)	0.14 (0.28)
rs1042665 *HSPA9*	C	T	1083	1.12 [0.81–1.55]	0.50 (1.0)	0.52 (1.0)	1034	**1.67 [1.14–2.46]**	**0.009** **(0.02)**	**0.009** **(0.02)**
rs7189628 *DNAJA2*	T	C	1068	**2.39 [1.45–3.95]**	**0.0007** **(0.001)**	**0.0008** **(0.002)**	1019	1.92 [0.94–3.91]	0.07 (0.14)	0.05 (0.1)
			Age < 68				Age ≥ 68			
rs1461496 *HSPA8*	A	G	952	0.91 [0.66–1.26]	0.57	0.56	204	**1.59 [1.05–2.4]**	**0.03**	**0.03**
rs1136141 *HSPA8*	A	G	914	**1.55 [1.06–2.28]**	**0.02**	**0.02**	194	0.9 [0.5–1.61]	0.71	0.75
rs1043618 *HSPA1A*	C	G	954	0.89 [0.64–1.23]	0.48	0.35	203	**1.56 [1.04–2.35]**	**0.03**	**0.03**
rs6457452 *HSPA1B*	T	C	946	0.96 [0.59–1.55]	0.85	0.86	197	**2.29 [1.16–4.54]**	**0.02**	**0.01**
rs7189628 *DNAJA2*	T	C	939	**2.02 [1.08–3.75]**	**0.03**	**0.02**	199	2.04 [0.96–4.36]	0.06	0.09

All calculations were performed relative to the minor alleles (Effect allele); ^1^—odds ratio and 95% confidence interval; ^2^—*p*-value; ^3^—*p*-value after permutation testing; *p*_bonf—_*p*-value after adjusting for multiple comparison where applicable; statistically significant differences are marked in bold.

**Table 3:** Gene-gene interactions associated with severe COVID-19 (MB-MDR modeling).

Gene-Gene Interaction Models	NH	Beta H	WH	NL	Beta L	WL	Wmax	*p*_perm_
The best two-locus models of intergenic interactions (for G×G models with *p*_min._ < 5 × 10^−5^, 1000 permutations)								
**rs7189628 *DNAJA2*** × **rs2034598 *DNAJA2***	2	0.2570	19.42	0	NA	NA	19.42	<0.001
**rs7189628 *DNAJA2*** × **rs10892958 *HSPA8***	2	0.2718	18.06	0	NA	NA	18.06	0.001
**rs7189628 *DNAJA2*** × **rs706121 *BAG1***	3	0.3072	17.94	1	−0.04071	3.210	17.94	0.001
The best three-locus models of intergenic interactions (for G×G models with *p*_min._ < 1 × 10^−8^, 1000 permutations)								
**rs7189628 *DNAJA2*** × **rs10892958 *HSPA8*** × **rs2034598 *DNAJA2***	2	0.5336	34.64	1	−0.16146	3.275	34.64	<0.001
The best four-locus models of gene-gene interactions (for G×G models with *p*_min._ < 1 × 10^−6^, 1000 permutations)								
**rs4279640 *HSF1*** × **rs7189628 *DNAJA2*** × **rs10892958 *HSPA8*** × **rs706121 *BAG1***	8	0.6104	60.27	1	−0.16122	3.061	60.27	<0.001
**rs4279640 *HSF1*** × **rs7189628 *DNAJA2*** × **rs706121 *BAG1*** × rs1136141 *HSPA8*	9	0.6407	60.23	0	NA	NA	60.23	<0.001

Note: NH is the number of interacting high-risk genotypes, beta H—regression coefficient for high-risk interactions identified at the 2nd stage of analysis, WH—Wald statistics for high-risk interactions, NL—number of interacting low-risk genotypes, beta L—regression coefficient for low-risk interactions identified at the 2nd stage of analysis, WL—Wald statistics for low-risk interactions, *p*_perm_—permutational significance levels for models (all models are adjusted for age); NA—means the absence of calculations for the beta L and WL indicators due to the absence of statistically significant low-risk interactions. Loci included in 2 or more best G×G models are indicated in bold.

**Table 4:** Gene-environment interactions associated with severe COVID-19 (MB-MDR modeling).

Gene-Environmental Interaction Models	NH	Beta H	WH	NL	Beta L	WL	Wmax	*p*_perm_
The best two-order models of gene-interactions (for G×E models with *p*_min._ < 0.001, 1000 permutations)								
**rs7189628 *DNAJA2*** × SMOKE	3	0.2551	17.09	0	NA	NA	17.09	<0.001
The best three-order models of gene-interactions (for G×E models with *p*_min._ < 1 × 10^−7^, 1000 permutations)								
**rs7189628 *DNAJA2*** × **rs2034598 *DNAJA2*** × SMOKE	3	0.5505	32.01	0	NA	NA	32.01	<0.001
**rs7189628 *DNAJA2*** × **rs196329 *BAG3*** × SMOKE	4	0.3641	29.19	0	NA	NA	29.19	<0.001
**rs7189628 *DNAJA2*** × rs4926222 *DNAJB1* × SMOKE	6	0.2854	28.81	0	NA	NA	28.81	<0.001
The best four-order models of gene-interactions (for G×E models with *p*_min._ < 1 × 10^−12^, 1000 permutations)								
rs4279640 *HSF1* × **rs7189628 *DNAJA2*** × **rs196329 *BAG3*** × SMOKE	5	0.6674	59.44	0	NA	NA	59.44	<0.001
**rs7189628 *DNAJA2*** × **rs2034598 *DNAJA2*** × **rs1043618 *HSPA1A*** × SMOKE	5	0.6554	53.12	0	NA	NA	53.12	<0.001
**rs7189628 *DNAJA2*** × rs1042665 *HSPA9* × **rs1043618 *HSPA1A*** × SMOKE	8	0.4561	52.91	0	NA	NA	52.91	<0.001

Note: NH is the number of high-risk interactions, beta H—regression coefficient for high-risk interactions identified at the 2nd stage of analysis, WH—Wald statistics for high-risk interactions, NL—number of interacting low-risk interactions, beta L—regression coefficient for low-risk interactions identified at the 2nd stage of analysis, WL—Wald statistics for low-risk interactions, *p*_perm_—permutational significance levels for models (all models are adjusted for age); Loci included in 2 or more best G×E models are indicated in bold; NA—means the absence of calculations for the beta L and WL indicators due to the absence of statistically significant low-risk interactions. Loci included in 2 or more best G×E models are indicated in bold.

## Data Availability

All data supporting the findings of this study are available within the paper and its Supplementary Materials.

## Supplementary Materials

The following supporting information can be downloaded at: https://www.mdpi.com/article/10.3390/ijms26188967/s1.
