# Supplementary material for: Genes Encoding Heat Shock Proteins Are Associated with Risk and Clinical Course of Severe COVID-19: A Pilot Study
Source: Int J Mol Sci. 2025 Sep 15;26(18):8967. doi: 10.3390/ijms26188967 (PMC12469910; doi:10.3390/ijms26188967)
Supplement: Supplementary file 1 [file ijms-26-08967-s001.zip › ijms-3849548-supplementary-final.pdf]

Table S1: Quality control metrics for the genotyped HSPs SNPs.

SNP	Missing genotypes	Observed genotypes	Missing genotype rate	Call rate	Cluster plot
rs753856 *HSPA6*	15	1161	0.013	0.987	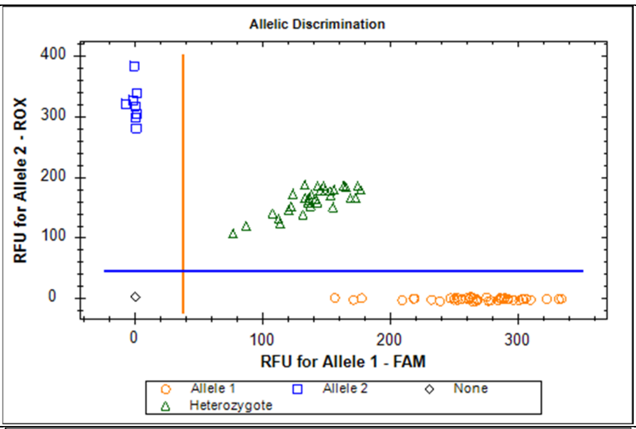
rs13161158 *HSPA4*	2	1161	0.002	0.998	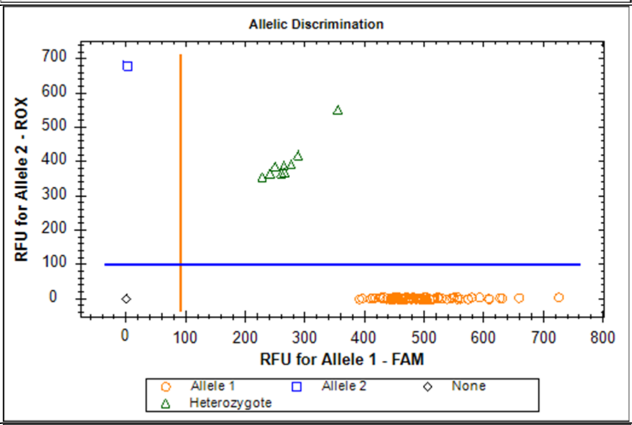
rs1042665 *HSPA9*	8	1161	0.007	0.993	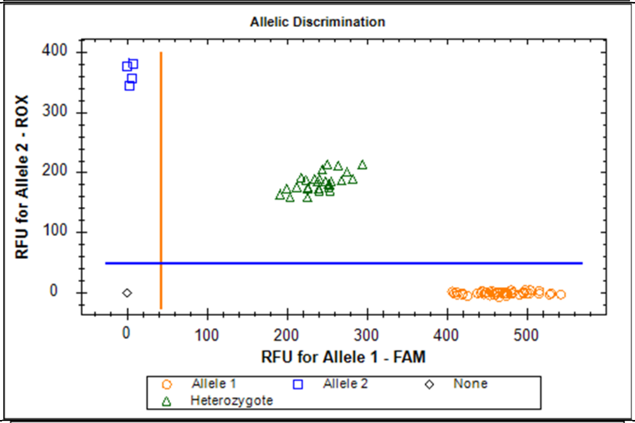
rs1043618 *HSPA1A*	4	1161	0.003	0.997	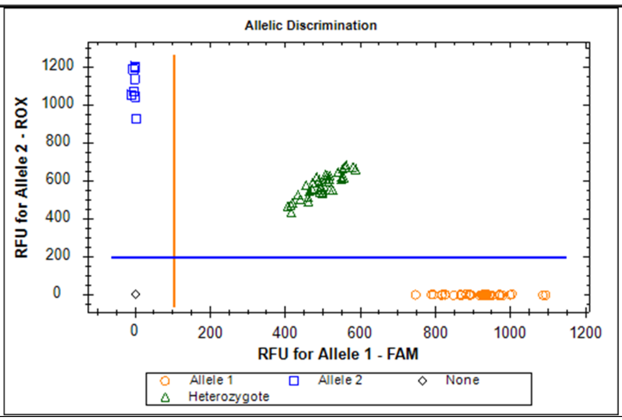

rs6457452 *HSPA1B*	18	1161	0.016	0.985	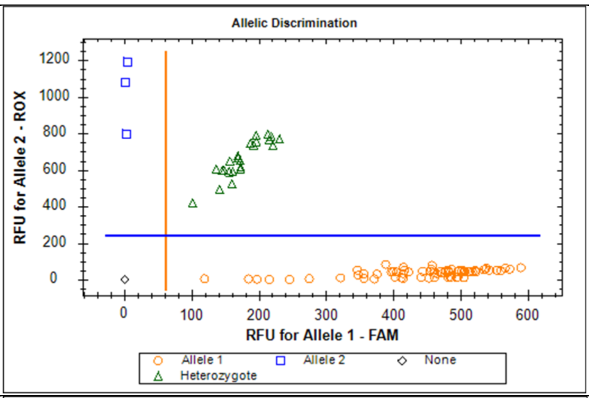
rs6909985 *HSF2*	8	1161	0.007	0.993	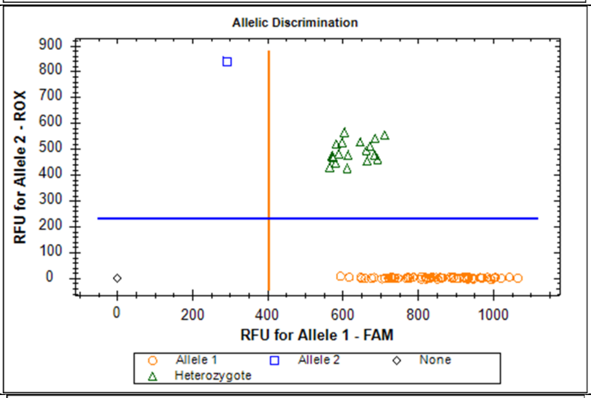
rs4279640 *HSF1*	16	1161	0.014	0.986	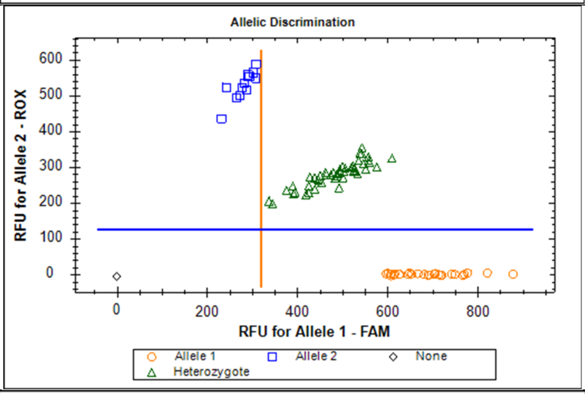
rs706121 *BAG1*	10	1161	0.009	0.991	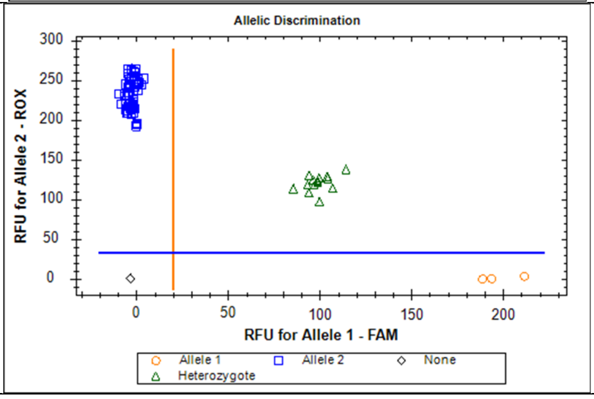

rs17155992<br>*HSPA14*	17	1161	0.015	0.985	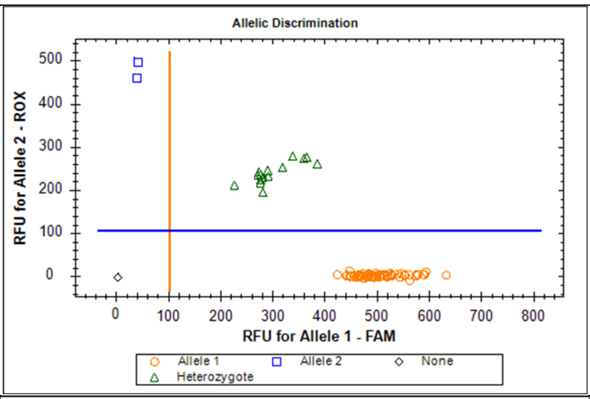
rs196336<br>*BAG3*	6	1161	0.005	0.995	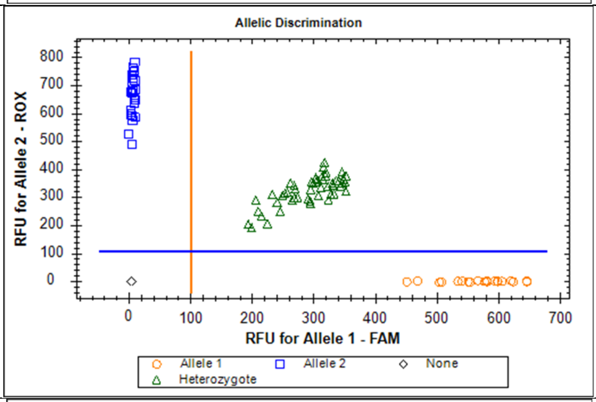
rs196329<br>*BAG3*	6	1161	0.005	0.995	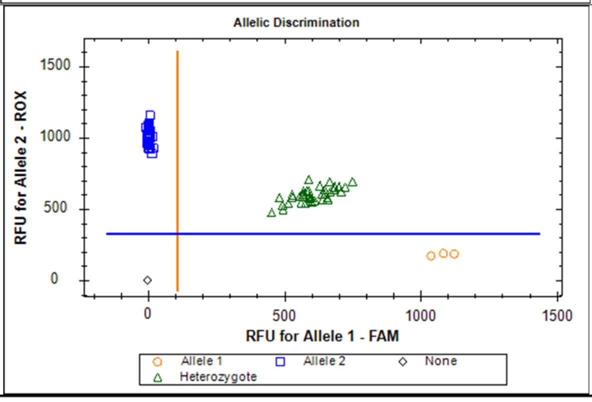
rs1461496<br>*HSPA8*	5	1161	0.004	0.996	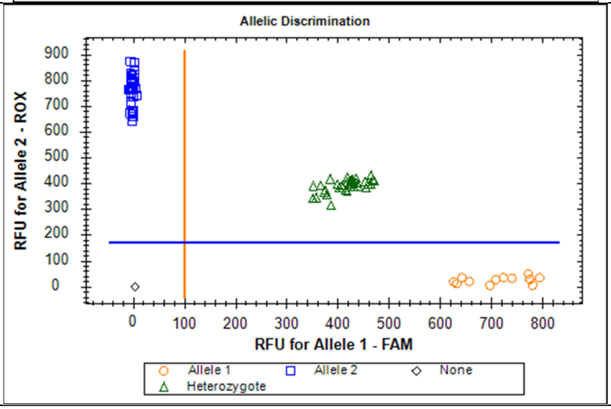

rs10892958<br>*HSPA8*	7	1161	0.006	0.994	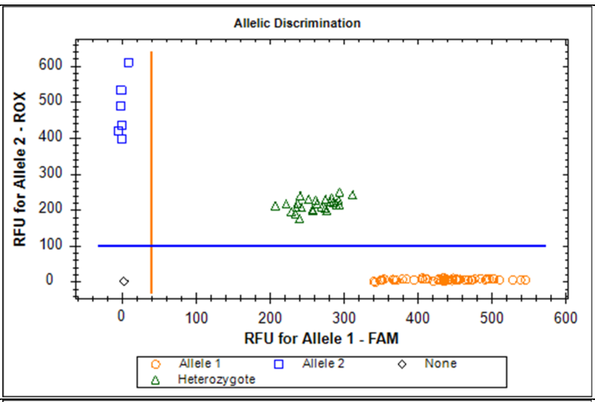
rs1136141<br>*HSPA8*	53	1161	0.046	0.954	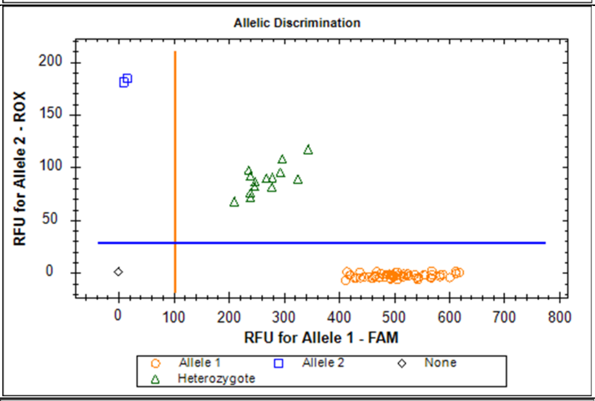
rs7155973<br>*HSP90AA1*	12	1161	0.010	0.990	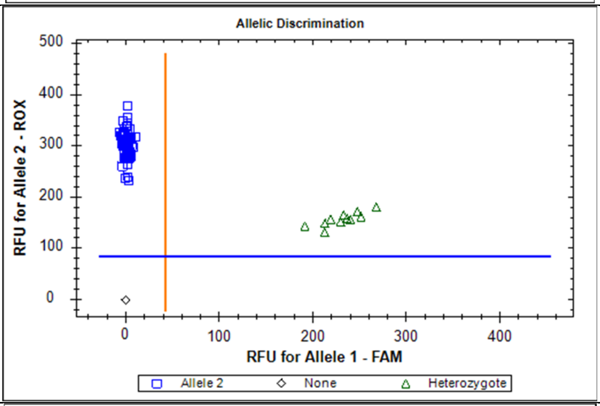
rs2034598<br>*DNAJA2*	6	1161	0.005	0.995	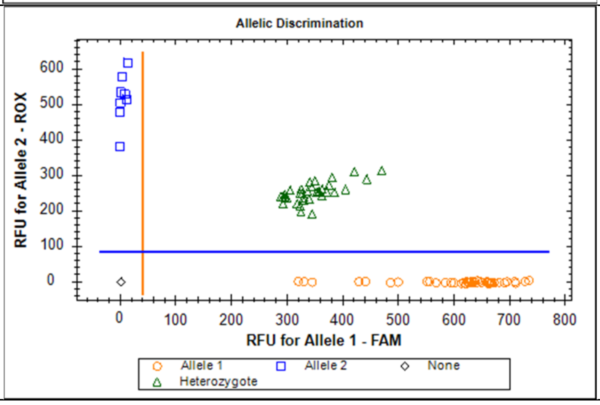

rs7189628 *DNAJA2*	23	1161	0.020	0.980	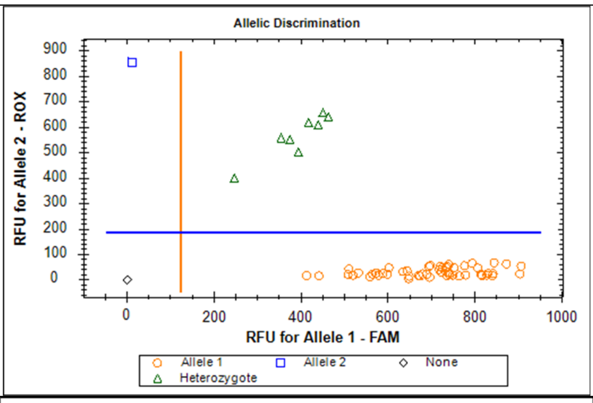
rs4926222 *DNAJB1*	5	1161	0.004	0.996	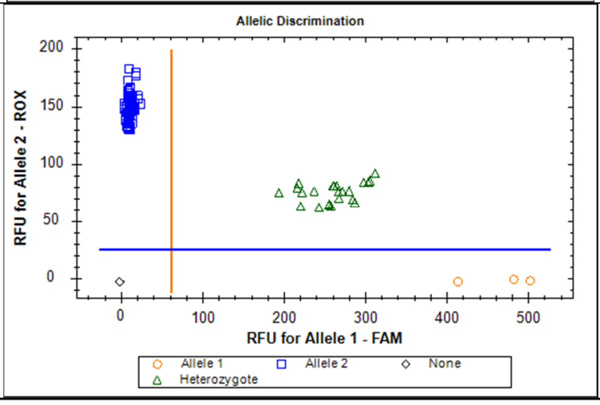
rs862832 *HSPA12B*	13	1161	0.011	0.989	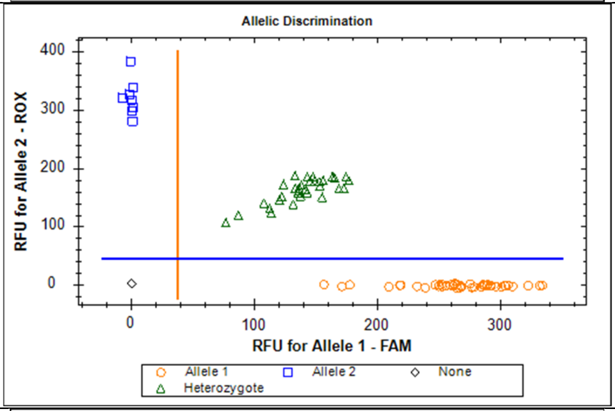
rs910652 *HSPA12B*	12	1161	0.010	0.990	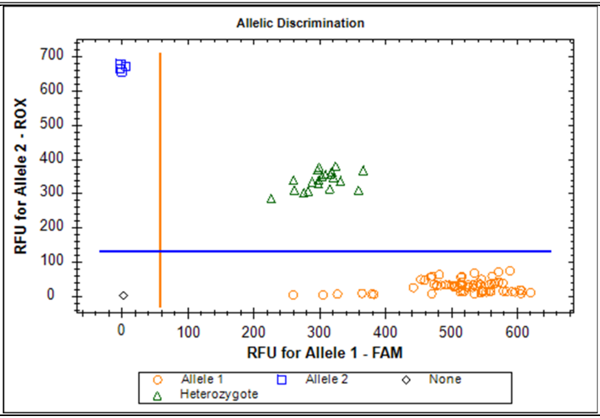

Table S2: Distribution of HSPs genotypes in COVID-19 patients/healthy controls and their correspondence to the Hardy-Weinberg equilibrium.

SNP	Genotypes	Controls		H_o $(H_e)^1$	P[2]	COVID-19 patients		H_o $(H_e)^3$	P[4]
rs753856 *HSPA6*	C/C	752	78.2%		>0.05	140	76.1%	0.23 (0.21)	>0.05
	C/G	199	20.7%			43	23.4%		

	G/G	11	1.1%	0.21 (0.20)		1	0.5%		
	MAF	0.11				0.12			
rs13161158 *HSPA4*	T/T	854	87.6%	0.12 (0.12)	>0.05	158	85.9%	0.14 (0.13)	>0.05
	T/C	117	12.0%			26	14.1%		
	C/C	4	0.4%			0	0.0%		
	MAF	0.06				0.07			
rs1042665 *HSPA9*	T/T	590	60.8%	0.35 (0.34)	>0.05	105	57.4%	0.33 (0.38)	>0.05
	T/C	335	34.5%			61	33.3%		
	C/C	45	4.6%			17	9.3%		
	MAF	0.22				0.26			
rs1043618 *HSPA1A*	G/G	397	40.8%	0.45 (0.46)	>0.05	66	36.1%	0.50 (0.48)	>0.05
	G/C	443	45.5%			91	49.7%		
	C/C	134	13.8%			26	14.2%		
	MAF	0.37				0.39			
rs6457452 *HSPA1B*	C/C	774	80.1%	0.18 (0.19)	>0.05	139	78.5%	0.18 (0.22)	**<0.05**
	T/C	175	18.1%			32	18.1%		
	T/T	17	1.8%			6	3.4%		
	MAF	0.11				0.12			
rs6909985 *HSF2*	G/G	704	72.5%	0.25 (0.25)	>0.05	125	68.7%	0.30 (0.28)	>0.05
	G/T	245	25.2%			54	29.7%		
	T/T	22	2.3%			3	1.6%		
	MAF	0.15				0.16			
rs4279640 *HSF1*	T/T	238	24.8%	0.50 (0.50)	>0.05	56	30.4%	0.45 (0.50)	>0.05
	T/C	484	50.4%			83	45.1%		
	C/C	239	24.9%			45	24.5%		
	MAF	0.50				0.47			
rs706121 *BAG1*	T/T	691	71.4%	0.25 (0.27)	**0.03**	124	67.8%	0.29 (0.29)	>0.05
	T/C	238	24.6%			53	29.0%		
	C/C	39	4.0%			6	3.3%		
	MAF	0.16				0.18			
rs17155992 *HSPA14*	G/G	828	86.1%	0.14 (0.13)	>0.05	151	83.0%	0.16 (0.16)	>0.05
	G/A	130	13.5%			30	16.5%		
	A/A	4	0.4%			1	0.5%		
	MAF	0.07				0.09			
rs196336 *BAG3*	C/C	329	33.8%	0.48 (0.49)	>0.05	58	31.7%	0.51 (0.49)	>0.05
	C/T	471	48.5%			94	51.4%		
	T/T	172	17.7%			31	16.9%		
	MAF	0.42				0.43			
rs196329 *BAG3*	G/G	560	57.4%	0.36 (0.37)	>0.05	110	61.1%	0.33 (0.35)	>0.05
	G/A	354	36.3%			59	32.8%		
	A/A	61	6.3%			11	6.1%		
	MAF	0.24				0.23			
rs1461496 *HSPA8*	G/G	394	40.5%	0.46 (0.46)	>0.05	69	37.5%	0.48 (0.47)	>0.05
	A/G	450	46.3%			89	48.4%		
	A/A	128	13.2%			26	14.1%		
	MAF	0.36				0.38			
rs10892958 *HSPA8*	C/C	621	63.7%	0.32 (0.32)	>0.05	112	62.6%	0.34 (0.32)	>0.05
	C/G	310	31.8%			61	34.1%		
	G/G	44	4.5%			6	3.4%		
	MAF	0.20				0.20			

rs1136141 *HSPA8*	G/G	688	74.5%	0.23 (0.24)	>0.05	129	70.1%	0.27 (0.28)	>0.05
	G/A	214	23.2%			49	26.6%		
	A/A	22	2.4%			6	3.3%		
	MAF	0.14				0.17			
rs7155973 *HSP90AA1*	G/G	839	86.3%	0.13 (0.13)	>0.05	153	86.4%	0.14 (0.13)	>0.05
	G/A	130	13.4%			24	13.6%		
	A/A	3	0.3%			0	0.0%		
	MAF	0.07				0.07			
rs2034598 *DNAJA2*	A/A	522	53.9%	0.38 (0.40)	>0.05	102	54.8%	0.38 (0.39)	>0.05
	A/G	368	38.0%			70	37.6%		
	G/G	79	8.2%			14	7.5%		
	MAF	0.27				0.26			
rs7189628 *DNAJA2*	C/C	871	91.2%	0.09 (0.08)	>0.05	151	82.5%	0.16 (0.17)	>0.05
	C/T	84	8.8%			30	16.4%		
	T/T	0	0.0%			2	1.1%		
	MAF	0.04				0.09			
rs4926222 *DNAJB1*	A/A	737	75.8%	0.22 (0.23)	>0.05	141	76.6%	0.20 (0.23)	>0.05
	A/G	216	22.2%			36	19.6%		
	G/G	19	2.0%			7	3.8%		
	MAF	0.13				0.14			
rs862832 *HSPA12B*	T/T	726	74.5%	0.24 (0.24)	>0.05	138	79.3%	0.18 (0.20)	>0.05
	T/C	230	23.6%			32	18.4%		
	C/C	18	1.8%			4	2.3%		
	MAF	0.14				0.11			
rs910652 *HSPA12B*	C/C	504	52.2%	0.4 (0.4)	>0.05	111	60.3%	0.33 (0.36)	>0.05
	C/T	382	39.6%			61	33.2%		
	T/T	79	8.2%			12	6.5%		
	MAF	0.28				0.23			

[1] – observed (Ho) and expected (He) heterozygosity in healthy controls; [2] – P-HWE in healthy controls; [3] – observed (Ho) and expected (He) heterozygosity in COVID-19 patients; [4] – P-HWE in COVID-19 patients, MAF – minor allele frequency.

Table S3: Results of the analysis of associations between *HSPs* SNPs and severe COVID-19 risk in groups stratified by sex, smoking status, physical activity levels, fresh fruit/vegetable intake, and age.

Genetic variant	Effect allele	Other allele	N	OR [95% CI][1]	P^2 (P_{bonf})	$P_{perm}{}^3$ (P_{bonf})	N	OR [95% CI][1]	P^2 (P_{bonf})	$P_{perm}{}^3$ (P_{bonf})
			Males				Females			
rs753856 *HSPA6*	G	C	480	0.98 [0.59-1.63]	0.93	1.00	666	1.16 [0.72-1.86]	0.54	0.59
rs13161158 *HSPA4*	C	T	485	1.26 [0.69-2.31]	0.45	0.55	674	0.94 [0.49-1.82]	0.85	1.00
rs1042665 *HSPA9*	C	T	483	1.24 [0.87-1.78]	0.23	0.18	670	1.24 [0.86-1.78]	0.25	0.22
rs1043618 *HSPA1A*	C	G	484	1.27 [0.91-1.77]	0.16	0.16	673	1.02 [0.74-1.4]	0.91	0.86
rs6457452 *HSPA1B*	T	C	479	1.4 [0.86-2.28]	0.18	0.28	664	1.01 [0.64-1.61]	0.95	1.00
rs6909985 *HSF2*	T	G	484	1.31 [0.85-2.02]	0.21	0.36	669	0.99 [0.64-1.53]	0.96	1.00
rs4279640 *HSF1*	C	T	481	0.81 [0.58-1.12]	0.21	0.18	664	0.96 [0.71-1.3]	0.79	0.86
rs706121 *BAG1*	C	T	482	1.1 [0.73-1.67]	0.64	0.70	669	1.1 [0.75-1.61]	0.64	0.7
rs17155992 *HSPA14*	A	G	478	1.14 [0.61-2.15]	0.68	0.86	666	1.38 [0.81-2.34]	0.24	0.31
rs196336 *BAG3*	T	C	485	0.92 [0.66-1.28]	0.63	0.60	670	1.13 [0.83-1.55]	0.43	0.37
rs196329 *BAG3*	A	G	482	0.85 [0.58-1.25]	0.41	0.35	673	0.94 [0.66-1.36]	0.76	0.86
rs1461496 *HSPA8*	A	G	484	1.17 [0.84-1.64]	0.36	0.38	672	1.02 [0.74-1.4]	0.91	0.86
rs10892958 *HSPA8*	G	C	483	1.05 [0.69-1.59]	0.82	0.86	671	0.96 [0.66-1.41]	0.85	0.86
rs1136141 *HSPA8*	A	G	465	1.27 [0.81-2.01]	0.30	0.23	643	1.17 [0.79-1.75]	0.43	0.5
rs7155973 *HSP90AA1*	A	G	479	0.88 [0.45-1.75]	0.72	0.70	670	1.04 [0.56-1.92]	0.91	0.86
rs2034598 *DNAJA2*	G	A	483	0.86 [0.59-1.24]	0.41	0.60	672	1.04 [0.75-1.46]	0.8	1.00
rs7189628 *DNAJA2*	T	C	473	**3.53 [1.9-6.56]**	**6.8×10^{-5}**	**7.6×10^{-5}**	665	1.56 [0.84-2.9]	0.16	0.15
rs4926222 *DNAJB1*	G	A	484	0.97 [0.6-1.58]	0.92	0.86	672	1.12 [0.73-1.71]	0.61	0.64
rs862832 *HSPA12B*	T	C	480	0.68 [0.39-1.16]	0.16	0.16	668	0.96 [0.6-1.53]	0.87	0.75
rs910652 *HSPA12B*	C	T	481	0.91 [0.63-1.31]	0.60	0.45	668	**0.68 [0.47-0.98]**	**0.04**	**0.04**
			Smokers				Nonsmokers			
rs753856 *HSPA6*	G	C	309	0.94 [0.48-1.86]	0.86	0.86	817	1.18 [0.78-1.77]	0.44	0.55
rs13161158 *HSPA4*	C	T	313	1.62 [0.81-3.23]	0.17	0.21	826	0.86 [0.47-1.57]	0.62	1.00
rs1042665 *HSPA9*	C	T	312	1.52 [0.96-2.43]	0.08	0.17	821	1.23 [0.9-1.67]	0.19	0.27
rs1043618 *HSPA1A*	C	G	313	1.11 [0.72-1.71]	0.64	0.86	824	1.09 [0.83-1.44]	0.52	0.60
rs6457452 *HSPA1B*	T	C	311	1.63 [0.9-2.95]	0.11	0.11	812	1.01 [0.67-1.53]	0.97	1.00

rs6909985 *HSF2*	T	G	313	1.43 [0.8-2.58]	0.23	0.24	821	1.04 [0.72-1.51]	0.82	1.00
rs4279640 *HSF1*	C	T	312	0.99 [0.65-1.5]	0.95	1.00	813	1.15 [0.88-1.5]	0.32	0.42
rs706121 *BAG1*	C	T	311	0.96 [0.56-1.64]	0.88	0.70	821	1.15 [0.82-1.61]	0.42	0.41
rs17155992 *HSPA14*	A	G	307	1.24 [0.56-2.74]	0.59	0.59	817	1.26 [0.78-2.04]	0.35	0.41
rs196336 *BAG3*	T	C	313	0.68 [0.43-1.06]	0.09	0.10	822	1.19 [0.9-1.56]	0.22	0.27
rs196329 *BAG3*	A	G	311	0.78 [0.45-1.35]	0.38	0.64	824	0.95 [0.69-1.29]	0.73	1.00
rs1461496 *HSPA8*	A	G	312	0.92 [0.6-1.41]	0.70	0.75	824	1.1 [0.84-1.46]	0.48	0.41
rs10892958 *HSPA8*	G	C	312	1.11 [0.66-1.88]	0.68	0.86	822	1 [0.71-1.39]	0.98	1.00
rs1136141 *HSPA8*	A	G	301	1.58 [0.88-2.85]	0.12	0.11	788	1.19 [0.83-1.69]	0.35	0.59
rs7155973 *HSP90AA1*	A	G	311	0.96 [0.4-2.3]	0.93	1.00	818	0.94 [0.54-1.64]	0.84	1.00
rs2034598 *DNAJA2*	G	A	312	1.1 [0.69-1.73]	0.70	0.86	823	0.93 [0.69-1.26]	0.65	0.86
rs7189628 *DNAJA2*	T	C	307	**3.99 [1.92-8.29]**	**0.0002**	**0.0003**	811	1.58 [0.9-2.78]	0.11	0.14
rs4926222 *DNAJB1*	G	A	313	1.18 [0.66-2.12]	0.58	0.86	823	1.01 [0.68-1.48]	0.97	1.00
rs862832 *HSPA12B*	T	C	311	0.9 [0.47-1.73]	0.76	1.00	817	0.83 [0.54-1.27]	0.39	0.33
rs910652 *HSPA12B*	C	T	310	0.75 [0.45-1.24]	0.26	0.31	819	0.79 [0.58-1.08]	0.15	0.15
			Low physical activity level				Normal physical activity level			
rs753856 *HSPA6*	G	C	1060	1.02 [0.65-1.63]	0.92 (1.0)	1.00 (1.0)	1042	1.17 [0.72-1.91]	0.53 (1.0)	0.75 (1.0)
rs13161158 *HSPA4*	C	T	1071	0.97 [0.53-1.8]	0.93 (1.0)	1.00 (1.0)	1057	1.26 [0.69-2.29]	0.45 (1.0)	0.64 (1.0)
rs1042665 *HSPA9*	C	T	1067	1.17 [0.83-1.66]	0.36 (0.72)	0.39 (0.78)	1050	**1.47 [1.03-2.1]**	**0.03** (0.06)	**0.02 (0.04)**
rs1043618 *HSPA1A*	C	G	1070	1.35 [1-1.81]	0.05 (0.1)	0.05 (0.1)	1055	0.85 [0.6-1.19]	0.34 (0.68)	0.27 (0.54)
rs6457452 *HSPA1B*	T	C	1058	**1.6 [1.08-2.37]**	**0.02 (0.04)**	**0.02 (0.04)**	1045	0.69 [0.38-1.25]	0.22 (0.44)	0.25 (0.5)
rs6909985 *HSF2*	T	G	1067	1.06 [0.7-1.59]	0.78 (1.0)	0.86 (1.0)	1051	1.22 [0.79-1.86]	0.37 (0.74)	0.41 (0.82)
rs4279640 *HSF1*	C	T	1057	0.98 [0.73-1.31]	0.88 (1.0)	0.86 (1.0)	1043	0.82 [0.6-1.13]	0.23 (0.46)	0.59 (1.0)
rs706121 *BAG1*	C	T	1064	0.99 [0.67-1.45]	0.95 (1.0)	1.00 (1.0)	1049	1.24 [0.84-1.82]	0.28 (0.56)	0.25 (0.50)

rs17155992 *HSPA14*	A	G	1057	1.27 [0.75-2.17]	0.37 (0.74)	0.32 (0.64)	1043	1.23 [0.69-2.2]	0.49 (1.0)	0.86 (1.0)
rs196336 *BAG3*	T	C	1068	1.17 [0.87-1.58]	0.30 (0.6)	0.28 (0.56)	1053	0.84 [0.6-1.16]	0.29 (0.58)	0.27 (0.54)
rs196329 *BAG3*	A	G	1070	1.05 [0.74-1.47]	0.80 (1.0)	0.86 (1.0)	1054	0.73 [0.49-1.1]	0.13 (0.26)	0.12 (0.24)
rs1461496 *HSPA8*	A	G	1069	0.88 [0.65-1.21]	0.44 (0.88)	0.59 (1.0)	1053	1.27 [0.92-1.76]	0.15 (0.3)	0.37 (0.74)
rs10892958 *HSPA8*	G	C	1069	1.02 [0.71-1.47]	0.91 (1.0)	1.00 (1.0)	1054	1.03 [0.69-1.53]	0.89 (1.0)	0.86 (1.0)
rs1136141 *HSPA8*	A	G	1021	1.25 [0.85-1.84]	0.26 (0.52)	0.31 (0.62)	1005	1.28 [0.84-1.95]	0.25 (0.50)	0.25 (0.50)
rs7155973 *HSP90AA1*	A	G	1065	0.91 [0.49-1.7]	0.78 (1.0)	1.00 (1.0)	1050	1.01 [0.53-1.93]	0.98 (1.0)	1.00 (1.0)
rs2034598 *DNAJA2*	G	A	1067	0.95 [0.68-1.31]	0.74 (1.0)	0.86 (1.0)	1051	1.01 [0.71-1.44]	0.94 (1.0)	1.00 (1.0)
rs7189628 *DNAJA2*	T	C	1050	**1.88 [1.05-3.34]**	**0.03** (0.6)	**0.02 (0.04)**	1037	**2.71 [1.52-4.84]**	**0.0007 (0.001)**	**0.0009 (0.002)**
rs4926222 *DNAJB1*	G	A	1069	0.77 [0.48-1.24]	0.28 (0.56)	0.22 (0.44)	1053	1.43 [0.94-2.16]	0.09 (0.18)	0.12 (0.24)
rs862832 *HSPA12B*	T	C	1065	1.15 [0.75-1.75]	0.52 (1.0)	0.60 (1.0)	1051	0.53 [0.29-0.98]	0.04 (0.08)	0.05 (0.1)
rs910652 *HSPA12B*	C	T	1061	0.96 [0.69-1.33]	0.79 (1.0)	0.75 (1.0)	1047	**0.58 [0.39-0.88]**	**0.009 (0.02)**	**0.007 (0.01)**
			Low fruit and vegetable intake				Normal fruit and vegetable intake			
rs753856 *HSPA6*	G	C	1076	1.08 [0.71-1.65]	0.72 (1.0)	1.00 (1.0)	1026	1.1 [0.64-1.91]	0.72 (1.0)	0.56 (1.0)
rs13161158 *HSPA4*	C	T	1089	1.03 [0.59-1.79]	0.92 (1.0)	1.00 (1.0)	1039	1.24 [0.63-2.43]	0.53 (1.0)	0.70 (1.0)
rs1042665 *HSPA9*	C	T	1083	1.12 [0.81-1.55]	0.50 (1.0)	0.52 (1.0)	1034	**1.67 [1.14-2.46]**	**0.009 (0.02)**	**0.009 (0.02)**
rs1043618 *HSPA1A*	C	G	1088	1.09 [0.83-1.44]	0.54 (1.0)	0.75 (1.0)	1037	1.11 [0.77-1.6]	0.59 (1.0)	0.86 (1.0)

rs6457452 *HSPA1B*	T	C	1076	1.37 [0.93-2.02]	0.11 (0.22)	0.09 (0.18)	1027	0.83 [0.45-1.53]	0.55 (1.0)	0.86 (1.0)
rs6909985 *HSF2*	T	G	1084	1.12 [0.77-1.63]	0.55 (1.0)	0.70 (1.0)	1034	1.14 [0.7-1.85]	0.59 (1.0)	0.52 (1.0)
rs4279640 *HSF1*	C	T	1075	0.91 [0.69-1.2]	0.52 (1.0)	0.86 (1.0)	1025	0.88 [0.62-1.26]	0.49 (1.0)	0.40 (0.80)
rs706121 *BAG1*	C	T	1082	0.93 [0.65-1.35]	0.72 (1.0)	1.00 (1.0)	1031	1.41 [0.93-2.14]	0.10 (0.20)	0.10 (0.20)
rs17155992 *HSPA14*	A	G	1075	1.26 [0.77-2.06]	0.36 (0.72)	0.60 (1.0)	1025	1.24 [0.65-2.38]	0.51 (1.0)	0.75 (1.0)
rs196336 *BAG3*	T	C	1086	1.04 [0.79-1.38]	0.76 (1.0)	0.86 (1.0)	1035	0.94 [0.65-1.36]	0.75 (1.0)	0.56 (1.0)
rs196329 *BAG3*	A	G	1087	0.87 [0.62-1.21]	0.41 (0.82)	0.39 (0.78)	1037	0.95 [0.62-1.45]	0.80 (1.0)	0.75 (1.0)
rs1461496 *HSPA8*	A	G	1086	0.95 [0.71-1.26]	0.71 (1.0)	0.70 (1.0)	1036	1.24 [0.86-1.78]	0.25 (0.5)	0.56 (1.0)
rs10892958 *HSPA8*	G	C	1087	1.3 [0.94-1.79]	0.11 (0.22)	0.13 (0.26)	1036	0.59 [0.35-1.01]	0.06 (0.12)	0.10 (0.20)
rs1136141 *HSPA8*	A	G	1039	**1.69 [1.2-2.36]**	**0.002 (0.004)**	**0.002 (0.004)**	987	0.6 [0.32-1.12]	0.11 (0.22)	0.14 (0.28)
rs7155973 *HSP90AA1*	A	G	1080	1.07 [0.62-1.84]	0.82 (1.0)	0.86 (1.0)	1035	0.78 [0.35-1.71]	0.53 (1.0)	0.86 (1.0)
rs2034598 *DNAJA2*	G	A	1084	0.87 [0.64-1.19]	0.38 (0.76)	0.42 (0.84)	1034	1.19 [0.81-1.73]	0.38 (0.76)	0.35 (0.7)
rs7189628 *DNAJA2*	T	C	1068	**2.39 [1.45-3.95]**	**0.0007 (0.001)**	**0.0008 (0.002)**	1019	1.92 [0.94-3.91]	0.07 (0.14)	0.05 (0.1)
rs4926222 *DNAJB1*	G	A	1086	0.93 [0.62-1.41]	0.74 (1.0)	0.75 (1.0)	1036	1.29 [0.8-2.09]	0.29 (0.58)	0.32 (0.64)
rs862832 *HSPA12B*	T	C	1082	0.98 [0.65-1.48]	0.93 (1.0)	0.86 (1.0)	1034	0.64 [0.34-1.2]	0.16 (0.32)	0.26 (0.52)
rs910652 *HSPA12B*	C	T	1079	0.8 [0.58-1.1]	0.17 (0.34)	0.13 (0.26)	1029	0.73 [0.47-1.11]	0.14 (0.28)	0.17 (0.34)
			Age<68				Age≥68			

rs753856 *HSPA6*	G	C	941	1.31 [0.83-2.05]	0.24	0.33	205	1.09 [0.55-2.15]	0.80	0.70
rs13161158 *HSPA4*	C	T	955	1.43 [0.83-2.48]	0.20	0.20	204	0.87 [0.36-2.08]	0.75	0.70
rs1042665 *HSPA9*	C	T	949	1.34 [0.94-1.91]	0.10	0.13	204	1.24 [0.79-1.93]	0.35	0.46
rs1043618 *HSPA1A*	C	G	954	0.89 [0.64-1.23]	0.48	0.35	203	**1.56 [1.04-2.35]**	**0.03**	**0.035**
rs6457452 *HSPA1B*	T	C	946	0.96 [0.59-1.55]	0.85	0.86	197	**2.29 [1.16-4.54]**	**0.02**	**0.01**
rs6909985 *HSF2*	T	G	951	1.28 [0.85-1.92]	0.23	0.38	202	0.93 [0.53-1.64]	0.81	0.86
rs4279640 *HSF1*	C	T	942	1.11 [0.81-1.51]	0.52	0.64	203	0.93 [0.64-1.36]	0.72	0.86
rs706121 *BAG1*	C	T	949	1.11 [0.76-1.62]	0.60	0.48	202	1.52 [0.88-2.63]	0.13	0.16
rs17155992 *HSPA14*	A	G	942	1.6 [0.96-2.66]	0.07	0.07	202	0.77 [0.36-1.66]	0.50	0.86
rs196336 *BAG3*	T	C	953	0.92 [0.67-1.27]	0.62	0.56	202	1.29 [0.87-1.91]	0.21	0.30
rs196329 *BAG3*	A	G	954	0.86 [0.59-1.25]	0.43	0.48	201	1.13 [0.71-1.78]	0.61	1.00
rs1461496 *HSPA8*	A	G	952	0.91 [0.66-1.26]	0.57	0.56	204	**1.59 [1.05-2.4]**	**0.03**	**0.03**
rs10892958 *HSPA8*	G	C	952	1.15 [0.79-1.67]	0.46	0.50	202	0.81 [0.49-1.33]	0.40	0.50
rs1136141 *HSPA8*	A	G	914	**1.55 [1.06-2.28]**	**0.02**	**0.02**	194	0.9 [0.5-1.61]	0.71	0.75
rs7155973 *HSP90AA1*	A	G	946	1.01 [0.54-1.88]	0.98	1.00	203	1.18 [0.5-2.77]	0.70	0.70
rs2034598 *DNAJA2*	G	A	951	0.99 [0.7-1.4]	0.96	1.00	204	0.76 [0.49-1.18]	0.22	0.36
rs7189628 *DNAJA2*	T	C	939	**2.02 [1.08-3.75]**	**0.03**	**0.02**	199	2.04 [0.96-4.36]	0.06	0.09
rs4926222 *DNAJB1*	G	A	952	1.11 [0.72-1.71]	0.65	0.75	204	0.85 [0.49-1.48]	0.58	0.50
rs862832 *HSPA12B*	T	C	951	0.61 [0.35-1.05]	0.07	0.06	197	1.14 [0.64-2.03]	0.66	0.70
rs910652 *HSPA12B*	C	T	946	0.78 [0.54-1.11]	0.17	0.20	203	0.63 [0.4-0.99]	0.047	0.08

All calculations were performed relative to the minor alleles (Effect allele); 1 - odds ratio and 95% confidence interval; 2– *P*- value; 3 - *P*- value after permutation testing; P_{bonf}– *P*-value after adjusting for multiple comparison where applicable; statistically significant differences are marked in bold.

Table S4: Associations of *HSPs* SNPs and clinical/laboratory parameters of COVID-19 patients.

SNP	Groups	Genotypes	N	Me [Q1-Q3]	Kruskal-Wallis-Test (P)	Mann–Whitney U test: P
Leukocytes						
rs2034598 *DNAJA2*	Entire group	A/A^1	102	11.2 [8.7; 14.7]	**0.025**	P$^{1-2}$=0.3
		A/G^2	71	12.6 [9; 16.7]		**P$^{1-3}$=0.007**
		G/G^3	15	15.5 [12.8; 17.6]		**P$^{2-3}$=0.06**
PTI						
rs1042665 *HSPA9*	Entire group	T/T^1	105	82 [73; 91]	**0.01**	P$^{1-2}$=0.8
		T/C^2	62	82.5 [73; 91]		**P$^{1-3}$=0.003**
		C/C^3	17	93 [83; 100]		**P$^{2-3}$=0.005**
Fibrinogen						
rs2034598 *DNAJA2*	Entire group	A/A^1	24	4.51 [3.68; 5.72]	**0.036**	**P$^{1-2}$=0.023**
		A/G^2	16	5.6 [5.1; 7.1]		P$^{1-3}$=1
		G/G^3	0			P$^{2-3}$=1
C-reactive protein						
rs753856 *HSPA6*	Entire group	C/C^1	140	94.8 [48.9; 150]	**0.026**	**P$^{1-2}$=0.01**
		C/G^2	43	64.7 [16; 115]		P$^{1-3}$=1
		G/G^3	0			P$^{2-3}$=1
Ground-glass opacity upon admission						
rs1136141 *HSPA8*	Entire group	G/G^1	130	49.5 [25; 60]	**0.01**	P$^{1-2}$=0.7
		A/G^2	50	45 [25; 58]		**P$^{1-3}$=0.002**
		A/A^3	6	9 [0; 15]		**P$^{2-3}$=0.003**
rs10892958 *HSPA8*	Entire group	C/C^1	113	50 [25; 60]	**0.01**	P$^{1-2}$=0.3
		C/G^2	62	46 [25; 60]		**P$^{1-3}$=0.003**
		G/G^3	6	9 [0; 15]		**P$^{2-3}$=0.002**
Ground-glass opacity upon discharge						
rs1136141 *HSPA8*	Entire group	G/G^1	121	38 [12; 50]	**0.025**	P$^{1-2}$=0.97
		A/G^2	49	35 [15; 50]		**P$^{1-3}$=0.008**
		A/A^3	5	4 [0; 10]		**P$^{2-3}$=0.007**
rs10892958 *HSPA8*	Entire group	C/C^1	105	38 [10; 50]	**0.015**	P$^{1-2}$=0.3
		C/G^2	60	40 [15; 50]		**P$^{1-3}$=0.01**
		G/G^3	5	4 [0; 10]		**P$^{2-3}$=0.004**
Oxygen therapy days						
rs6457452 *HSPA1B*	Entire group	C/C^1	141	3 [0; 5]	**0.03**	**P$^{1-2}$=0.02**
		C/T^2	32	4 [2; 8.5]		P$^{1-3}$=0.13
		T/T^3	6	7 [2; 12]		P$^{2-3}$=0.6
rs753856 *HSPA6*	Entire group	C/C^1	140	3 [0; 5]	**0.02**	**P$^{1-2}$=0.014**
		C/G^2	43	4 [2; 8]		P$^{1-3}$=1
		G/G^3	1	0		P$^{2-3}$=1
Days of lung ventilation						
rs706121 *BAG1*	Entire group	T/T^1	125	6 [3; 10]	**0.04**	**P$^{1-2}$=0.018**
		T/C^2	54	4 [1; 8]		P$^{1-3}$=0.3
		C/C^3	6	2.5 [2; 6]		P$^{2-3}$=0.9
Ttime to the start of clot growth (Tlag, minutes)						
rs196336 *BAG3*	Entire group	C/C^1	35	0.9 [0.8; 1]	**0.01**	P$^{1-2}$=0.08
		C/T^2	60	0.98 [0.83; 1.1]		**P$^{1-3}$=0.005**
		T/T^3	23	1 [0.9; 1.25]		P$^{2-3}$=0.09

rs196329 *BAG3*	Entire group	G/G[1]	69	0.9 [0.8; 1]	**0.001**	**P^{1-2}=0.001**
		A/G[2]	40	1 [0.9; 1.15]		**P^{1-3}=0.03**
		A/A[3]	7	1.1 [0.9; 1.3]		P^{2-3}=0.5
rs1461496 *HSPA8*	Entire group	G/G[1]	43	1 [0.9; 1.2]	**0.02**	**P^{1-2}=0.023**
		A/G[2]	61	0.9 [0.8; 1]		**P^{1-3}=0.02**
		A/A[3]	14	0.8 [0.8; 1.1]		P^{2-3}=0.4
rs6909985 *HSF2*	Entire group	G/G[1]	80	0.9 [0.8; 1.05]	**0.03**	**P^{1-2}=0.012**
		G/T[2]	34	1 [0.9; 1.15]		P^{1-3}=0.8
		T/T[3]	2	0.9 [0.8; 1]		P^{2-3}=0.3

Table S5: Cross-validation values of the most significant G×G interaction models associated with the development of severe COVID-19

Gene–gene interaction models	OR [95%CI]	Bal. Acc.	Se	Sp	CVC
Best two-locus models of intergenic interactions (for G×G models with $P_{min.} < 5\times10^{-5}$, 1000 permutations)					
**rs7189628 *DNAJA2*** × **rs2034598 *DNAJA2***	2.13 [1.05-4.32]	53.57	18.86	89.84	10/10
**rs7189628 *DNAJA2*** × **rs10892958 *HSPA8***	2.20 [1.08-4.50]	54.60	20.86	88.34	10/10
**rs7189628 *DNAJA2*** × **rs706121 *BAG1***	2.03 [1.06-3.94]	54.62	24.79	84.44	10/10
Best three-locus models of intergenic interactions (for G×G models with $P_{min.} < 1\times10^{-8}$, 1000 permutations)					
**rs7189628 *DNAJA2*** × **rs10892958 *HSPA8*** × **rs2034598 *DNAJA2***	2.41 [1.34-4.33]	57.33	31.62	83.04	10/10
Best four-locus models of gene-gene interactions (for models with $P_{min.} \leq$ 1×10-13, 1000 permutations)					
**rs4279640 *HSF1* × rs7189628 *DNAJA2*** × **rs10892958 *HSPA8*** × **rs706121 *BAG1***	2.34 [1.41-3.90]	59.28	49.46	68.17	10/10
**rs4279640 *HSF1*** × **rs7189628 *DNAJA2*** × **rs706121 *BAG1*** × rs1136141 *HSPA8*	2.34 [1.43-3.82]	59.85	57.53	61.15	10/10
Note: obtained by the GMDR method; OR [95% CI] – odds ratio with 95% confidence interval; Bal. Acc. (Balanced Accuracy) – model prediction accuracy; Se – model sensitivity; Sp – model specificity; CVC – model reproducibility (was 100%); a permutation test was performed for all models taking into account correction for covariates (age); 1000 permutations were performed with 10 cross-validations (CV), which ensures $P_{perm} \leq 0.001$; model reproducibility (CVC) as a result of the permutation test was 100%.					

Table S6: The most significant combinations of genotypes associated with severe COVID-19

Combinations of genotypes (1)	Beta (2)	P (3)	Risk (4)
**rs7189628 *DNAJA2* × rs2034598 *DNAJA2***			
rs7189628 *DNAJA2* T/T × rs2034598 *DNAJA2* A/A	0.83554	0.0013755	H
rs7189628 *DNAJA2* C/T × rs2034598 *DNAJA2* A/G	0.22538	0.0001757	H
**rs7189628 *DNAJA2* × rs10892958 *HSPA8***			
rs7189628 *DNAJA2* T/T × rs10892958 *HSPA8* C/C	0.84073	0.0011052	H
rs7189628 *DNAJA2* C/T × rs10892958 *HSPA8* C/G	0.23318	0.0004357	H
**rs7189628 *DNAJA2* × rs706121 *BAG1***			
rs7189628 *DNAJA2* C/T × rs706121 *BAG1* T/T	-0.040706	0.0734637	L
rs7189628 *DNAJA2* T/T × rs706121 *BAG1* T/T	0.850022	0.0210069	H
rs7189628 *DNAJA2* C/T × rs706121 *BAG1* T/C	0.261094	0.0005771	H
rs7189628 *DNAJA2* T/T× rs706121 *BAG1* T/C	0.822273	0.0255918	H
**rs7189628 *DNAJA2* × rs10892958 *HSPA8* × rs2034598 *DNAJA2***			
rs7189628 *DNAJA2* T/T × rs10892958 *HSPA8* C/C × rs2034598 *DNAJA2* A/A	0.840029	1.139e-03	H
rs7189628 *DNAJA2* C/T × rs10892958 *HSPA8* C/G × rs2034598 *DNAJA2* A/G	0.487459	6.216e-07	H
rs7189628 *DNAJA2* C/C× rs10892958 *HSPA8* G/G × rs2034598 *DNAJA2* A/G	-0.161458	7.059e-02	L
**rs4279640 *HSF1* × rs7189628 *DNAJA2* × rs10892958 *HSPA8* × rs706121 *BAG1***			
rs4279640 *HSF1* C/C × rs7189628 *DNAJA2* T/T × rs10892958 *HSPA8* C/C × rs706121 *BAG1* T/T	0.852263	1.997e-02	H
rs4279640 *HSF1* T/T × rs7189628 *DNAJA2* C/T × rs10892958 *HSPA8* C/G × rs706121 *BAG1* T/T	0.549438	7.265e-05	H
rs4279640 *HSF1* T/C × rs7189628 *DNAJA2* C/C× rs10892958 *HSPA8* G/G × rs706121 *BAG1* T/T	-0.161220	8.049e-02	L
rs4279640 *HSF1* C/C × rs7189628 *DNAJA2* C/T × rs10892958 *HSPA8* C/C × rs706121 *BAG1* T/C	0.596712	1.123e-03	H
rs4279640 *HSF1* T/C × rs7189628 *DNAJA2* T/T × rs10892958 *HSPA8* C/C × rs706121 *BAG1* T/C	0.824514	2.439e-02	H
rs4279640 *HSF1* T/C × rs7189628 *DNAJA2* C/T × rs10892958 *HSPA8* C/G × rs706121 *BAG1* T/C	0.345809	5.943e-02	H
rs4279640 *HSF1* C/C × rs7189628 *DNAJA2* C/T × rs10892958 *HSPA8* C/G × rs706121 *BAG1* T/C	0.825257	1.430e-03	H
rs4279640 *HSF1* T/T × rs7189628 *DNAJA2* C/C× rs10892958 *HSPA8* G/G × rs706121 *BAG1* T/C	0.852263	1.997e-02	H
rs4279640 *HSF1* T/T × rs7189628 *DNAJA2* C/T × rs10892958 *HSPA8* C/C × rs706121 *BAG1* C/C	0.824514	2.439e-02	H
**rs4279640 *HSF1* × rs7189628 *DNAJA2* × rs706121 *BAG1* × rs1136141 *HSPA8***			
rs4279640 *HSF1* C/C × rs7189628 *DNAJA2* T/T × rs706121 *BAG1* T/T × rs1136141 *HSPA8* G/G	0.841954	0.025034	H
rs4279640 *HSF1* C/C × rs7189628 *DNAJA2* C/T × rs706121 *BAG1* T/C × rs1136141 *HSPA8* G/G	0.586431	0.001805	H
rs4279640 *HSF1* T/C × rs7189628 *DNAJA2* T/T × rs706121 *BAG1* T/C × rs1136141 *HSPA8* G/G	0.814204	0.030246	H

rs4279640 *HSF1* T/T × rs7189628 *DNAJA2* C/T × rs706121 *BAG1* C/C × rs1136141 *HSPA8* G/G	0.814204	0.030246	H
rs4279640 *HSF1* T/T × rs7189628 *DNAJA2* C/T × rs706121 *BAG1* T/T × rs1136141 *HSPA8* A/G	0.420957	0.012375	H
rs4279640 *HSF1* C/C × rs7189628 *DNAJA2* C/T × rs706121 *BAG1* T/C × rs1136141 *HSPA8* A/G	0.814968	0.002146	H
rs4279640 *HSF1* C/C × rs7189628 *DNAJA2* C/C× rs706121 *BAG1* T/T × rs1136141 *HSPA8* A/A	0.593388	0.001589	H
rs4279640 *HSF1* C/C × rs7189628 *DNAJA2* C/T × rs706121 *BAG1* T/T × rs1136141 *HSPA8* A/A	0.841954	0.025034	H
rs4279640 *HSF1* T/T × rs7189628 *DNAJA2* C/C× rs706121 *BAG1* T/C × rs1136141 *HSPA8* A/A	0.841954	0.025034	H

Note: obtained by the MB-MDR method, taking into account correction for covariates; 1 – combination of genotypes;
2- beta – logistic regression coefficients for combinations of genotypes; 3 *p* – level of significance; 4 - Risk: H – high, L – low

Table S7: Cross-validation indices of the most significant G×E interaction models associated with the development of severe COVID-19

Gene–environment interaction models	OR [95%CI]	Bal. Acc.	Se	Sp	CVC
Best two-locus models of intergenic interactions (for G×E models with $P_{min.} < 5\times10^{-5}$, 1000 permutations)					
**rs7189628 *DNAJA2*** × SMOKE	2.07 [1.02-4.19]	53.93	16.67	91.17	10/10
Best three-locus models of intergenic interactions (for G×E models with $P_{min.} < 1\times10^{-8}$, 1000 permutations)					
**rs7189628 *DNAJA2*** × **rs2034598 *DNAJA2*** × SMOKE	2.26 [1.24-4.14]	56.06	34.34	77.79	10/10
**rs7189628 *DNAJA2*** × **rs196329 *BAG3*** × SMOKE	2.20 [1.26-3.85]	56.92	40.84	73.00	10/10
**rs7189628 *DNAJA2*** × rs4926222 *DNAJB1* × SMOKE	3.51 [1.60-7.70]	56.45	19.93	92.97	10/10
Best four-locus models of intergenic interactions (for G×E models with $P_{min.} \leq 1\times10^{-13}$, 1000 permutations)					
rs4279640 *HSF1* × **rs7189628 *DNAJA2*** × **rs196329 *BAG3*** × SMOKE	2.40 [1.45-3.95]	59.86	48.27	71.45	10/10
**rs7189628 *DNAJA2*** × **rs2034598 *DNAJA2*** × **rs1043618 *HSPA1A*** × SMOKE	2.33 [1.38-3.93]	58.40	48.04	68.76	10/10
**rs7189628 *DNAJA2*** × rs1042665 *HSPA9* × **rs1043618 *HSPA1A*** × SMOKE	2.38 [1.46-3.87]	60.24	33.57	65.98	10/10
Note: obtained by the GMDR method; OR [95% CI] – odds ratio with 95% confidence interval; Bal. Acc. (Balanced Accuracy) – model prediction accuracy; Se – model sensitivity; Sp – model specificity; CVC – model reproducibility (was 100%); a permutation test was performed for all models taking into account correction for covariates (age); 1000 permutations were performed with 10 cross-validations (CV), which ensures $P_{perm} \leq 0.001$; model reproducibility (CVC) as a result of the permutation test was 100%.					

Table S8: The most significant genotype-environment combinations associated with severe COVID-19

Combinations of genotypes (1)	Beta (2)	P (3)	Risk (4)
**rs7189628 *DNAJA2* × SMOKE**			
rs7189628 *DNAJA2* T/T × 0	0.82401	0.0246791	H
rs7189628 *DNAJA2* C/T × 1	0.21879	0.0006004	H
rs7189628 *DNAJA2* T/T × 1	0.85176	0.0202213	H
**rs7189628 *DNAJA2* × rs2034598 *DNAJA2* × SMOKE**			
rs7189628 *DNAJA2* T/T × rs2034598 *DNAJA2* A/A × 0	0.824064	2.465e-02	H
rs7189628 *DNAJA2* T/T × rs2034598 *DNAJA2* A/A × 1	0.851813	2.019e-02	H
rs7189628 *DNAJA2* C/T × rs2034598 *DNAJA2* A/G × 1	0.499993	2.390e-06	H
**rs7189628 *DNAJA2* × rs196329 *BAG3* × SMOKE**			
rs7189628 *DNAJA2* C/T × rs196329 *BAG3* A/A × 0	0.42166	0.0021307	H
rs7189628 *DNAJA2* T/T × rs196329 *BAG3* A/A × 0	0.82853	0.0223124	H
rs7189628 *DNAJA2* C/T × rs196329 *BAG3* G/G × 1	0.28850	0.0004099	H
rs7189628 *DNAJA2* T/T × rs196329 *BAG3* A/G × 1	0.85628	0.0181901	H
**rs7189628 *DNAJA2* × rs4926222 *DNAJB1* × SMOKE**			
rs7189628 *DNAJA2* T/T × rs4926222 *DNAJB1* A/A × 0	0.8260154	0.023595	H
rs7189628 *DNAJA2* C/T × rs4926222 *DNAJB1* A/G × 0	0.2655907	0.012090	H
rs7189628 *DNAJA2* C/T × rs4926222 *DNAJB1* A/A × 1	0.1942220	0.005373	H
rs7189628 *DNAJA2* T/T × rs4926222 *DNAJB1* A/A × 1	0.8537642	0.019289	H
rs7189628 *DNAJA2* C/C× rs4926222 *DNAJB1* G/G × 1	0.4327589	0.008091	H
rs7189628 *DNAJA2* C/T × rs4926222 *DNAJB1* G/G × 1	0.8260154	0.023595	H
**rs4279640 *HSF1* × rs7189628 *DNAJA2* × rs196329 *BAG3* × SMOKE**			
rs4279640 *HSF1* C/C × rs7189628 *DNAJA2* C/T × rs196329 *BAG3* A/A × 0	0.847807	5.176e-05	H
rs4279640 *HSF1* T/C × rs7189628 *DNAJA2* T/T × rs196329 *BAG3* A/A × 0	0.827769	2.273e-02	H
rs4279640 *HSF1* T/T × rs7189628 *DNAJA2* C/T × rs196329 *BAG3* G/G × 1	0.534638	3.307e-06	H
rs4279640 *HSF1* C/C × rs7189628 *DNAJA2* C/T × rs196329 *BAG3* A/G × 1	0.828521	1.252e-03	H
rs4279640 *HSF1* C/C × rs7189628 *DNAJA2* T/T × rs196329 *BAG3* A/G × 1	0.855518	1.855e-02	H

**rs7189628 *DNAJA2* × rs2034598 *DNAJA2* × rs1043618 *HSPA1A* × SMOKE**			
rs7189628 *DNAJA2* T/T × rs2034598 *DNAJA2* A/A × rs1043618 *HSPA1A* G/C × 0	0.8255709	2.385e-02	H
rs7189628 *DNAJA2* C/T × rs2034598 *DNAJA2* A/G × rs1043618 *HSPA1A* C/C × 0	0.8455910	5.945e-05	H
rs7189628 *DNAJA2* C/T × rs2034598 *DNAJA2* A/G × rs1043618 *HSPA1A* G/G × 1	0.4928242	1.958e-02	H
rs7189628 *DNAJA2* T/T × rs2034598 *DNAJA2* A/A × rs1043618 *HSPA1A* G/C × 1	0.8533198	1.951e-02	H
rs7189628 *DNAJA2* C/T × rs2034598 *DNAJA2* A/G × rs1043618 *HSPA1A* G/C × 1	0.5859720	5.686e-06	H
Note: obtained by the MB-MDR method, taking into account correction for covariates; 1 – combination of genotypes; 2- beta – logistic regression coefficients for combinations of genotypes; 3 *p* – level of significance; 4 - Risk: H – high, L – low			

Table S9: cis-eQTL effects for risk-associated HSP SNPs according to GTEx Portal.

Gene Symbol	Effect Allele	SNP Id	P-Value	NES	Tissue
HLA-DRB5	C	rs1043618 *HSPA1A*	5.90E-09	-0.34	Artery - Aorta
C4A	C	rs1043618 *HSPA1A*	6.50E-07	-0.22	Artery - Aorta
CYP21A1P	C	rs1043618 *HSPA1A*	1.5E-06	-0.29	Artery - Aorta
LY6G5C	C	rs1043618 *HSPA1A*	2.4E-06	-0.21	Artery - Aorta
HLA-DQB1	C	rs1043618 *HSPA1A*	0.000009	-0.22	Artery - Aorta
BAG6	C	rs1043618 *HSPA1A*	0.000012	-0.14	Artery - Aorta
C4B	C	rs1043618 *HSPA1A*	0.000021	0.18	Artery - Aorta
LY6G5B	C	rs1043618 *HSPA1A*	0.000037	-0.1	Artery - Aorta
HLA-B	C	rs1043618 *HSPA1A*	0.00011	0.14	Artery - Aorta
MICB	C	rs1043618 *HSPA1A*	0.00012	0.2	Artery - Aorta
VARS2	C	rs1043618 *HSPA1A*	0.00024	-0.17	Artery - Aorta
BAG6	C	rs1043618 *HSPA1A*	1.80E-21	-0.27	Artery - Tibial
HLA-DRB5	C	rs1043618 *HSPA1A*	9.20E-10	-0.27	Artery - Tibial
CYP21A1P	C	rs1043618 *HSPA1A*	1.50E-09	-0.27	Artery - Tibial
VARS2	C	rs1043618 *HSPA1A*	1.50E-08	-0.18	Artery - Tibial
C4A	C	rs1043618 *HSPA1A*	1.7E-06	-0.17	Artery - Tibial
LY6G5B	C	rs1043618 *HSPA1A*	2.3E-06	-0.081	Artery - Tibial
MICB	C	rs1043618 *HSPA1A*	4.8E-06	0.2	Artery - Tibial
SKIV2L	C	rs1043618 *HSPA1A*	0.000042	-0.14	Artery - Tibial
CYP21A2	C	rs1043618 *HSPA1A*	0.000046	0.19	Artery - Tibial
STK19	C	rs1043618 *HSPA1A*	0.00005	-0.12	Artery - Tibial
SNHG32	C	rs1043618 *HSPA1A*	0.000068	-0.1	Artery - Tibial
NOTCH4	C	rs1043618 *HSPA1A*	0.00018	-0.087	Artery - Tibial

LY6G5C	C	rs1043618 *HSPA1A*	2.20E-13	-0.28	Lung
HLA-DRB5	C	rs1043618 *HSPA1A*	1.50E-11	-0.36	Lung
C4A	C	rs1043618 *HSPA1A*	6.70E-11	-0.27	Lung
CYP21A1P	C	rs1043618 *HSPA1A*	2.90E-08	-0.29	Lung
CCHCR1	C	rs1043618 *HSPA1A*	1.4E-06	0.17	Lung
VARS2	C	rs1043618 *HSPA1A*	0.000002	-0.18	Lung
MICB	C	rs1043618 *HSPA1A*	3.6E-06	0.17	Lung
HCG22	C	rs1043618 *HSPA1A*	0.000011	-0.23	Lung
LY6G5B	C	rs1043618 *HSPA1A*	0.000046	-0.078	Lung
LY6G5C	C	rs1043618 *HSPA1A*	3.40E-24	-0.34	Whole Blood
C4B	C	rs1043618 *HSPA1A*	1.00E-15	0.4	Whole Blood
CYP21A1P	C	rs1043618 *HSPA1A*	6.40E-12	-0.3	Whole Blood
HLA-DRB5	C	rs1043618 *HSPA1A*	9.40E-10	-0.22	Whole Blood
C4A	C	rs1043618 *HSPA1A*	4.30E-09	-0.28	Whole Blood
CYP21A2	C	rs1043618 *HSPA1A*	8.70E-08	0.27	Whole Blood
LY6G5B	C	rs1043618 *HSPA1A*	3.10E-07	-0.085	Whole Blood
SNHG32	C	rs1043618 *HSPA1A*	1.6E-06	-0.069	Whole Blood
CCHCR1	C	rs1043618 *HSPA1A*	0.000017	0.11	Whole Blood
PRRC2A	C	rs1043618 *HSPA1A*	0.000023	-0.048	Whole Blood
VWA7	C	rs1043618 *HSPA1A*	0.000075	-0.15	Whole Blood
ENSG00000272501	C	rs1043618 *HSPA1A*	0.000086	0.08	Whole Blood
VARS2	C	rs1043618 *HSPA1A*	0.0001	-0.079	Whole Blood
HLA-C	C	rs1043618 *HSPA1A*	0.00029	0.1	Whole Blood
REEP2	C	rs1042665 *HSPA9*	0.000099	-0.12	Artery - Tibial
STK19B	T	rs6457452 *HSPA1B*	1.80E-08	0.69	Artery - Aorta

LSM2	T	rs6457452 *HSPA1B*	4.3E-06	-0.24	Artery - Aorta
VARS2	T	rs6457452 *HSPA1B*	0.000057	-0.36	Artery - Aorta
LSM2	T	rs6457452 *HSPA1B*	1.70E-08	-0.23	Artery - Tibial
STK19	T	rs6457452 *HSPA1B*	4.90E-07	-0.3	Artery - Tibial
VARS2	T	rs6457452 *HSPA1B*	5.3E-06	-0.29	Artery - Tibial
ATP6V1G2	T	rs6457452 *HSPA1B*	6.5E-06	0.35	Artery - Tibial
PSORS1C1	T	rs6457452 *HSPA1B*	0.00013	0.37	Artery - Tibial
STK19B	T	rs6457452 *HSPA1B*	3.50E-16	0.84	Lung
LSM2	T	rs6457452 *HSPA1B*	2.10E-08	-0.23	Lung
STK19	T	rs6457452 *HSPA1B*	1.20E-07	-0.38	Lung
LY6G5C	T	rs6457452 *HSPA1B*	1.9E-06	-0.3	Whole Blood
STK19	T	rs6457452 *HSPA1B*	0.00022	-0.15	Whole Blood
HLA-S	T	rs6457452 *HSPA1B*	0.00028	0.34	Whole Blood
HSPA12B	C	rs910652 *HSPA12B*	5.10E-10	0.3	Whole Blood
LINC01730	C	rs910652 *HSPA12B*	8.10E-08	0.24	Whole Blood
DNAJA2	C	rs7189628 *DNAJA2*	0.00072	0.11	Artery - Tibial

Table S10: cis-eQTL effects of HSP SNPs in whole blood according to eQTL Gene Browser

ID	Assessed	Other	Symbol	Z-score	P-value	FDR
rs1043618 *HSPA1A*	C	G	*HLA-DRB5*	-31.0505	1.12E-211	0
rs1043618 *HSPA1A*	C	G	*C4B*	24.6963	1.17E-134	0
rs1043618 *HSPA1A*	C	G	*LY6G5C*	-23.8935	3.57E-126	0
rs1043618 *HSPA1A*	C	G	*HSPA1B*	-15.2759	1.11E-52	0
rs1043618 *HSPA1A*	C	G	*C6orf48*	-14.4966	1.27E-47	0
rs1043618 *HSPA1A*	C	G	*CYP21A1P*	-13.1402	1.94E-39	0
rs1043618 *HSPA1A*	C	G	*HLA-DRB6*	-13.1111	2.84E-39	0
rs1043618 *HSPA1A*	C	G	*CSNK2B*	12.6476	1.15E-36	0

rs1043618 *HSPA1A*	C	G	*LY6G5B*	-11.9827	4.37E-33	0
rs1043618 *HSPA1A*	C	G	*AIF1*	-11.9534	6.22E-33	0
rs1043618 *HSPA1A*	C	G	*HLA-DQB1-AS1*	-11.6915	1.41E-31	0
rs1043618 *HSPA1A*	C	G	*DDAH2*	-11.3679	6.04E-30	0
rs1043618 *HSPA1A*	C	G	*CYP21A2*	10.9204	9.21E-28	0
rs1043618 *HSPA1A*	C	G	*HSPA1L*	-9.7603	1.67E-22	0
rs1043618 *HSPA1A*	C	G	*C4A*	-9.1168	7.74E-20	0
rs1043618 *HSPA1A*	C	G	*HLA-C*	9.1096	8.27E-20	0
rs1043618 *HSPA1A*	C	G	*CCHCR1*	9.0882	1.01E-19	0
rs1043618 *HSPA1A*	C	G	*VARS2*	-8.1535	3.53E-16	0
rs1043618 *HSPA1A*	C	G	*PRRC2A*	-7.7581	8.61E-15	0
rs1043618 *HSPA1A*	C	G	*VWA7*	-7.5709	3.70E-14	0
rs1043618 *HSPA1A*	C	G	*HCG22*	-6.9368	4.01E-12	0
rs1043618 *HSPA1A*	C	G	*HLA-DQB1*	-6.5237	6.85E-11	0
rs1043618 *HSPA1A*	C	G	*LSM2*	-6.4812	9.10E-11	0
rs1043618 *HSPA1A*	C	G	*PPT2*	6.4178	1.38E-10	0
rs1043618 *HSPA1A*	C	G	*PBX2*	-6.3836	1.73E-10	0
rs1043618 *HSPA1A*	C	G	*TNXA*	-6.2749	3.50E-10	0
rs1043618 *HSPA1A*	C	G	*HCG27*	6.2057	5.45E-10	0
rs1043618 *HSPA1A*	C	G	*XXbac-BPG248L24.11*	6.1676	6.93E-10	6.6E-06
rs1043618 *HSPA1A*	C	G	*APOM*	-6.0781	1.22E-09	1.31E-05
rs1043618 *HSPA1A*	C	G	*C2*	5.8076	6.34E-09	4.52E-05
rs1043618 *HSPA1A*	C	G	*AGER*	5.7895	7.06E-09	4.52E-05
rs1043618 *HSPA1A*	C	G	*DOM3Z*	-5.6022	2.11E-08	0.000109
rs1043618 *HSPA1A*	C	G	*HLA-DRA*	5.3229	1.02E-07	0.000327

rs1043618 *HSPA1A*	C	G	*STK19P*	5.2638	1.41E-07	0.000433
rs1043618 *HSPA1A*	C	G	*TNF*	-4.9918	5.98E-07	0.001819
rs1043618 *HSPA1A*	C	G	*MICB*	4.8166	1.46E-06	0.00409
rs1043618 *HSPA1A*	C	G	*XXbac-BPG299F13.16*	4.5519	5.319E-06	0.014265
rs1043618 *HSPA1A*	C	G	*HLA-DQA1*	-4.4172	9.99E-06	0.02587
rs1043618 *HSPA1A*	C	G	*DDX39B*	-4.3379	1.438E-05	0.036806
rs7189628 *DNAJA2*	T	C	*RP11-169E6.1*	-7.9132	2.51E-15	0
rs7189628 *DNAJA2*	T	C	*NETO2*	7.0376	1.96E-12	0
rs7189628 *DNAJA2*	T	C	*DNAJA2*	-5.6849	1.31E-08	7.07E-05
rs7189628 *DNAJA2*	T	C	*PHKB*	-4.995	5.88E-07	0.001776
rs7189628 *DNAJA2*	T	C	*GPT2*	4.5014	6.751E-06	0.017816
rs910652 *HSPA12B*	C	T	*C20orf27*	-17.7964	7.51E-71	0
rs910652 *HSPA12B*	C	T	*CDC25B*	12.5327	4.95E-36	0
rs910652 *HSPA12B*	C	T	*HSPA12B*	8.1087	5.12E-16	0
rs910652 *HSPA12B*	C	T	*PANK2*	-4.3672	1.259E-05	0.032481
rs1136141 *HSPA8*	A	G	*HSPA8*	8.1852	2.72E-16	0
rs1461496 *HSPA8*	A	G	*CRTAM*	5.1885	2.12E-07	0.00065
rs1042665 *HSPA9*	C	T	*KDM3B*	13.6722	1.49E-42	0
rs1042665 *HSPA9*	C	T	*ETF1*	-10.1477	3.39E-24	0
rs1042665 *HSPA9*	C	T	*FAM53C*	-4.432	9.328E-06	0.024184
rs1042665 *HSPA9*	C	T	*DNAJC18*	4.316	1.589E-05	0.040278
rs1042665 *HSPA9*	C	T	*KLHL3*	-4.2901	1.785E-05	0.044909

Table S11: The impact of *HSPs* SNPs on histone tags in various tissues (HaploReg data)

SNP (Ref/Alt allele)	Tissues Marks	Lung	Vessels – aorta	Blood
	H3K4me1	Enh	-	Enh

rs1461496<br>*HSPA8*<br>(G/**A**)	H3K4me3	Pro	Pro	Pro
	H3K27ac	Enh	Enh	Enh
	H3K9ac	-	-	Pro
	DNase	-	-	DNase
rs1136141<br>*HSPA8*<br>(G/**A**)	H3K4me1	-	-	Enh
	H3K4me3	Pro	Pro	Pro
	H3K27ac	Enh	Enh	Enh
	H3K9ac	-	-	Pro
	DNase	-	-	DNase
rs1042665<br>*HSPA9*<br>(T/**C**)	H3K4me1	Enh	-	Enh
	H3K4me3	-	-	Pro
	H3K27ac	-	-	Enh
	H3K9ac	-	-	Pro
rs6457452<br>*HSPA1B*<br>(C/**T**)	H3K4me1	Enh	-	Enh
	H3K4me3	Pro	Pro	Pro
	H3K27ac	Enh	Enh	Enh
	H3K9ac	-	-	Pro
	DNase	-	-	DNase
rs910652<br>*HSPA12B*<br>(T/**C**)	H3K4me1	-	-	Enh
	H3K9ac	-	-	Pro
rs1043618<br>*HSPA1A*<br>(G/**C**)	H3K4me1			Enh
	H3K4me3	Pro	Pro	Pro
	H3K27ac	Enh	Enh	Enh
	H3K9ac			Pro
	DNase			DNase
rs7189628<br>*DNAJA2*<br>(C/**T**)	H3K4me1	Enh	Enh	Enh
	H3K4me3	-	-	Pro
	H3K27ac	-	-	Enh
	H3K9ac	-	-	Pro

H3K4me1 – mono-methylation at the 4th lysine residue of the histone H3 protein; H3K4me3 – tri-methylation at the 4th lysine residue of the histone H3 protein; H3K9ac – the acetylation at the 9th lysine residues of the histone H3 protein; H3K27ac – acetylation of the lysine residues at N-terminal position 27 of the histone H3 protein; effect alleles are marked in bold.
Enh – histone modification in the enhancer region; Pro – histone modification at the promoter region.

Table S12: Results of aggregated bioinformatic analyses of associations between SNPs and the risk of severe COVID-19.

№	SNP	Phenotype	*P* Value	Beta (OR)	Sample Size
1.	rs1461496<br>*HSPA8*<br>(G/**A**)	-			
2.<br>3.	rs1136141<br>*HSPA8*<br>(G/**A**)	Peak expiratory flow	0.004221	Beta ▼-0.0106	690530
4.		Forced expired volume in 1 second (FEV1)	0.004895	Beta ▼-0.0097	793446

5.		Forced vital capacity (FVC)	0.01485	Beta ▼ -0.0082	792940
6.	rs1042665 *HSPA9* (T/**C**)	-			
7.	rs6457452 *HSPA1B* (C/**T**)	Hospitalized COVID-19 vs population	0.002841	OR ▲ 1.0384	821852
8.		Forced vital capacity (FVC) in idiopathic pulmonary fibrosis	0.005571	Beta ▼ -0.2686	1048
9.					
10.		COVID-19 vs population	0.006677	OR ▲ 1.0118	1447230
11.		Peak expiratory flow	0.00212	Beta ▼ -0.0122	690530
12.		Very severe respiratory confirmed COVID-19 vs population	0.0025335	OR ▲ 1.0537	69528
13.		Very severe COVID-19 vs population and mild COVID-19 cases	0.04599	OR ▲ 1.4054	95491
14.		Hospitalized COVID-19 vs population and mild COVID-19 cases	0.04599	OR ▲ 1.4054	179045
15.	rs910652 *HSPA12B* (T/**C**)	Very severe respiratory confirmed COVID-19 vs population	0.018885	OR ▼ 0.9660	62656
16.	rs1043618 *HSPA1A* (G/**C**)	FEV1 to FVC ratio	1.52e-15	Beta ▼ -0.0154	571162
17.		Peak expiratory flow	9.56e-12	Beta ▼ -0.0160	382560
18.		Forced expired volume in 1 second (FEV1)	1.49e-7	Beta ▼ -0.0101	571351
19.	rs7189628 *DNAJA2* (C/**T**)	-			

data obtained using the bioinformatic resource Lung Disease Knowledge Portal https://lung.hugeamp.org/
Effect alleles are marked in bold

Table S13: Analysis of the effect of rs1461496 *HSPA8* on the binding of DNA to transcription factors.

№	Ref/SNP allele[1]	TF[2]	GAIN /LOSS[3]	Motif[4]	P-Value SNP impact[5]	P-Value Ref[6]	P-Value SNP[7]
1	A/G	SMAD	gain	SMAD_2	0.002	0.216	0.007
2	A/G	SMAD	gain	SMAD_1	0.002	0.705	0.007
3	A/G	SMAD3	gain	SMAD3_2	0.003	0.240	0.009
4	A/G	SMAD4	gain	SMAD4_1	0.007	0.187	0.010
5	A/G	ATF1	gain	ATF1_3	0.011	0.170	0.012
6	A/G	ESR1	gain	ESR1_3	0.009	0.099	0.014
7	A/G	ESR1	gain	MA0112.2	0.008	0.100	0.014
8	A/G	Bach1::Mafk	gain	MA0591.1	0.009	0.140	0.017
9	A/G	RFX4	gain	RFX4_3	0.019	0.152	0.019
10	A/G	HDAC2	gain	HDAC2_disc4	0.010	0.488	0.020

11	A/G	SMAD3	gain	SMAD3_1	0.005	0.233	0.023
12	A/G	MEIS2	gain	MEIS2_3	0.014	0.531	0.025
13	A/G	ESRRA	gain	ESRRA_disc1	0.021	0.149	0.025
14	A/G	SRF	gain	SRF_2	0.019	0.254	0.026
15	A/G	MEIS1	gain	MEIS1_4	0.011	0.904	0.026
16	A/G	RFX3	gain	RFX3_3	0.004	0.932	0.028
17	A/G	FOSL2	gain	MA0478.1	0.028	0.239	0.032
18	A/G	RFX4	gain	RFX4_1	0.022	0.257	0.033
19	A/G	RFX2	gain	RFX2_4	0.030	0.203	0.037
20	A/G	HDAC2	gain	HDAC2_disc1	0.015	0.529	0.038
21	A/G	RFX3	gain	RFX3_1	0.024	0.267	0.040
22	A/G	MEIS3	gain	MEIS3_4	0.014	0.809	0.050
23	A/G	PBX1	loss	PBX1_3	0.002	0.001	0.052
24	A/G	ZEB1	loss	ZEB1_4	0.0001	0.002	0.079
25	A/G	SOX9	loss	ZEB1_4	0.0001	0.002	0.079
26	A/G	E2F3	loss	E2F3_4	0.010	0.013	0.091
27	A/G	SP1	loss	SP1_disc2	0.001	0.006	0.126
28	A/G	FOXG1	loss	SP1_disc2	0.001	0.006	0.126
29	A/G	POU1F1	loss	POU1F1_1	0.001	0.010	0.136
30	A/G	AIRE	loss	AIRE_2	0.005	0.037	0.149
31	A/G	FOXA	loss	FOXA_disc5	0.016	0.021	0.166
32	A/G	LEF1	loss	LEF1_1	0	0.007	0.173
33	A/G	E2F2	loss	LEF1_1	0	0.007	0.173
34	A/G	CACD	loss	CACD_2	0.006	0.018	0.194
35	A/G	BATF	loss	BATF_disc2	0.021	0.027	0.210
36	A/G	AHR::ARNT	loss	AHR::ARNT_3	0.004	0.026	0.230
37	A/G	FOXF1	loss	FOXF1_1	0.004	0.018	0.233
38	A/G	STAT	loss	STAT_disc4	0.032	0.046	0.264
39	A/G	FOXA2	loss	FOXA2_1	0.003	0.012	0.307
40	A/G	Foxd3	loss	MA0041.1	0.025	0.049	0.323
41	A/G	FOXI1	loss	FOXI1_2	0.014	0.039	0.358
42	A/G	FOXD1	loss	MA0031.1	0.032	0.037	0.358
43	A/G	FOXI1	loss	FOXI1_1	0.011	0.042	0.396
44	A/G	FOXI1	loss	MA0042.1	0.001	0.032	0.400
45	A/G	SOX	loss	MA0042.1	0.001	0.032	0.400
46	A/G	PBX1	loss	PBX1_1	0.005	0.023	0.422
47	A/G	FOXO4	loss	FOXO4_1	0.003	0.025	0.436
48	A/G	FOXO1	loss	FOXO1_1	0.018	0.028	0.462
49	A/G	PAX2	loss	PAX2_2	0.014	0.045	0.562
50	A/G	DMRTA1	loss	DMRTA1_1	0.011	0.033	0.601
51	A/G	LEF1	loss	LEF1_3	0.006	0.037	0.705
52	A/G	SRY	loss	SRY_1	0.009	0.019	0.718
53	A/G	FOXJ2	loss	FOXJ2_4	0.009	0.042	0.822
54	A/G	FOXL1	loss	FOXL1_4	0.004	0.017	0.907
	G[8]	regulation of transforming growth factor beta2 production (GO:0032909; FDR = 0.0025); positive regulation of extracellular matrix assembly (GO:1901203; FDR = 0.00681); activin receptor signaling pathway (GO:0032924; FDR = 0.021); positive regulation of epithelial to mesenchymal transition (GO:0010718; FDR = 0.00153); positive regulation of nitric oxide biosynthetic process (GO:0045429; FDR = 0.0328);					

		regulation of transforming growth factor beta receptor signaling pathway (GO:0017015; FDR = 0.0149); cellular response to transforming growth factor beta stimulus (GO:0071560; FDR = 0.0176); SMAD protein signal transduction (GO:0060395; FDR = 0.019); response to hypoxia (GO:0001666; FDR = 0.00637).
	A[9]	epithelial tube branching involved in lung morphogenesis (GO:0060441; FDR = 0.0402); lymphocyte differentiation (GO:0030098; FDR = 0.0385); negative regulation of epithelial cell differentiation (GO:0030857; FDR = 0.00389); positive regulation by host of viral transcription (GO:0043923; FDR = 0.0209); canonical Wnt signaling pathway (GO:0060070; FDR = 0.00144).

1 – reference (Ref) / alternative (SNP) allele;
2 – TF - transcription factor;
3 – binding of TF to the reference (LOSS) / alternative (GAIN) allele;
4 – binding sites with high affinity for TF;
5 – p value statistically confirming the potential gain or loss of function of the genomic region with SNP in terms of transcription factor binding;
6 – p-value for assessing the binding of TF to the Ref allele;
7 – p-value for assessing the binding of TF to the SNP allele;
8 – biological processes pathogenetically significant for IS, in which TFs that bind to the SNP allele are jointly involved (data from the Gene Ontology resource; http://geneontology.org/);
9 – biological processes pathogenetically significant for IS, in which TFs that bind to reference allele are jointly involved (data from the Gene Ontology resource; http://geneontology.org/)

Table S14. Analysis of the effect of rs1136141 *HSPA8* on the binding of DNA to transcription factors.

№	Ref/SNP allele[1]	TF[2]	GAIN /LOSS[3]	Motif[4]	P-Value SNP impact[5]	P-Value Ref[6]	P-Value SNP[7]
1	G/A	TEAD1	gain	TEAD1_2	0.002	0.086	0.005
2	G/A	EGR1	gain	TEAD1_2	0.002	0.086	0.005
3	G/A	FEV	gain	FEV_1	0.001	0.169	0.006
4	G/A	KLF12	gain	FEV_1	0.001	0.169	0.006
5	G/A	TEAD1	gain	TEAD1_1	0.006	0.144	0.006
6	G/A	TEAD1	gain	MA0090.1	0.0001	0.108	0.007
7	G/A	NFATC1	gain	MA0090.1	0.0001	0.108	0.007
8	G/A	TEAD1	gain	TEAD1_3	0.011	0.264	0.014
9	G/A	EGR1	gain	EGR1_disc4	0.001	0.094	0.014
10	G/A	RREB1	gain	EGR1_disc4	0.001	0.094	0.014
11	G/A	Stat5a::Stat 5b	gain	MA0519.1	0.010	0.195	0.017
12	G/A	GATA2	gain	GATA2_2	0.019	0.238	0.019
13	G/A	TEAD3	gain	TEAD3_2	0.009	0.284	0.019
14	G/A	E2F1	gain	E2F1_10	0.009	0.306	0.020
15	G/A	CREB1	gain	CREB1_8	0.007	0.517	0.021
16	G/A	IKZF2	gain	IKZF2_1	0.005	0.519	0.024
17	G/A	STAT	gain	STAT_disc1	0.009	0.313	0.039
18	G/A	IKZF1	gain	IKZF1_1	0.020	0.360	0.047
19	G/A	KLF5	loss	MA0599.1	0.009	0.003	0.058
20	G/A	PPARA	loss	PPARA_1	0.011	0.012	0.059

21	G/A	EBF1	loss	EBF1_1	0.004	0.011	0.077
22	G/A	TFAP2	loss	EBF1_1	0.004	0.011	0.077
23	G/A	KLF4	loss	KLF4_1	0.002	0.005	0.085
24	G/A	ZNF524	loss	KLF4_1	0.002	0.005	0.085
25	G/A	CTCF	loss	CTCF_disc6	0.019	0.007	0.087
26	G/A	TATA	loss	TATA_disc4	0.004	0.005	0.088
27	G/A	AHR	loss	TATA_disc4	0.004	0.005	0.088
28	G/A	Klf4	loss	MA0039.2	0.004	0.007	0.089
29	G/A	E4F1	loss	MA0039.2	0.004	0.007	0.089
30	G/A	EBF1	loss	EBF1_4	0.008	0.009	0.100
31	G/A	Klf1	loss	MA0493.1	0.007	0.004	0.116
32	G/A	NR2C2	loss	NR2C2_disc3	0.005	0.012	0.138
33	G/A	SP1	loss	NR2C2_disc3	0.005	0.012	0.138
34	G/A	EGR1	loss	EGR1_disc5	0.017	0.033	0.156
35	G/A	PAX4	loss	PAX4_1	0.000	0.00001	0.165
36	G/A	ATF3	loss	PAX4_1	0.000	0.00001	0.165
37	G/A	SP3	loss	SP3_2	0.002	0.006	0.178
38	G/A	ELF5	loss	SP3_2	0.002	0.006	0.178
39	G/A	RREB1	loss	RREB1_1	0.001	0.019	0.184
40	G/A	KLF7	loss	RREB1_1	0.001	0.019	0.184
41	G/A	RREB1	loss	RREB1_2	0.003	0.041	0.184
42	G/A	ZNF589	loss	RREB1_2	0.003	0.041	0.184
43	G/A	SP1	loss	SP1_2	0.018	0.029	0.188
44	G/A	EGR1	loss	EGR1_disc1	0.010	0.022	0.188
45	G/A	SP1	loss	SP1_3	0.008	0.014	0.194
46	G/A	NR2F2	loss	NR2F2_1	0.005	0.034	0.207
47	G/A	TBX1	loss	TBX1_1	0.012	0.027	0.213
48	G/A	EGR1	loss	EGR1_disc6	0.015	0.023	0.217
49	G/A	SP1	loss	SP1_7	0.007	0.015	0.224
50	G/A	ZNF281	loss	ZNF281_1	0.012	0.038	0.228
51	G/A	WT1	loss	WT1_1	0.018	0.041	0.263
52	G/A	REST	loss	REST_disc8	0.004	0.024	0.301
53	G/A	BCL	loss	REST_disc8	0.004	0.024	0.301
54	G/A	HEY1	loss	HEY1_disc2	0.000	0.001	0.307
55	G/A	SP1	loss	HEY1_disc2	0.000	0.001	0.307
56	G/A	TBX5	loss	TBX5_1	0.005	0.015	0.319
57	G/A	RUNX1	loss	RUNX1_7	0.013	0.035	0.376
58	G/A	RUNX3	loss	RUNX3_2	0.018	0.029	0.389
59	G/A	IRF	loss	IRF_disc4	0.005	0.029	0.415
60	G/A	RUNX3	loss	RUNX3_4	0.012	0.033	0.436
61	G/A	RUNX1	loss	RUNX1_9	0.019	0.044	0.445
62	G/A	KLF16	loss	KLF16_1	0.003	0.026	0.454
63	G/A	ETS1	loss	KLF16_1	0.003	0.026	0.454
64	G/A	RUNX2	loss	RUNX2_6	0.003	0.021	0.467
65	G/A	GLIS2	loss	RUNX2_6	0.003	0.021	0.467
66	G/A	GLIS1	loss	GLIS1_1	0.002	0.024	0.535
67	G/A	ETS	loss	GLIS1_1	0.002	0.024	0.535
68	G/A	PAX4	loss	PAX4_3	0.001	0.008	0.958
69	G/A	EGR3	loss	PAX4_3	0.001	0.008	0.958

	A[8]	hippo signaling (GO:0035329; FDR = 0.0219); leukocyte differentiation (GO:0002521; FDR = 0.00251).
	G[9]	positive regulation of CD8-positive, alpha-beta T cell differentiation (GO:0043378; FDR = 0.0043); regulation of leukocyte cell-cell adhesion (GO:1903037; FDR = 0.00615); response to cAMP (GO:0051591; FDR = 0.0452); negative regulation of leukocyte cell-cell adhesion (GO:1903038; FDR = 0.0121).

1 – reference (Ref) / alternative (SNP) allele;
2 – TF - transcription factor;
3 – binding of TF to the reference (LOSS) / alternative (GAIN) allele;
4 – binding sites with high affinity for TF;
5 – p value statistically confirming the potential gain or loss of function of the genomic region with SNP in terms of transcription factor binding;
6 – p-value for assessing the binding of TF to the Ref allele;
7 – p-value for assessing the binding of TF to the SNP allele;
8 – biological processes pathogenetically significant for IS, in which TFs that bind to the SNP allele are jointly involved (data from the Gene Ontology resource; http://geneontology.org/);
9 – biological processes pathogenetically significant for IS, in which TFs that bind to reference allele are jointly involved (data from the Gene Ontology resource; http://geneontology.org/)

Table S15: Analysis of the effect of rs1042665 *HSPA9* on the binding of DNA to transcription factors

№	Ref/SNP allele[1]	TF[2]	GAIN /LOSS[3]	Motif[4]	P-Value SNP impact[5]	P-Value Ref[6]	P-Value SNP[7]
1	T/C	TBX20	gain	TBX20_3	0.00001	0.455	0.0002
2	T/C	SRY	gain	TBX20_3	0.00001	0.455	0.0002
3	T/C	TBX21	gain	TBX21_2	0	0.059	0.0003
4	T/C	FOXC2	gain	TBX21_2	0	0.059	0.0003
5	T/C	TBX20	gain	TBX20_4	0.0001	0.283	0.0004
6	T/C	TBX20	gain	TBX20_4	0.0001	0.283	0.0004
7	T/C	TBX1	gain	TBX1_3	0.0001	0.333	0.0004
8	T/C	FOXC1	gain	TBX1_3	0.0001	0.333	0.0004
9	T/C	TBR1	gain	TBR1_2	0.0001	0.546	0.0004
10	T/C	PRDM1	gain	TBR1_2	0.0001	0.546	0.0004
11	T/C	MGA	gain	MGA_1	0.0002	0.114	0.001
12	T/C	FOXD3	gain	MGA_1	0.0002	0.114	0.001
13	T/C	TBX4	gain	TBX4_1	0.001	0.084	0.001
14	T/C	MEF2A	gain	TBX4_1	0.001	0.084	0.001
15	T/C	TBX2	gain	TBX2_2	0.001	0.066	0.001
16	T/C	PBX	gain	TBX2_2	0.001	0.066	0.001
17	T/C	TBX5	gain	TBX5_4	0.000	0.496	0.001
18	T/C	PAX6	gain	TBX5_4	0.0001	0.496	0.001
19	T/C	NKX3-1	gain	NKX3-1_6	0.004	0.096	0.002
20	T/C	MEF2A	gain	NKX3-1_6	0.004	0.096	0.002
21	T/C	NKX3-1	gain	NKX3-1_5	0.005	0.072	0.002
22	T/C	NKX2-5	gain	NKX3-1_5	0.005	0.072	0.002
23	T/C	NKX3-2	gain	NKX3-2_3	0.001	0.098	0.002
24	T/C	SPDEF	gain	NKX3-2_3	0.001	0.098	0.002
25	T/C	TBX15	gain	TBX15_1	0.0004	0.205	0.002

26	T/C	DMRTA1	gain	TBX15_1	0.0004	0.205	0.002
27	T/C	AHR::ARNT	gain	AHR::ARNT_1	0.004	0.230	0.006
28	T/C	FOXK1	gain	AHR::ARNT_1	0.004	0.230	0.006
29	T/C	NKX2-3	gain	NKX2-3_3	0.002	0.688	0.006
30	T/C	BARHL1	gain	NKX2-3_3	0.002	0.688	0.006
31	T/C	MGA	gain	MGA_2	0.001	0.450	0.009
32	T/C	Pax6	gain	MGA_2	0.001	0.450	0.009
33	T/C	NKX3-2	gain	NKX3-2_1	0.001	0.294	0.009
34	T/C	MNT	gain	NKX3-2_1	0.001	0.294	0.009
35	T/C	CREB3L1	gain	CREB3L1_3	0.002	0.718	0.011
36	T/C	RXRA	gain	CREB3L1_3	0.002	0.718	0.011
37	T/C	NKX3-1	gain	NKX3-1_1	0.010	0.204	0.012
38	T/C	NKX2-3	gain	NKX2-3_2	0.010	0.274	0.012
39	T/C	MSX2	gain	NKX2-3_2	0.010	0.274	0.012
40	T/C	AHR::ARNT::HIF1A	gain	AHR::ARNT::HIF1A_1	0.006	0.264	0.013
41	T/C	FOXA	gain	AHR::ARNT::HIF1A_1	0.006	0.264	0.013
42	T/C	AHR::ARNT	gain	AHR::ARNT_3	0.005	0.318	0.014
43	T/C	MYC	gain	AHR::ARNT_3	0.005	0.318	0.014
44	T/C	Nkx3-2	gain	MA0122.1	0.006	0.256	0.015
45	T/C	VDR	gain	MA0122.1	0.006	0.256	0.015
46	T/C	TBX1	gain	TBX1_4	0.005	0.121	0.015
47	T/C	MSX1	gain	TBX1_4	0.005	0.121	0.015
48	T/C	TBX21	gain	TBX21_4	0.003	0.423	0.015
49	T/C	FOXD3	gain	TBX21_4	0.003	0.423	0.015
50	T/C	NKX3-1	gain	NKX3-1_3	0.009	0.343	0.021
51	T/C	PAX4	gain	NKX3-1_3	0.009	0.343	0.021
52	T/C	EN2	gain	EN2_3	0.005	0.793	0.021
53	T/C	FOXJ3	gain	EN2_3	0.005	0.793	0.021
54	T/C	NKX3-2	gain	NKX3-2_2	0.005	0.490	0.023
55	T/C	NR2E1	gain	NKX3-2_2	0.005	0.490	0.023
56	T/C	NKX2-6	gain	NKX2-6_1	0.006	0.395	0.029
57	T/C	FOXA2	gain	NKX2-6_1	0.006	0.395	0.029
58	T/C	ZNF784	gain	ZNF784_1	0.004	0.668	0.030
59	T/C	IKZF3	gain	ZNF784_1	0.004	0.668	0.030
60	T/C	MTF1	gain	MTF1_1	0.009	0.311	0.033
61	T/C	KLF12	gain	MTF1_1	0.009	0.311	0.033
62	T/C	CDX	gain	CDX_1	0.009	0.436	0.035
63	T/C	HNF1A	gain	CDX_1	0.009	0.436	0.035
64	T/C	ZNF75A	gain	ZNF75A_1	0.005	0.735	0.042
65	T/C	HNF4A	gain	ZNF75A_1	0.005	0.735	0.042
66	T/C	HESX1	gain	HESX1_1	0.013	0.746	0.043
67	T/C	ZNF232	loss	ZNF232_1	0.001	0.004	0.098
68	T/C	HDAC2	loss	ZNF232_1	0.001	0.004	0.098
69	T/C	FOXB1	loss	FOXB1_2	0.005	0.014	0.117
70	T/C	BARX1	loss	FOXB1_2	0.005	0.014	0.117

71	T/C	NANOG	loss	NANOG_disc 4	0.0003	0.002	0.118
72	T/C	NKX2-8	loss	NANOG_disc 4	0.0003	0.002	0.118
73	T/C	SMAD4	loss	SMAD4_1	0.001	0.010	0.135
74	T/C	TATA	loss	SMAD4_1	0.001	0.010	0.135
75	T/C	NR4A2	loss	NR4A2_4	0.010	0.009	0.136
76	T/C	SOX10	loss	NR4A2_4	0.010	0.009	0.136
77	T/C	MYEF2	loss	MYEF2_1	0.006	0.033	0.142
78	T/C	ESRRA	loss	MYEF2_1	0.006	0.033	0.142
79	T/C	FOXD2	loss	FOXD2_1	0.007	0.011	0.153
80	T/C	IRF1	loss	FOXD2_1	0.007	0.011	0.153
81	T/C	MEF2A	loss	MEF2A_3	0.002	0.019	0.157
82	T/C	IRF2	loss	MEF2A_3	0.002	0.019	0.157
83	T/C	MYEF2	loss	MYEF2_5	0.004	0.022	0.204
84	T/C	NR2F6	loss	MYEF2_5	0.004	0.022	0.204
85	T/C	HNF1B	loss	HNF1B_1	0.008	0.033	0.238
86	T/C	NKX2-8	loss	HNF1B_1	0.008	0.033	0.238
87	T/C	SOX10	loss	SOX10_7	0.004	0.046	0.248
88	T/C	MAX	loss	SOX10_7	0.004	0.046	0.248
89	T/C	IRF8	loss	IRF8_3	0.007	0.019	0.284
90	T/C	ESRRA	loss	IRF8_3	0.007	0.019	0.284
91	T/C	MYC::MAX	loss	MYC::MAX_5	0.005	0.032	0.320
92	T/C	BATF	loss	MYC::MAX_5	0.005	0.032	0.320
93	T/C	NOTO	loss	NOTO_1	0.010	0.036	0.418
94	T/C	MEF2	loss	MEF2_disc1	0.0002	0.002	0.440
95	T/C	HNF1B	loss	MEF2_disc1	0.0002	0.002	0.440
96	T/C	MEF2D	loss	MEF2D_1	0.003	0.025	0.461
97	T/C	SRY	loss	MEF2D_1	0.003	0.025	0.461
	C[8]	lymph vessel development (GO:0001945; FDR = 0.00186); positive regulation of BMP signaling pathway (GO:0030513; FDR = 0.00427); regulation of epithelial to mesenchymal transition (GO:0010717; FDR = 0.00415); lung development (GO:0030324; FDR = 0.0352).					
	T[9]	-					

1 – reference (Ref) / alternative (SNP) allele;
2 – TF - transcription factor;
3 – binding of TF to the reference (LOSS) / alternative (GAIN) allele;
4 – binding sites with high affinity for TF;
5 – p value statistically confirming the potential gain or loss of function of the genomic region with SNP in terms of transcription factor binding;
6 – p-value for assessing the binding of TF to the Ref allele;
7 – p-value for assessing the binding of TF to the SNP allele;
8 – biological processes pathogenetically significant for IS, in which TFs that bind to the SNP allele are jointly involved (data from the Gene Ontology resource; http://geneontology.org/);
9 – biological processes pathogenetically significant for IS, in which TFs that bind to reference allele are jointly involved (data from the Gene Ontology resource; http://geneontology.org/)

Table S16: Analysis of the effect of rs910652 *HSPA12B* on the binding of DNA to transcription factors

№	Ref/SNP allele[1]	TF[2]	GAIN /LOSS[3]	Motif[4]	P-Value SNP impact[5]	P-Value Ref[6]	P-Value SNP[7]
1	T/C	ZNF143	gain	ZNF143_disc1	0.014	0.054	0.003
2	T/C	STAT5A	gain	STAT5A_4	0.003	0.161	0.006
3	T/C	NFAT	gain	STAT5A_4	0.003	0.161	0.006
4	T/C	SMARC	gain	SMARC_disc2	0.007	0.058	0.006
5	T/C	RBPJ	gain	RBPJ_1	0.002	0.118	0.008
6	T/C	POU5F1	gain	RBPJ_1	0.002	0.118	0.008
7	T/C	SOX3	gain	SOX3_3	0.028	0.073	0.014
8	T/C	ZBTB33	gain	ZBTB33_disc1	0.008	0.097	0.014
9	T/C	SOX8	gain	SOX8_8	0.008	0.097	0.015
10	T/C	SOX8	gain	SOX8_3	0.018	0.124	0.021
11	T/C	CHD2	gain	CHD2_disc1	0.031	0.171	0.025
12	T/C	HDAC2	gain	HDAC2_disc1	0.036	0.229	0.025
13	T/C	ZNF75A	gain	ZNF75A_1	0.012	0.483	0.033
14	T/C	AP1	gain	AP1_disc5	0.002	0.347	0.034
15	T/C	RXRB	gain	AP1_disc5	0.002	0.347	0.034
16	T/C	ZBTB33	gain	MA0527.1	0.033	0.250	0.038
17	T/C	STAT3	gain	STAT3_2	0.028	0.454	0.040
18	T/C	ID4	gain	ID4_1	0.030	0.393	0.043
19	T/C	AR	gain	AR_6	0.015	0.617	0.043
20	T/C	HMX3	gain	HMX3_1	0.027	0.322	0.047
21	T/C	SPIC	gain	SPIC_2	0.009	0.668	0.047
22	T/C	IRF2	loss	IRF2_1	0.009	0.014	0.078
23	T/C	FOXG1	loss	FOXG1_3	0.019	0.016	0.083
24	T/C	STAT5A	loss	STAT5A_2	0.005	0.004	0.105
25	T/C	FOXI1	loss	STAT5A_2	0.005	0.004	0.105
26	T/C	Hltf	loss	MA0109.1	0.034	0.016	0.115
27	T/C	HNF4	loss	HNF4_disc3	0.014	0.012	0.119
28	T/C	IRF2	loss	MA0051.1	0.0005	0.009	0.121
29	T/C	PAX6	loss	MA0051.1	0.0005	0.009	0.121
30	T/C	HNF4	loss	HNF4_disc1	0.037	0.023	0.126
31	T/C	NKX3-1	loss	NKX3-1_1	0.015	0.011	0.136
32	T/C	HNF4A	loss	HNF4A_1	0.027	0.029	0.147
33	T/C	HNF4A	loss	HNF4A_12	0.029	0.035	0.168
34	T/C	HNF4A	loss	HNF4A_7	0.027	0.025	0.178
35	T/C	IRF1	loss	IRF1_1	0.001	0.014	0.181
36	T/C	EBF1	loss	IRF1_1	0.001	0.014	0.181
37	T/C	HOXD13	loss	HOXD13_5	0.031	0.030	0.206
38	T/C	IRF2	loss	IRF2_2	0.021	0.033	0.210
39	T/C	MYC	loss	MYC_1	0.024	0.028	0.213
40	T/C	Pax4	loss	MA0068.1	0.004	0.028	0.217
41	T/C	HMX2	loss	MA0068.1	0.004	0.028	0.217
42	T/C	HNF4A	loss	HNF4A_2	0.004	0.016	0.229
43	T/C	IRF1	loss	HNF4A_2	0.004	0.016	0.229
44	T/C	BBX	loss	BBX_1	0.018	0.039	0.238
45	T/C	MYEF2	loss	MYEF2_2	0.019	0.038	0.239
46	T/C	ZBTB49	loss	ZBTB49_1	0.0002	0.009	0.246
47	T/C	HNF4A	loss	ZBTB49_1	0.0002	0.009	0.246

48	T/C	PAX4	loss	PAX4_5	0.007	0.035	0.259
49	T/C	HNF4A	loss	MA0114.2	0.010	0.030	0.299
50	T/C	POU6F1	loss	POU6F1_1	0.021	0.045	0.327
51	T/C	IRF	loss	IRF_disc6	0.015	0.038	0.333
52	T/C	PPARG::RXRA	loss	MA0065.2	0.010	0.045	0.398
53	T/C	PPARG	loss	PPARG_4	0.011	0.045	0.406
54	T/C	HOXB13	loss	HOXB13_2	0.013	0.034	0.409
55	T/C	E2F8	loss	E2F8_1	0.007	0.045	0.436
56	T/C	RXRG	loss	RXRG_3	0.012	0.034	0.483
57	T/C	FOXO3	loss	FOXO3_5	0.015	0.026	0.532
58	T/C	NR2F6	loss	NR2F6_5	0.016	0.035	0.557
59	T/C	HNF4A	loss	HNF4A_4	0.002	0.015	0.907
60	T/C	MYC	loss	HNF4A_4	0.002	0.015	0.907
	C[8]	interleukin-9-mediated signaling pathway (GO:0038113; FDR = 0.00454); interleukin-2-mediated signaling pathway (GO:0038110; FDR = 0.00569); growth hormone receptor signaling pathway via JAK-STAT (GO:0060397; FDR = 0.00755); interleukin-15-mediated signaling pathway (GO:0035723; FDR = 0.00859); cellular response to estrogen stimulus (GO:0071391; FDR = 0.0216); positive regulation of epithelial cell proliferation (GO:0050679; FDR = 0.00004); cellular response to transforming growth factor beta stimulus (GO:0071560; FDR = 0.000426).					
	T[9]	cellular response to prostaglandin E stimulus (GO:0071380; FDR = 0.027); cellular response to hypoxia (GO:0071456; FDR = 0.00344); response to cytokine (GO:0034097; FDR = 0.0407).					
1 – reference (Ref) / alternative (SNP) allele;<br>2 – TF - transcription factor;<br>3 – binding of TF to the reference (LOSS) / alternative (GAIN) allele;<br>4 – binding sites with high affinity for TF;<br>5 – p value statistically confirming the potential gain or loss of function of the genomic region with SNP in terms of transcription factor binding;<br>6 – p-value for assessing the binding of TF to the Ref allele;<br>7 – p-value for assessing the binding of TF to the SNP allele;<br>8 – biological processes pathogenetically significant for IS, in which TFs that bind to the SNP allele are jointly involved (data from the Gene Ontology resource; http://geneontology.org/);<br>9 – biological processes pathogenetically significant for IS, in which TFs that bind to reference allele are jointly involved (data from the Gene Ontology resource; http://geneontology.org/)							

Table S17. Analysis of the effect of rs7189628 *DNAJA2* on the binding of DNA to transcription factors

№	Ref/SNP allele[1]	TF[2]	GAIN /LOSS[3]	Motif[4]	P-Value SNP impact[5]	P-Value Ref[6]	P-Value SNP[7]
1	T/C	SPI1	gain	SPI1_disc3	0.0001	0.060	0.002
2	T/C	SIN3A	gain	SPI1_disc3	0.0001	0.060	0.002
3	T/C	PAX4	gain	PAX4_1	0.001	0.265	0.003
4	T/C	HLF	gain	PAX4_1	0.001	0.265	0.003
5	T/C	ELF2	gain	ELF2_1	0.010	0.053	0.004
6	T/C	POU2F2	gain	POU2F2_disc2	0.0001	0.078	0.004
7	T/C	BCL	gain	POU2F2_disc2	0.0001	0.078	0.004

8	T/C	TCF12	gain	TCF12_1	0.005	0.058	0.005
9	T/C	NFIL3	gain	TCF12_1	0.005	0.058	0.005
10	T/C	EGR1	gain	EGR1_disc3	0.001	0.086	0.005
11	T/C	ELF3	gain	EGR1_disc3	0.001	0.086	0.005
12	T/C	CTCF	gain	CTCF_disc5	0.003	0.056	0.005
13	T/C	GABP	gain	CTCF_disc5	0.003	0.056	0.005
14	T/C	YY1	gain	YY1_disc5	0.001	0.160	0.006
15	T/C	NFE2	gain	YY1_disc5	0.001	0.160	0.006
16	T/C	ELF1	gain	ELF1_disc2	0.001	0.200	0.008
17	T/C	ETS1	gain	ELF1_disc2	0.001	0.200	0.008
18	T/C	ESRRA	gain	ESRRA_disc4	0.002	0.090	0.008
19	T/C	GABPA	gain	ESRRA_disc4	0.002	0.090	0.008
20	T/C	NFE2L2	gain	NFE2L2_1	0.000	0.165	0.009
21	T/C	NFE2L2	gain	NFE2L2_1	0.000	0.165	0.009
22	T/C	TRIM28	gain	TRIM28_disc2	0.005	0.110	0.010
23	T/C	E2F1	gain	TRIM28_disc2	0.005	0.110	0.010
24	T/C	ELF1	gain	MA0473.1	0.001	0.237	0.010
25	T/C	E2F1	gain	MA0473.1	0.001	0.237	0.010
26	T/C	ZEB1	gain	ZEB1_3	0.008	0.101	0.010
27	T/C	TFAP2A	gain	ZEB1_3	0.008	0.101	0.010
28	T/C	CTCF	gain	CTCF_disc8	0.001	0.158	0.011
29	T/C	MYB	gain	CTCF_disc8	0.001	0.158	0.011
30	T/C	GABPA	gain	GABPA_3	0.003	0.320	0.012
31	T/C	E2F4	gain	GABPA_3	0.003	0.320	0.012
32	T/C	EGR1	gain	EGR1_disc2	0.001	0.257	0.013
33	T/C	ELK1	gain	EGR1_disc2	0.001	0.257	0.013
34	T/C	ELK4	gain	MA0076.2	0.005	0.153	0.014
35	T/C	E2F1	gain	MA0076.2	0.005	0.153	0.014
36	T/C	BCL	gain	BCL_disc10	0.002	0.149	0.014
37	T/C	Erg	gain	BCL_disc10	0.002	0.149	0.014
38	T/C	ETS	gain	ETS_disc2	0.004	0.282	0.016
39	T/C	ERF	gain	ETS_disc2	0.004	0.282	0.016
40	T/C	ELF1	gain	ELF1_disc1	0.002	0.220	0.016
41	T/C	FLI1	gain	ELF1_disc1	0.002	0.220	0.016
42	T/C	NRF1	gain	NRF1_1	0.006	0.167	0.018
43	T/C	DMRTA1	gain	NRF1_1	0.006	0.167	0.018
44	T/C	ZBTB3	gain	ZBTB3_1	0.005	0.302	0.019
45	T/C	SRY	gain	ZBTB3_1	0.005	0.302	0.019
46	T/C	ETS1	gain	ETS1_6	0.009	0.715	0.020
47	T/C	EHF	gain	MA0598.1	0.004	0.254	0.027
48	T/C	RUNX2	gain	MA0598.1	0.004	0.254	0.027
49	T/C	E2F1	gain	E2F1_14	0.010	0.232	0.028
50	T/C	ETS1	gain	ETS1_5	0.004	0.316	0.030
51	T/C	YY1	gain	ETS1_5	0.004	0.316	0.030
52	T/C	TEAD2	gain	TEAD2_1	0.003	0.363	0.032
53	T/C	ZBTB14	gain	TEAD2_1	0.003	0.363	0.032
54	T/C	NFE2	gain	NFE2_disc3	0.003	0.249	0.036
55	T/C	RXRA	gain	NFE2_disc3	0.003	0.249	0.036
56	T/C	TFAP4	gain	TFAP4_1	0.005	0.265	0.036
57	T/C	HLF	gain	TFAP4_1	0.005	0.265	0.036

58	T/C	TFAP4	gain	TFAP4_4	0.007	0.346	0.037
59	T/C	E2F1	gain	TFAP4_4	0.007	0.346	0.037
60	T/C	PAX5	gain	PAX5_disc5	0.010	0.635	0.042
61	T/C	HDAC2	gain	HDAC2_disc5	0.008	0.314	0.044
62	T/C	INSM1	gain	HDAC2_disc5	0.008	0.314	0.044
63	T/C	CTCF	gain	CTCF_disc10	0.009	0.301	0.045
64	T/C	AP1	gain	AP1_disc10	0.010	0.328	0.049
65	T/C	ZBTB7A	loss	ZBTB7A_disc2	0.001	0.001	0.056
66	T/C	E2F	loss	ZBTB7A_disc2	0.001	0.001	0.056
67	T/C	Ddit3::Cebpa	loss	MA0019.1	0.002	0.005	0.087
68	T/C	E2F1	loss	MA0019.1	0.002	0.005	0.087
69	T/C	HES1	loss	HES1_1	0.009	0.013	0.118
70	T/C	T	loss	T_1	0.008	0.022	0.134
71	T/C	NR3C1	loss	T_1	0.008	0.022	0.134
72	T/C	SCRT1	loss	SCRT1_1	0.000	0.001	0.177
73	T/C	Bach1::Mafk	loss	SCRT1_1	0.000	0.001	0.177
74	T/C	RFX1	loss	RFX1_3	0.004	0.012	0.181
75	T/C	AP1	loss	RFX1_3	0.004	0.012	0.181
76	T/C	SCRT2	loss	SCRT2_1	0.000	0.001	0.231
77	T/C	HIF1A	loss	SCRT2_1	0.000	0.001	0.231
78	T/C	T	loss	T_2	0.005	0.020	0.273
79	T/C	JUN	loss	T_2	0.005	0.020	0.273
80	T/C	HLF	loss	HLF_1	0.007	0.010	0.276
81	T/C	RFX1	loss	HLF_1	0.007	0.010	0.276
82	T/C	ATF4	loss	ATF4_3	0.008	0.022	0.292
83	T/C	Atoh1	loss	ATF4_3	0.008	0.022	0.292
84	T/C	FOXP3	loss	FOXP3_1	0.002	0.048	0.338
85	T/C	GATA	loss	FOXP3_1	0.002	0.048	0.338
86	T/C	Sox2	loss	MA0143.3	0.009	0.047	0.366
87	T/C	LEF1	loss	LEF1_2	0.003	0.024	0.366
88	T/C	E2F1	loss	LEF1_2	0.003	0.024	0.366
89	T/C	SOX17	loss	SOX17_1	0.009	0.049	0.641
90	T/C	TFAP2E	loss	SOX17_1	0.009	0.049	0.641
91	T/C	BRCA1	loss	BRCA1_1	0.003	0.012	0.887
92	T/C	NR1H	loss	BRCA1_1	0.003	0.012	0.887
	C[8]	cellular response to reactive oxygen species (GO:0034614; FDR = 0.029); lymphocyte differentiation (GO:0030098; FDR = 0.0000708); lymphocyte activation (GO:0046649; FDR = 0.00238); leukocyte activation (GO:0045321; FDR = 0.00958).					
	T[9]	negative regulation of interleukin-4 production (GO:0032713; FDR = 0.00301); negative regulation of interleukin-5 production (GO:0032714; FDR = 0.0036); lymphocyte proliferation (GO:0046651; FDR = 0.0227); positive regulation by host of viral transcription (GO:0043923; FDR = 0.0139); positive regulation of vascular endothelial growth factor production (GO:0010575; FDR = 0.000842); cytokine production (GO:0001816; FDR = 0.0282); B cell homeostasis (GO:0001782; FDR = 0.0291); epithelial cell apoptotic process (GO:1904019; FDR = 0.00339); regulation of					

		transforming growth factor beta production (GO:0071634; FDR = 0.047); transforming growth factor beta receptor superfamily signaling pathway (GO:0141091; FDR = 0.0407); cellular response to hypoxia (GO:0071456; FDR = 0.0238); positive regulation of cell-cell adhesion (GO:0022409; FDR = 0.0229); response to oxidative stress (GO:0006979; FDR = 0.0324); leukocyte differentiation (GO:0002521; FDR = 0.0497); cellular response to cytokine stimulus (GO:0071345; FDR = 0.0417).
1 – reference (Ref) / alternative (SNP) allele;<br>2 – TF - transcription factor;<br>3 – binding of TF to the reference (LOSS) / alternative (GAIN) allele;<br>4 – binding sites with high affinity for TF;<br>5 – p value statistically confirming the potential gain or loss of function of the genomic region with SNP in terms of transcription factor binding;<br>6 – p-value for assessing the binding of TF to the Ref allele;<br>7 – p-value for assessing the binding of TF to the SNP allele;<br>8 – biological processes pathogenetically significant for IS, in which TFs that bind to the SNP allele are jointly involved (data from the Gene Ontology resource; http://geneontology.org/);<br>9 – biological processes pathogenetically significant for IS, in which TFs that bind to reference allele are jointly involved (data from the Gene Ontology resource; http://geneontology.org/)		

Table S18: Baseline and clinical characteristics of the studied groups.

Baseline and clinical characteristics		COVID-19 patients (n=199)	Controls (n=962)	P-value
Age, Me [Q1; Q3]		68 [57; 78]	56 [52; 63]	**<0.001**
BMI, Me [Q1; Q3]		30 [24.2; 35.1]	27.6 [25; 31] (n=302)	**<0.05**
Gender	Males, N (%)	92 (46.2%)	425 (41.3%)	**>0.05**
	Females, N (%)	107 (53.8%)	604 (58.7%)	
Smoking	Yes, N (%)	56 (28%)	283 (27.5%)	**>0.05**
	No, N (%)	137 (69%)	729 (70.8%)	
	ND, N (%)	6 (3%)	17 (1.7%)	
Low physical activity	Yes, N (%)	106 (53.3%)	ND	
	No, N (%)	87 (43.7%)		
	ND, N (%)	6 (3%)		
Low fruit/vegetable consumption	Yes, N (%)	126 (63.3%)	ND	
	No, N (%)	67 (33.7%)		
	ND, N (%)	6 (3%)		
Vaccination	Yes, N (%)	21 (10.6%)	ND	
	No, N (%)	20 (10.1%)		
	ND, N (%)	146 (73.4%)		
Death	Yes, N (%)	67 (33.7%)	0 (0%)	**<0.001**
	No, N (%)	132 (66.3%)	1029 (100%)	
Essential hypertension (EH)	Yes, N (%)	129 (64.8%)	195 (19%)	**<0.001**
	No, N (%)	70 (39.7%)	834 (81%)	
	Yes, N (%)	84 (42.2%)	0 (0%)	**<0.001**

Coronary artery disease (CAD)	No, N (%)	115 (57.8%)	1029 (100%)	
Atrial fibrillation (AF)	Yes, N (%)	42 (21.1%)	0 (0%)	**<0.001**
	No, N (%)	158 (79.4%)	1029 (100%)	
Congestive heart failure (CHF)	Yes, N (%)	71 (35.7%)	0 (0%)	**<0.001**
	No, N (%)	129 (64.8%)	1029 (100%)	
Cerebrovascular accident (CVA)	Yes, N (%)	32 (16.1%)	0 (0%)	**<0.001**
	No, N (%)	167 (83.9%)	1029 (100%)	
Diabetes mellitus type 2 (T2D)	Yes, N (%)	39 (19.6%)	0 (0%)	**<0.001**
	No, N (%)	160 (80.4%)	1029 (100%)	
Bed-day in intensive care unit (ICU), Me [Q1; Q3]		10 (7; 14) (n=199)	-	
Leukocytes, Me [Q1; Q3]		12.3 (8.9; 15.7) (n=199)	ND	
Platelets, Me [Q1; Q3]		261 (193; 314) (n=199)	ND	
Prothrombin index (PTI), Me [Q1; Q3]		83 [74; 92] (n=198)	ND	
Activated partial thromboplastin time (APTT), Me [Q1; Q3]		28.5 [25.2; 33.19] (n=199)	ND	
Fibrinogen, Me [Q1; Q3]		5.12 [4.1; 6.19] (n=41)	ND	
C-reactive protein (CRP), Me [Q1; Q3]		98.5 [45.7; 146] (n=199)	ND	
Ground-glass opacity, admission, Me [Q1; Q3]		45 [20; 60] (n=199)	-	
Ground-glass opacity, discharge, Me [Q1; Q3]		38 [10; 50] (n=187)	-	
CT dynamics	Positive. N (%)	136 (68.34%)	-	
	Negative. N (%)	52 (26.13%)		
	ND. N (%)	11 (5.53%)		
Oxygen therapy day, Me [Q1; Q3]		3 [0; 5] (n=199)	-	
Non-invasive mechanical ventilation, Me [Q1; Q3]		5 [2; 8] (n=199)	ND	
Time to the start of clot growth (T lag, min), Me [Q1; Q3]		1 [0.8; 1.1] (n=127)	ND	
Initial spatial clot growth rates (Vi, µm/min), Me [Q1; Q3]		39.9 [21.9; 53.3] (n=127)	ND	
Stationary spatial clot growth rates (Vs, µm/min), Me [Q1; Q3]		10.7 [6; 24.2] (n=127)	ND	
Stationary spatial clot growth rates (V, µm/min), Me [Q1; Q3]		11.5 [6.7; 25.9] (n=127)	ND	
Maximum optical density of the formed clot (D), Me [Q1; Q3]		27973 [22874; 32146] (n=127)	ND	

Clot size at 30 min after coagulation activation (CS, μm), Me [Q1; Q3]	670 [405; 1052] (n=127)	ND	
Statistically significant differences between groups are indicated in bold; ND – no data.			

Table S19: Primers and probes designed for the study.

SNPs	Probes and Primers
rs862832 *HSPA12B*	F 5′-TGTGGTGGTGGCAGCTAC-3′<br>R 5′-CAGCATTTCAGGGCAGGAC-3′<br>5′-(FAM)CAGGGCTACTC**C**ACTCCCTCC(RTQ1)-3′<br>5′-(ROX)CAGGGCTACTC**T**ACTCCCTCC(BHQ2)-3′
rs910652 *HSPA12B*	F 5′-ATCTCAAGCCTGCCTGTGG-3′<br>R 5′-CCGTCCTGATCTTTGCACAG-3′<br>5′-(FAM)CTACACTT**T**TCACTGGAAG(RTQ1)-3′<br>5′-(ROX)CTACACTT**C**TCACTGGAAG(BHQ2)-3′
rs17155992 *HSPA14*	F 5′-TCCTTCAGCCCTGTAATGATGT-3′<br>R 5′-GGCTTCAGTATCAACCATCTTCT-3′<br>5′-(FAM)CTAACATTATGAC**G**TTGTGTCTTC(RTQ1)-3′<br>5′-(ROX)CTAACATTATGAC**A**TTGTGTCTTC(BHQ2)-3′
rs1043618 *HSPA1A*	F 5′-ATCCAGTGTTCCGTTTCCAG-3′<br>R 5′-GAGTAGGTGGTGCCCAGGT-3′<br>5′-(FAM)CTCAGAGC**G**GAGCCGAC(RTQ1)-3′<br>5′-(ROX)CTCAGAGC**C**GAGCCGAC(BHQ2)-3′
rs6457452 *HSPA1B*	F 5′-TCAGAAGGGGAAAGGCGG-3′<br>R 5′-GGAAAGCCTTGGGACCGC-3′<br>5′-(FAM)CGAGGGTCCG**C**TTCGTCTTTC(RTQ1)-3′<br>5′-(ROX)CGAGGGTCCG**T**TTCGTCTTTC(BHQ2)-3′
rs13161158 *HSPA4*	F ′- CCAGTGGCTGATACACCAGA-3′<br>R 5′-GGAAGACACTTACGCATTCCA-3′<br>5′-(FAM)CCATCTAG**T**GCCCCTAGCT(RTQ1)-3′<br>5′-(ROX) CCATCTAG**C**GCCCCTAGCT(BHQ2)-3′
rs753856 *HSPA6*	F 5′-TGAGGATGAGGCCCAGAGG-3′<br>R 5′-AGCCAGGCAAGGACTTCC-3′<br>5′-(FAM)CCTTAGGGA**C**AAGATTCC(RTQ1)-3′<br>5′-(ROX)CCTTAGGGA**G**AAGATTCC(BHQ2)-3′
rs1042665 *HSPA9*	F 5′-AAATAAGCTCCGGCTGAAAA-3′<br>R 5′-GACAGGGGTTGATTTGACTAAA-3′<br>5′-(FAM)AGTTCACA**T**TTAGCCT(RTQ1)-3′<br>5′-(ROX)AGTTCACA**C**TTAGCCT(BHQ2)-3′
rs706121 *BAG1*	F 5′- CAGCCCTCTCTCATCACCTT -3′<br>R 5′- TTCCAGTTCAGGAGCCTCTC -3′<br>5′- FAM- CTGAACCTT**C**CTTTCCAGTC-RTQ1-3′<br>5′- ROX- CTGAACCTT**T**CTTTCCAGTC-BHQ2-3′
rs196329 *BAG3*	F 5′- AGTGTCCACCACACCCTTTC -3′<br>R 5′- GGTTCAGCATGGGCAAAG-3′<br>5′- FAM- TCACCTTCC**A**CTCAGTTAC-RTQ1-3′<br>5′- ROX- TCACCTTCC**G**CTCAGTTAC-BHQ2-3′
rs196336 *BAG3*	F 5′- CACCTGCAGTCTTCCTGGAT -3′<br>R 5′- CATCCGGCCTCTGTTTATTC -3′<br>5′- FAM- CAGGCCC**T**TTCCTGGC-RTQ1-3′<br>5′- ROX- CAGGCCC**C**TTCCTGGC -BHQ2-3′
